# The effect of harmonization on the variability of PET radiomic features extracted using various segmentation methods

**DOI:** 10.1007/s12149-024-01923-7

**Published:** 2024-04-04

**Authors:** Seyyed Ali Hosseini, Isaac Shiri, Pardis Ghaffarian, Ghasem Hajianfar, Atlas Haddadi Avval, Milad Seyfi, Stijn Servaes, Pedro Rosa-Neto, Habib Zaidi, Mohammad Reza Ay

**Affiliations:** 1 Translational Neuroimaging Laboratory, The McGill University Research Centre for Studies in Aging, Douglas Hospital, McGill University, Montréal, QC Canada; 2 Department of Neurology and Neurosurgery, Faculty of Medicine, McGill University, Montréal, QC Canada; 3 Division of Nuclear Medicine and Molecular Imaging, Geneva University Hospital, 1211 Geneva 4, Switzerland; 4 Chronic Respiratory Diseases Research Center, National Research Institute of Tuberculosis and Lung Diseases (NRITLD), Shahid Beheshti University of Medical Sciences, Tehran, Iran; 5 PET/CT and Cyclotron Center, Masih Daneshvari Hospital, Shahid Beheshti University of Medical Sciences, Tehran, Iran; 6 School of Medicine, Mashhad University of Medical Sciences, Mashhad, Iran; 7 Department of Medical Physics and Biomedical Engineering, Tehran University of Medical Sciences, Tehran, Iran; 8 Research Center for Molecular and Cellular Imaging, Tehran University of Medical Sciences, Tehran, Iran; 9 Department of Nuclear Medicine and Molecular Imaging, University of Groningen, University Medical Center Groningen, 9700 RB Groningen, Netherlands; 10 Department of Nuclear Medicine, University of Southern Denmark, 500 Odense, Denmark; 11 University Research and Innovation Center, Óbudabuda University, Budapest, Hungary

**Keywords:** PET, Radiomics, Segmentation, Lung cancer, Harmonization, Robustness

## Abstract

**Purpose:**

This study aimed to examine the robustness of positron emission tomography (PET) radiomic features extracted via different segmentation methods before and after ComBat harmonization in patients with non-small cell lung cancer (NSCLC).

**Methods:**

We included 120 patients (positive recurrence = 46 and negative recurrence = 74) referred for PET scanning as a routine part of their care. All patients had a biopsy-proven NSCLC. Nine segmentation methods were applied to each image, including manual delineation, K-means (KM), watershed, fuzzy-C-mean, region-growing, local active contour (LAC), and iterative thresholding (IT) with 40, 45, and 50% thresholds. Diverse image discretizations, both without a filter and with different wavelet decompositions, were applied to PET images. Overall, 6741 radiomic features were extracted from each image (749 radiomic features from each segmented area). Non-parametric empirical Bayes (NPEB) ComBat harmonization was used to harmonize the features. Linear Support Vector Classifier (LinearSVC) with L1 regularization For feature selection and Support Vector Machine classifier (SVM) with fivefold nested cross-validation was performed using StratifiedKFold with ‘n_splits’ set to 5 to predict recurrence in NSCLC patients and assess the impact of ComBat harmonization on the outcome.

**Results:**

From 749 extracted radiomic features, 206 (27%) and 389 (51%) features showed excellent reliability (ICC ≥ 0.90) against segmentation method variation before and after NPEB ComBat harmonization, respectively. Among all, 39 features demonstrated poor reliability, which declined to 10 after ComBat harmonization. The 64 fixed bin widths (without any filter) and wavelets (LLL)-based radiomic features set achieved the best performance in terms of robustness against diverse segmentation techniques before and after ComBat harmonization. The first-order and GLRLM and also first-order and NGTDM feature families showed the largest number of robust features before and after ComBat harmonization, respectively. In terms of predicting recurrence in NSCLC, our findings indicate that using ComBat harmonization can significantly enhance machine learning outcomes, particularly improving the accuracy of watershed segmentation, which initially had fewer reliable features than manual contouring. Following the application of ComBat harmonization, the majority of cases saw substantial increase in sensitivity and specificity.

**Conclusion:**

Radiomic features are vulnerable to different segmentation methods. ComBat harmonization might be considered a solution to overcome the poor reliability of radiomic features.

**Supplementary Information:**

The online version contains supplementary material available at 10.1007/s12149-024-01923-7.

## Introduction

Cancer is a leading cause of death worldwide and a significant barrier to increasing life expectancy [1]. According to the 2019 report World Health Organization (WHO) [2], the first or second primary reason for mortality in people under 70 is cancer in most parts of the world. A total of 19.3 and 10 million cancer incidences and cancer-related deaths occurred in 2020, respectively [3]. Among the different cancer types, the second most frequent type of cancer is lung cancer, with 2,206,771 new cases (11.4% of all cancers) and the highest number of deaths, with 1,796,144 patients (18% of all) [3].

In recent years, positron emission tomography (PET) imaging has become widely used for cancer diagnosis and is currently considered the gold standard for detecting solitary pulmonary nodules [4]. In recent years, radiomics, a computational approach using data mining techniques, received considerable attention in medical imaging analysis, particularly in the context of clinical oncology. Previous studies proved the capability of radiomics as a powerful tool in clinical diagnosis [5], prognosis, and outcome prediction of cancer and other diseases [6, 7] using statistical analyses [8], machine learning classifiers [9–11], and deep neural networks [12, 13].

A considerable number of radiomic features are extracted [14], leading to the development of multiple pipelines and packages for their extraction, with many of them being compliant with the Image Biomarker Standardization Initiative (IBSI) guidelines [15]. This includes the Pyradiomics package in Python [16] and SERA in MATLAB [17]. Nevertheless, radiomic features suffer from low repeatability (the degree to which the same measurement or computation yields the same results under identical conditions) and reproducibility (the results can be duplicated when the experiment or measurement is repeated under different conditions) since a combination of various factors [18, 19], including image acquisition [20], reconstruction algorithms [21, 22], image pre-processing [23], and image segmentation methods [18], highly affect radiomic features. Among these factors, different segmentation methods may impact the radiomic features to a large extent [18]. Although the effect of contouring variability has been recognized for its large impact on PET radiomic features [24], research focusing on the reproducibility of PET radiomic features with respect to variability of segmentation methods is relatively limited, especially when considering the influence of other factors, including image reconstruction technique and variability arising from the use of multi-center studies [25, 26]. Hence, a comprehensive examination of the impact of various segmentation methods, including manual, semi-automatic, and fully automated segmentation, on the extracted radiomics features is still required. Yang et al. [27] examined PET radiomics variability affected by manual segmentations in lung cancer. They concluded that the Gray Level Dependence Matrix (GLDM) family showed the highest performance in terms of reproducibility and harmonization against contouring variability.

Several potential solutions have been reported to overcome the low reproducibility of radiomic features against various influencing factors. Among these solutions, selecting robust features [19] and ComBat harmonization [28] have demonstrated a promising ability to boost the repeatability and reproducibility of the extracted radiomic features. To date, a few studies assessed the impact of automated segmentation on PET radiomic features [29, 30]. In the context of ComBat harmonization, the alignment of radiomic features is anticipated because of its inherent computational approach. While this outcome aligns with expectations, it is important to show that such harmonization also affects classification outcomes and how it is going to be.

This study aims to examine how various segmentation strategies, including automated, semi-automated, and manual contouring, affect PET radiomic features in lung cancer. A harmonization method was applied to reduce the variability of the features and calculated the intraclass correlation coefficient (ICC) for each radiomic feature with and without considering the harmonization effect to select robust features against various segmentation methods and endorse the impact of harmonization on the variability of the features. In addition, to examine the impact of ComBat harmonization on the performance of a machine learning (ML) classifier, Support Vector Machine (SVM) with a Linear Support Vector Classifier (LinearSVC) feature selection technique was implemented to predict the recurrence in NSCL subjects.

## Materials and methods

### Patients’ demographics and PET/CT data acquisition

Our study used PET images from a cohort of 120 patients, with 46 being recurrence positive and 74 being recurrence negative. These data were gathered from March 2018 to April 2021. The local ethics committee approved the study protocol and consent forms were waived given the retrospective nature of the study. All patients had biopsy-proven non-small cell lung cancer (NSCLC), and positive or negative recurrence. They underwent PET/CT scanning [GE Discovery 690 PET/CT scanner (General Electric Healthcare, USA)] as part of their routine workup. Patients fasted for at least 6 h prior to injection of ^18^F-Fluorodeoxyglucose (^18^F-FDG). The patients were instructed to fast for a minimum of 8 h prior to the scan. They were then administered an average activity of 309.26 MBq (138.90–572.25 MBq) of ^18^F-FDG. The average uptake time observed was 66.58 min (23.08–128.90 min). The acquisition time per bed position was consistently set at 3 min for all studies. Moreover, anatomical localization and attenuation correction were performed using low-dose CT imaging. PET data were reconstructed using the ordered subset expectation maximization (OSEM) iterative algorithm with 3 iterations and 18 subsets, leading to an image matrix of 256 × 256, with 3.906 mm^2^ pixel size. A Gaussian post-reconstruction filter with a full width at half maximum (FWHM) of 4.5 mm was applied. All the images were reconstructed using the same algorithm with the same number of iterations and subsets to minimize the effects of reconstruction on the results.

### Image segmentation

**Fig. 1:**
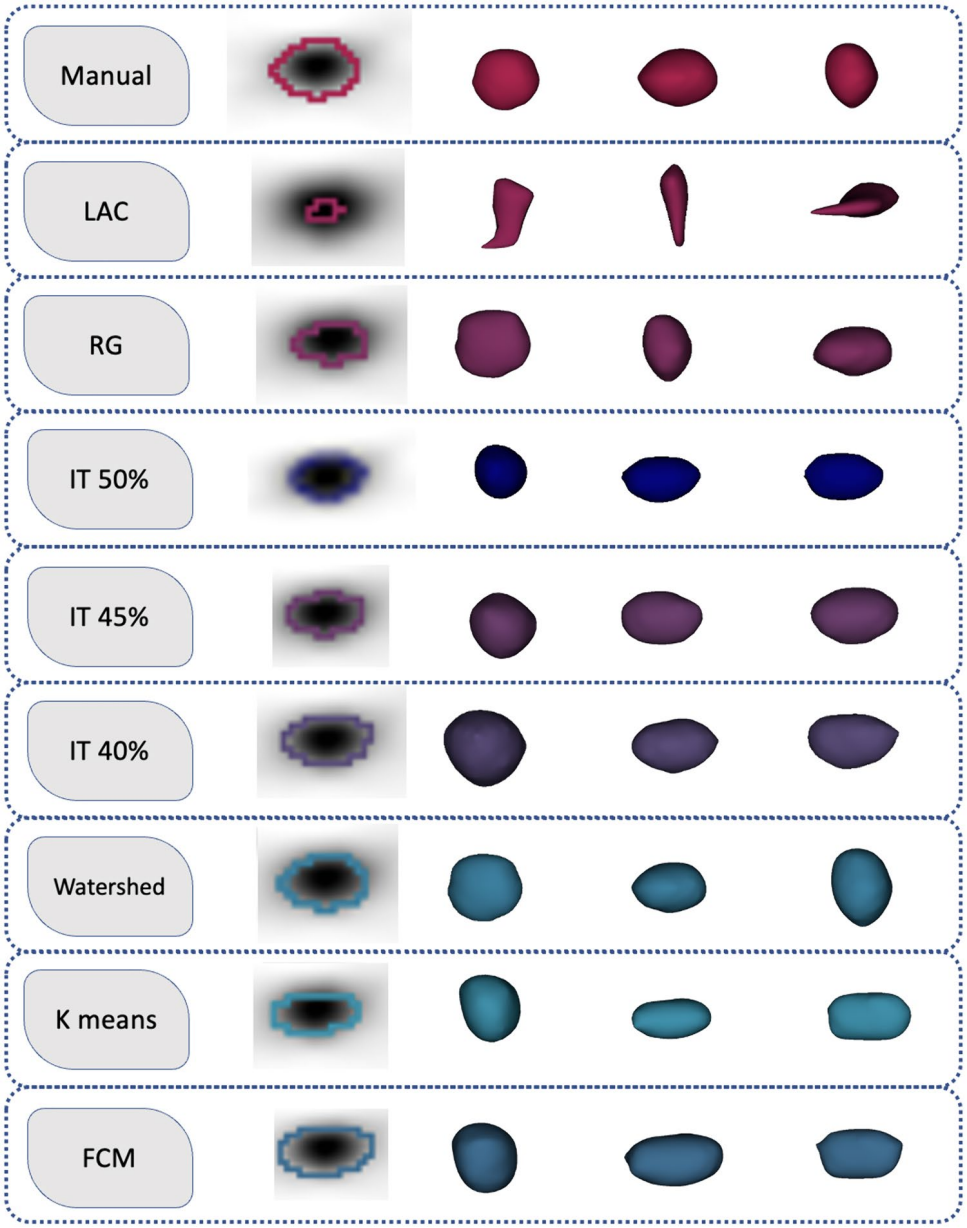
Multiple segmentation algorithms were applied in the current study. The first from the left is the central slice of PET images, the second is viewed anteriorly, the third is upright, and the fourth is downward

Numerous segmentation techniques were applied to PET images, ranging from manual contouring by a PET physicist with 15 years of experience (verified by another medical physicist with 10 years of experience), to semi-automated and automated methods, including K-means [31], watershed [32], FCM [33], iterative thresholding (IT)-based segmentation [34] with different thresholds (40, 45, and 50%), RG [35], and local active contour (LAC) [36] (Fig. 1).

In K-means segmentation, each image pixel is considered a feature point with a specific position. The basic K-means algorithm then randomly assigns this cluster site selection within a multidimensional subspace. Every spot is assigned to the cluster with the subjective mean vector adjacent to it. The process is continued until the positioning of the class mean vectors does not show a significant difference over the iterations [37].

FCM is based on a clustering approach whereby a sample is divided into multiple groups, upon each data point relating to each cluster to some extent. For instance, measurements at the center of a group will have a significant degree of membership in that cluster. In contrast, a data point considerably farther from a cluster would have a low grade of membership in that cluster [38].

The concept behind RG algorithms is that nearby pixels within a region have significant similarities. A typical operation involves comparing one pixel with its neighbors. If a similarity condition is met, the pixel can be assigned to any or all of the cluster’s neighbors. The choice of similarity criterion is critical, as noise impacts the outcome in every case [39].

The active contour model utilizes the image energy limitations and compels one to divide it into areas of interest. An active contour creates distinct boundaries or curves for each segment of the target item [40]. The contour is grouped into many categories depending on various requirements, including geometric models, gradient vector flow, and balloons [41].

The watershed segmentation algorithm is a well-established technique where the image is treated as a topographical landscape, with the grayscale intensity representing the height of the terrain. It identifies ‘markers’ or local minima in the landscape and floods the area from these marker points, and constructs ‘dam’ barriers at places of watershed lines, effectively segmenting the image into distinct regions [42].

To distinguish areas of interest within an image, IT, a complex image processing technique is employed that uses an iterative modification of a pixel intensity threshold. Beginning with an initial threshold, the procedure adjusts the threshold based on the histogram of pixel intensities in a cycle of repeated calculations until a convergence condition is satisfied, thus optimizing the separation of target and background regions. This dynamic approach handles a variety of image settings and contrasts and intensity fluctuations well [43].

### PET image pre-processing and feature extraction

**Fig. 2:**
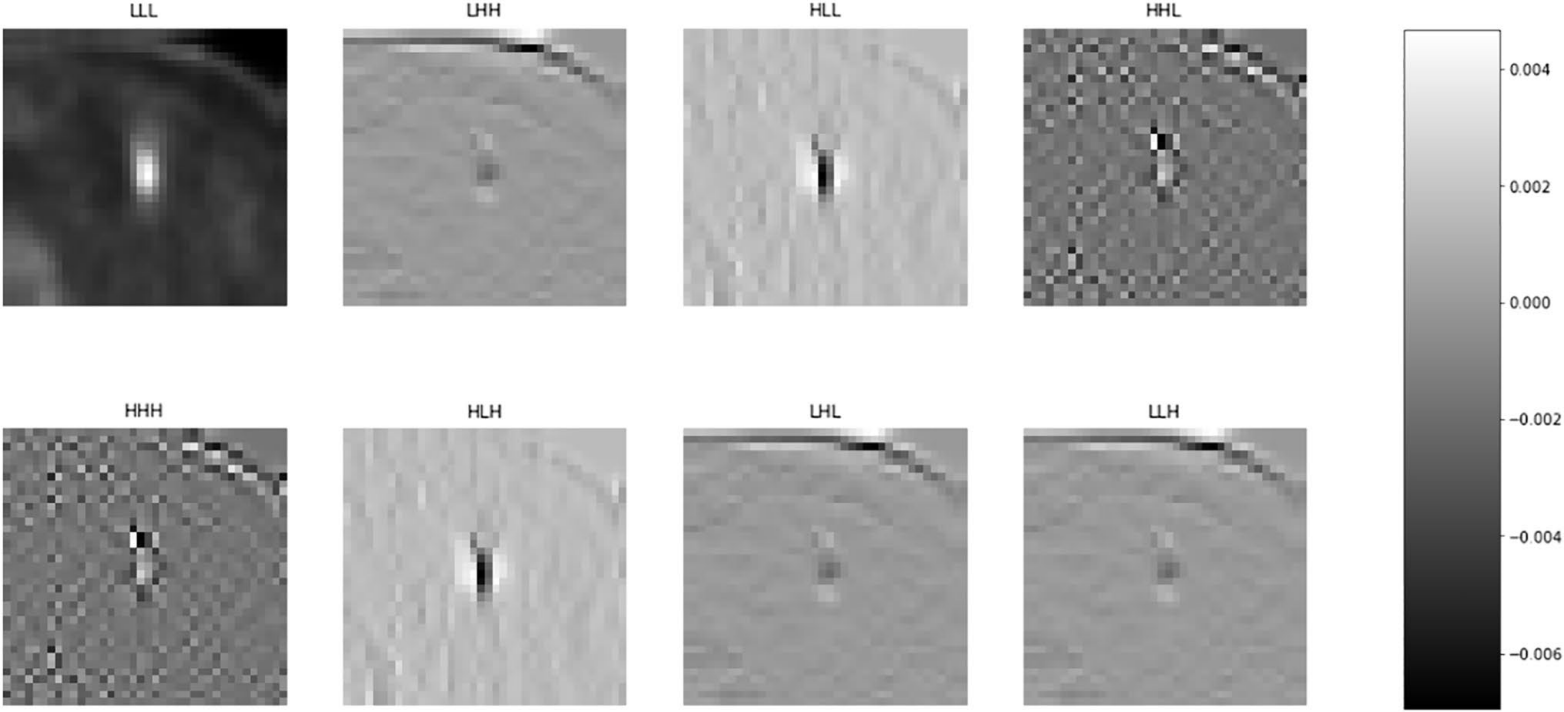
Representative example of wavelet images with multiple wavelet decompositions (LHH, HLL, HHL, LLL, HHH, HLH, LHL, and LLH) without any filter

The crucial step in this part involved the application of image discretization and wavelet transforms to pre-processed images. Image discretization, in this context, refers to the conversion of continuous image data into a finite set of intensity levels. This technique aids in reducing the complexity of the image data, thereby simplifying further analyses. Coif1 wavelet transformations were then employed to provide multi-resolution analysis of PET images. Wavelet decompositions, including LHH, HLL, LLL, HHL, HHH, HLH, LLH, and LHL, were used in this process (Fig. 2). These labels represent various forms of wavelet transform coefficients corresponding to different frequency bands and orientations: ‘L’ signifies low-pass filtered elements (approximations), and ‘H’ represents high-pass filtered features (details), in either horizontal, vertical, or diagonal directions. The application of these various wavelet decompositions allowed for the extraction of different texture features, capturing various elements of image heterogeneity.

From each region of interest (ROI) in each image with fixed 64 bin width and an isotropic voxel size of 1 × 1 × 1 mm^3^, 749 radiomic features were extracted using the Image Biomarker Standardization Initiative (IBSI) [15] compliant Pyradiomics package [16] in Python. These features consisted of four sets, including shape-, first-, second-, and higher-ordered features, comprising Gray Level Run Length Matrix (GLRLM), Gray Level Co-occurrence Matrix (GLCM), Gray Level Dependence Matrix (GLDM), and Gray Level Size Zone Matrix (GLSZM) feature sets. A total of 6741 radiomic features were extracted from each image, comprising 9 ROIs from 9 segmentation methods applied in the current study. The name, set, and family of the features are listed in Supplementary Table 1.

In our study, the response maps were created following the resampling process, adhering to the recommendations outlined by the IBSI. This approach ensures the maintenance of image data quality, especially when a higher-order interpolator, rather than a linear one, is employed during resampling. This process allows for more accurate wavelet response mapping, consequently resulting in more reliable and precise extraction of radiomic features.

### Non-parametric empirical Bayes (NPEB) ComBat harmonization

Before investigating the effect of different segmentation algorithms on radiomic features, non-parametric empirical Bayes (NPEB) harmonization was applied to the features. We used this harmonization method to decrease the features’ variability due to various segmentation methods explored in the current study [44]. NPEB ComBat harmonization is a widely used method initially developed for genomics to correct batch effects in high-dimensional data, but has been used in numerous fields, including PET radiomics analysis. At its core, ComBat uses an experimental Bayes framework to predict and adjust for variations across different batches or data sources (in this study, radiomic features). This is achieved using a model that estimates both the location (mean) and scale (variance) parameters for each batch, effectively normalizing the data across batches. The NPEB adaptation of ComBat extends this approach by not presuming a specific distribution for the data, which is particularly beneficial in diverse datasets, such as those in PET radiomics. This lack of assumption regarding data distribution makes NPEB ComBat more flexible and applicable to a wider range of scenarios [44]. In the context of PET radiomics, NPEB ComBat harmonization makes sure that features extracted from images acquired under different conditions or devices are comparable, leading to more reliable analyses [61].

However, it is important to discuss potential drawbacks of using ComBat and NPEB in PET radiomics. One limitation is the potential over-correction of data, which could lead to the loss of biologically meaningful variations among different patient groups or conditions. Another concern is that both techniques assume that batch effects are the primary source of variability in the dataset, which might not always be the case. This assumption can lead to misinterpretation of the data if other sources of variability are present but not accounted for.

### Statistical analysis

In the current study, we used the ‘irr’ library (version 0.84.1) for statistical analysis [45–47]. For each radiomic feature, the intraclass correlation coefficient (ICC) was determined using R version 4.0.4 (The R Foundation, Vienna, Austria) both before and after harmonization. Considering Koo and Li’s guideline [48], two-way random effects with complete agreement and multi-raters were conducted to calculate the ICC. An ICC of less than 0.5, 0.5 ≤ ICC < 0.75, 0.75 ≤ ICC < 0.9, and ICC ≥ 0.9 reflect low, rational, promising, and outstanding reliability, respectively. The Kruskal–Wallis (KW) test, a non-parametric test, was utilized to examine an independent dataset and assess differences between various segmentation methods used in this study. The KW test can be applied to distinguish whether there are significant differences in a sequential or continuous dependent variable among the independent variable of the data set (segmentations in the current study). As the goal of this study was to investigate the variability of features without any form of pre-processing, we did not normalize images or features since normalization might introduce a confounding factor for calculating the variability of radiomic features (using ICC).

### Feature selection and ML classifier

An in-house-developed ML classifier and feature selection pipeline from Scikit-Learn library was implemented in Python.

#### Random Under Sampler

To address the issue of imbalance in our datasets, a Random Under Sampler was employed [49]. This method strategically reduces the number of examples in the majority class to match the quantity in the minority class, aiming to create a balanced distribution of classes. By doing so, the likelihood of the model’s overfitting to the majority class is reduced, and its ability to learn and predict the minority class is improved. The rationale behind using an under-sampling technique is to force the model to focus on the more complex patterns that are characteristic of the minority class, thereby potentially uncovering subtle but important signals that could be overlooked in an imbalanced dataset.

#### Feature selection

For feature selection, a Linear Support Vector Classifier (LinearSVC) with L1 regularization was used. This approach regulates feature sparsity by assigning zero to coefficients of non-informative features, selecting only the most relevant features [50]. The *C* parameter in LinearSVC feature selection methods controls the trade-off between margin maximization and classification error minimization. As the *C* parameter decreases, it emphasizes regularization and feature sparsity [51]. In this study, the *C* parameter was equal to 0.05, which is considered low. The penalty parameter is set to l1 for L1 regularization, which contributes to selecting important features. The dual parameter is set to alse to accommodate L1 regularization as well. In the end, at most 10 features were selected (max_features = 10).

#### ML classifier

An SVM classifier was implemented to predict recurrence in NSCLC patients [52]. SVMs, as supervised learning methods, aim to discover an ideal hyperplane that separates various classes in a high-dimensional feature space [52] with the following parameters: *C* (regularization parameter): larger *C* values may contribute to better training set accuracy, but too large values might cause overfitting. To prevent over/underfitting biases, we used a *C* value of 1.0, which is considered moderate. Kernel type: As medical data and binary classification problem like predicting recurrence in NSCLC, which is considered a non-linear high-dimensional space, we used an RBF kernel. Class weight: Although we used a Random Under Sampler method to mitigate the impact of imbalanced dataset to fit our results better, we used imbalanced class weight in our parameters as well to maximize the model’s performance and accuracy in the presence of imbalanced data, a common challenge in medical datasets and predictive modeling.

#### Fivefold cross-validation

Fivefold nested cross-validation was performed using StratifiedKFold with ‘n_splits’ set to 5. This approach ensures the dataset is divided into balanced subsets for training, evaluation, and validation on unforeseen datasets. Nested cross-validation required two cross-validation steps: an outer loop and an inner loop [53]. Outer loop: For model assessment, the data were divided into fivefold. While the model was trained on the remaining data, each fold is utilized as a hold-out validation set. Inner loop: The data were further subdivided into training and validation subsets inside each fold of the outer loop. This inner loop was utilized to fine-tune the hyperparameters and choose the optimum model configuration. The results provided in this study are for the outer loop of the nested cross-validation, which was chosen for evaluating the model’s performance on different validation sets [53].

## Results

**Fig. 3:**
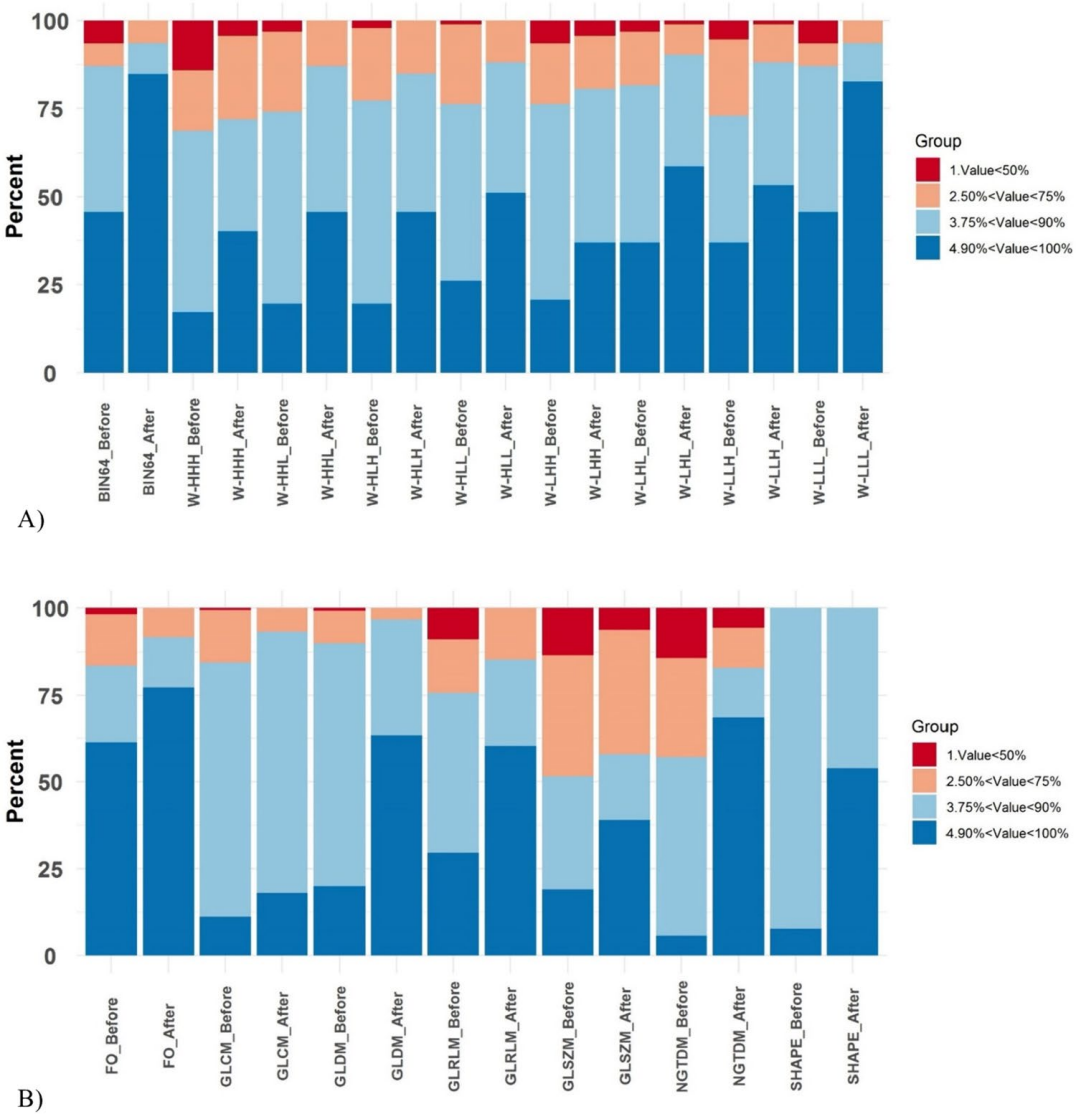
ICC percentage bar plot of the different radiomic feature sets depicting different radiomic features’ family extracted in this study **A** before and **B** after NPEB ComBat harmonization. The ICC values were classified into 4 categories: ICC < 50% (dark red), 50% < ICC < 75% (light red), 75% < ICC < 90% (light blue), ICC > 90% (dark blue). The feature sets include 64 fixed bin widths and wavelets with multiple wavelet decompositions (LHH, HLL, HHL, LLL, HHH, HLH, LHL, and LLH) without any filter. Various image segmentation techniques were applied to PET images, including K-means, Watershed, FCM, IT (40, 45, and 50% thresholds), RG, LAC, and manual contouring

Figure 3A shows a bar plot representing the ICC values of the different feature sets extracted in this study through various image segmentation techniques, including K-means, watershed, FCM, IT (40, 45, and 50 percent), RG, LAC, and manual contouring, applied to PET images. In this study, all analyses were performed twice, i.e., with and without applying the NPEB ComBat harmonization. Feature sets include 64 fixed bin widths (without any filter) and wavelets with various wavelet decompositions (LHH, HLL, HHL, LLL, HHH, HLH, LHL, and LLH). In this figure, the ICC value is categorized into four groups; ICC < 50% (low robustness, dark red), 50% ≤ CC < 75% (medium robustness, light red), 75% ≤ CC < 90% (qualified robustness, light blue), ICC ≥ 90% (high robustness, dark blue). Figure 3A highlights the influence of image segmentation variability on radiomic features after harmonization.

Figure 3B is a bar plot indicating the percentage of ICC radiomic features family extracted in this study, including shape, FO, GLDM, GLRLM, and GLSZM, after applying different PET image segmentation methods. The ICC percentage with and without NPEB ComBat harmonization depicts the effect of harmonization on radiomic features’ repeatability. Figure 3B illustrates the percentage of the four examined belonging to diverse radiomic families. The beneficial impact of ComBat harmonization is clear where the number of robust features surged after using the harmonization algorithm. It is evident from Fig. 3B that first-order statistic features showed the best performance with 61 and 77% robust features before and after ComBat harmonization, followed by GLRLM and GLDM. Although NGTDM (5%), shape (7%), and GLCM (11%) feature families showed the least robustness before ComBat harmonization, NGTDM (68%) and shape (53%) feature families surged in the number of repeatable features, whereas GLCM (20%) did not respond to ComBat harmonization.

Supplemental Fig. 1 shows a box plot reflecting a regulated method of visualizing statistical parameters distribution. This plot illustrates the ICC values of various features set in the present study, after (upper panel) and before (lower panel) applying NPEB ComBat harmonization. This is a box plot showing the range of ICC values concentration and distribution between 0 and 1, which belongs to various feature sets extracted before and after ComBat harmonization. It is apparent that after ComBat harmonization, the box plot of all feature sets focused (area within the first quartile (Q1/25th Percentile) and third quartile (Q3/75th Percentile) narrowed) on the higher (ICC) value. Besides, ICC value distribution after ComBat harmonization shrank. Wavelet HHH and LHH’s ICC value distribution was more than the other feature sets.

**Fig. 4:**
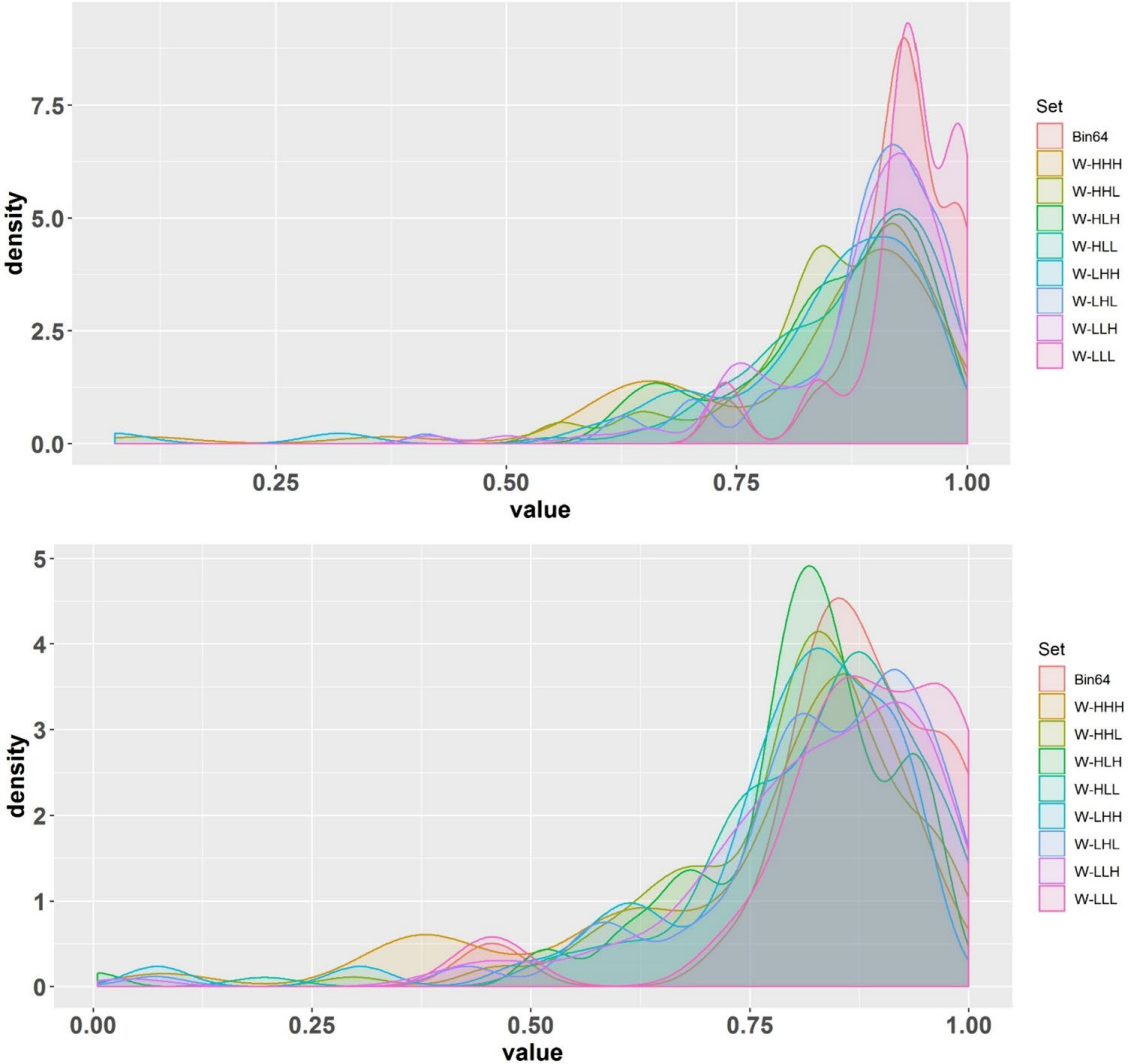
Probability density distribution (PDD) of ICC values before (lower panel) and after (upper panel) applying NPEB ComBat harmonization on the radiomic features for the different feature sets including 64 fixed bin widths and wavelets with multiple wavelet decompositions (LHH, HLL, HHL, LLL, HHH, HLH, LHL, and LLH), without any filter. The higher the focus of a graph’s density toward larger values (close to 1), the more robust the feature is to the effective factor

Figure 4 is a probability density distribution (PDD) of ICC values, i.e., the distribution of ICC values belonging to radiomic features set when applying diverse segmentation and harmonization methods. The density of each chart in the PDD figure depicts the probability of features’ robustness against various segmentations. Consequently, the further a density concentration of a graph moves to the right (close to 1), the more likely the feature set is robust. The upper image represents the PDD of the ICC values after applying NPEB ComBat harmonization to the radiomic features. The PDD of the ICC values before applying NPEB ComBat harmonization is shown in the lower panel of Fig. 4. It can be seen that implementing harmonization will push the ICC values to the right (equal to 1) and narrow the density distribution.

**Fig. 5:**
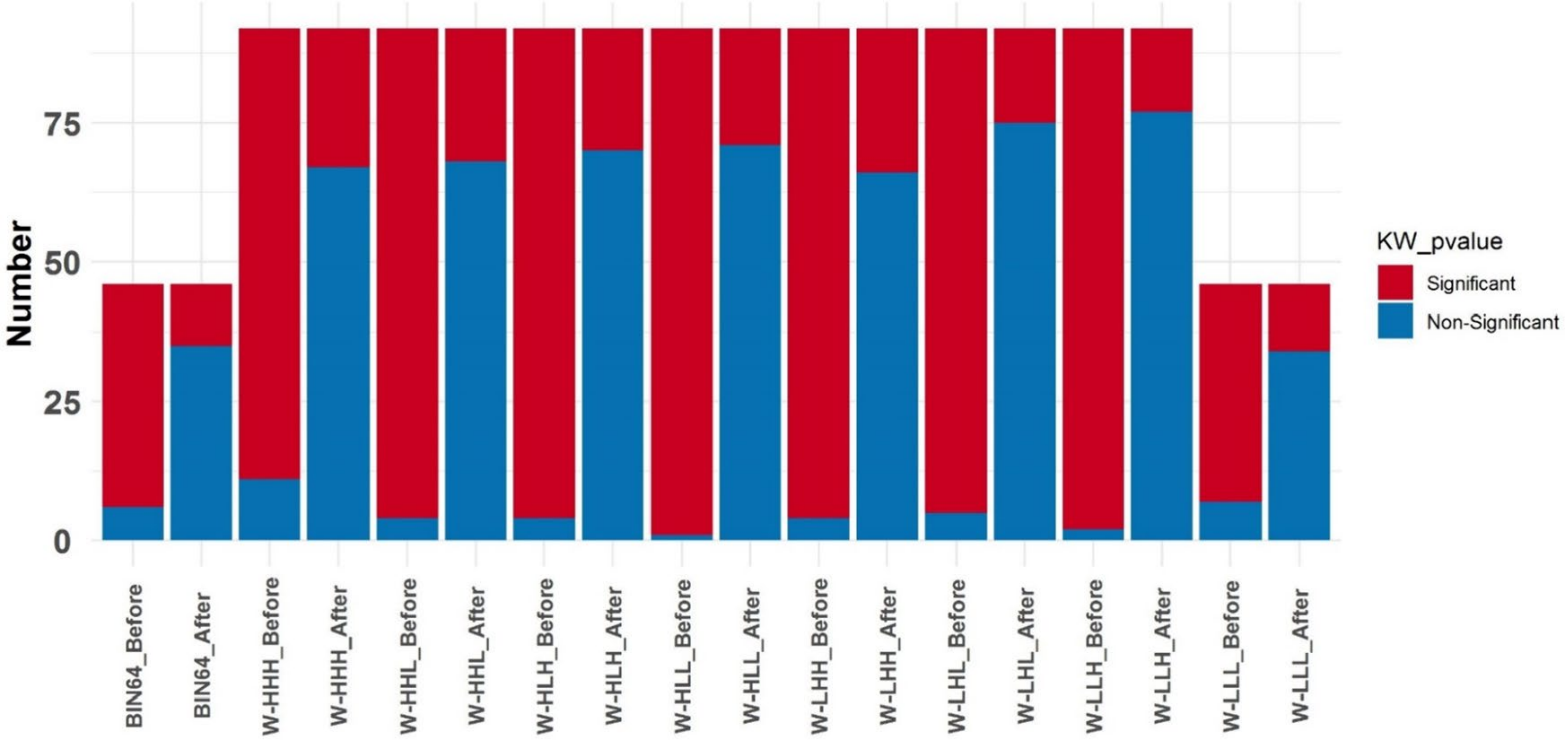
Kruskal–Wallis (KW) p value bar plot of the extracted features before and after NPEB ComBat harmonization. The various feature sets include 64 fixed bin widths and wavelets with multiple wavelet decompositions (LHH, HLL, HHL, LLL, HHH, HLH, LHL, and LLH) without any filter. Each feature set’s number of significant (red) and non-significant (blue) features is depicted

The results of the (KW) p value tests through a number of significant (red) and non-significant (blue) radiomic features belonging to each feature set before and after NPEB ComBat harmonization are depicted in Fig. 5. The red color indicates those features with statistically significant differences. According to the results of the KW test, wavelet-based radiomic features had much more significant radiomic features than the original feature set before ComBat harmonization. However, after ComBat harmonization, the number of non-significant radiomic features increased considerably, whereas wavelet LLH-based features had the highest number of non-significant radiomic features after ComBat harmonization.

Table 1 depicts the top 20 robust radiomic features against segmentation variability. The radiomic features are arranged in descending order based on the ICC, before and after NPEB ComBat harmonization. First-order (FO) statistic features were not considered in the present table, as most FO features have an ICC ≥ 0.95 (extremely repeatable) against segmentation. Complete information about feature variability is reported in Supplemental Table 2 where the radiomic features are arranged from the highest to lowest ICC.

**Table 1:** Top 20 high performing features in terms of robustness against the diverse segmentation methods, after (upper part) and before (lower part) applying NPEB ComBat harmonization

Radiomic features after ComBat harmonization	ICC value
wavelet-LLH _ glrlm _ Long Run Low Gray Level Emphasis	0.978
Original _ glszm _ Large Area Emphasis	0.978
Original _ glszm _ Large Area Low Gray Level Emphasis	0.978
Original _ glszm _ Large Area High Gray Level Emphasis	0.978
wavelet-LLL _ glszm _ Large Area Emphasis	0.978
wavelet-LLL _ glszm _ Large Area Low Gray Level Emphasis	0.978
wavelet-LLL _ glszm _ Large Area High Gray Level Emphasis	0.978
wavelet-LLH _ glrlm _ Run Variance	0.977
wavelet-LHL _ glrlm _ Long Run Low Gray Level Emphasis	0.974
wavelet-LLH _ glrlm _ Long Run Emphasis	0.973
wavelet-LHL _ gldm _ Dependence Non-Uniformity Normalized	0.969
wavelet-LHL _ glrlm _ Run Variance	0.967
wavelet-HLL _ glszm _ Large Area Low Gray Level Emphasis	0.967
wavelet-LHL _ glrlm _ Long Run Emphasis	0.964
wavelet-HLL _ glszm _ Zone Variance	0.964
wavelet-LHL _ gldm _ Large Dependence Emphasis	0.964
wavelet-LHL _ glrlm _ Run Percentage	0.962
wavelet-HLL _ glszm _ Large Area Emphasis	0.960
wavelet-LHL _ gldm _ Dependence Entropy	0.958
wavelet-HHH _ glrlm _ Long Run High Gray Level Emphasis	0.956

Radiomic features before ComBat harmonization	ICC value
wavelet-LLH_ glrlm _ Run Variance	0.967
wavelet-LHL _ glrlm _ Long Run Low Gray Level Emphasis	0.964
wavelet-LLH _ glrlm _ Long Run Emphasis	0.962
wavelet-LHL _ gldm _ Dependence Non-Uniformity Normalized	0.960
Original _ glszm _ Large Area Emphasis	0.956
Original _ glszm _ Large Area Low Gray Level Emphasis	0.956
Original _ glszm _ Large Area High Gray Level Emphasis	0.956
wavelet-LLL _ glszm _ Large Area Emphasis	0.956
wavelet-LLL _ glszm _ Large Area Low Gray Level Emphasis	0.956
wavelet-LLL _ glszm _ Large Area High Gray Level Emphasis	0.956
wavelet-HLL _ glszm _ Large Area Low Gray Level Emphasis	0.955
wavelet- LHL _ glrlm _ Run Variance	0.952
wavelet-HLL _ glszm _ Zone Variance	0.951
wavelet-HHH _ gldm _ Dependence Non-Uniformity Normalized	0.948
wavelet-LHL _ glrlm _ Long Run Emphasis	0.948
wavelet-LHL _ gldm _ Dependence Entropy	0.948
wavelet-HLL _ glszm _ Large Area Emphasis	0.947
wavelet-LLH _ gldm _ Dependence Non-Uniformity Normalized	0.946
wavelet-HHH _ gldm _ Dependence Entropy	0.944
wavelet-LHH _ gldm _ Dependence Non-Uniformity Normalized	0.943
wavelet-LLH _ glrlm _ Run Variance	0.967

First-order features were eliminated (most FO features were highly repeatable (ICC ≥ 0.95) against segmentation)

**Fig. 6:**
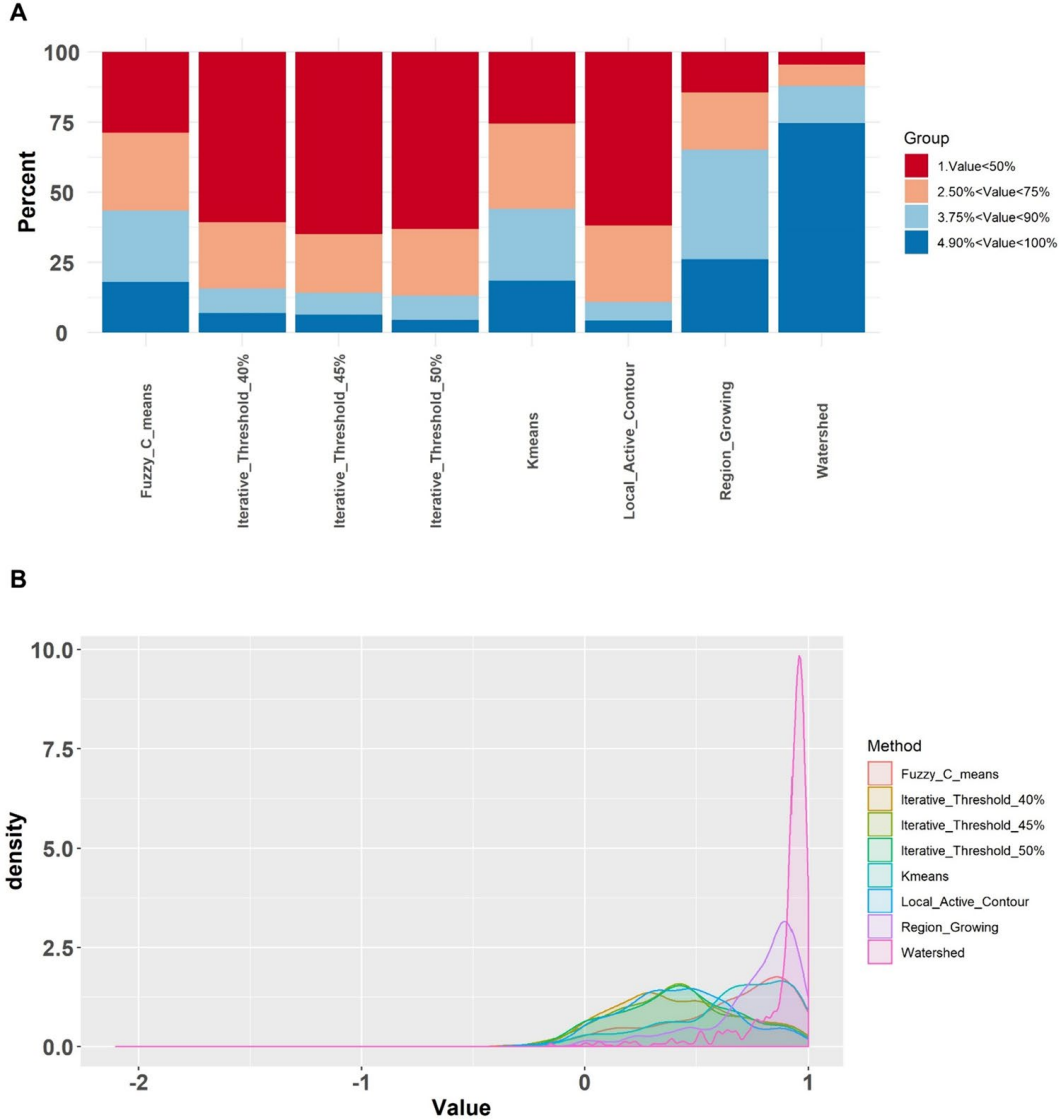
Bar plot (**A**) and PDD (**B**) of the results of the ICC metric for radiomic features between manual contouring and other semi-automated and automated PET image segmentation techniques implemented in the current study

Figure 6A represents the ICC bar plot of various radiomic features between manual contouring and other semi-automated and automated segmentations examined in this study. It is evident that the watershed showed the highest reproducibility with 74% robust features, followed by region-growing and K-means with 26 and 18% robust features, respectively. Local active contour had the lowest number of robust features (4%). In Fig. 6B, we indicate the PDD of the ICC between manual contouring and other semi-automated and automated segmentation techniques examined in this study. The concentration of watershed segmentation near 1 indicates the robustness of the radiomic features extracted from this contour. A non-parametric KW test was also conducted to examine the impact of various segmentation methods on radiomic features (Supplemental Fig. 2). Moreover, the heatmap of the ICC results before and after NPEB ComBat harmonization is plotted in Supplemental Fig. 3.

The impact of harmonization on the reliability of radiomic features is remarkable, and the percentage of the fourth group (ICC > 90%) was raised in all feature sets. The original features set (64 fixed bin widths without any filter) and wavelet (LLL) set showed the highest repeatability before ComBat harmonization. After ComBat harmonization, these two feature sets showed dramatic growth in terms of the percentage of highly reliable (ICC > 90%) features to more than 70%, and there was no feature with an ICC less than 50%. The third place of robustness against segmentation variation goes to wavelet (LHL) with a 58% robust feature (out of 92).

Table 2 summarizes the performance of the different segmentation techniques in predicting recurrence in patients with NSCLC before and after ComBat harmonization. Before ComBat harmonization, manual contouring showed the highest performance (accuracy: 0.850, AUC: 0.850, sensitivity: 1, specificity: 0.700), followed by K-means (accuracy: 0.825, AUC: 0.825, sensitivity: 0.700, specificity: 0.950). After ComBat harmonization, watershed with a big jump showed the highest performance (accuracy: 0.875, AUC: 0.875, sensitivity: 0.800, specificity: 0.950) followed by manual contouring with similar results as before ComBat (accuracy: 0.850, AUC: 0.850, sensitivity: 1, specificity: 0.700) and iterative threshold 50% (accuracy: 0.825, AUC: 0.825, sensitivity: 1, specificity: 0.750). As is evident from Table 2, although downward trend might happen after ComBat harmonization, which is dependent on the segmentation method, some of the results improved after ComBat harmonization, especially sensitivity and specificity, which show improvement in their ability to correctly identify positive cases (sensitivity) and negative cases (specificity).

**Table 2:**
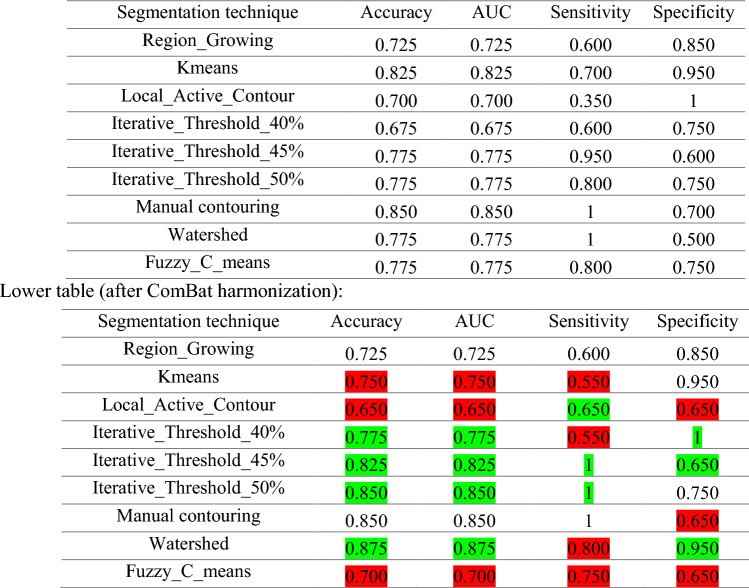
Results of LinearSVC feature selection technique and SVM machine learning classifier of different PET segmentation techniques before (upper table) and after (lower table) ComBat harmonization with improved values are highlighted in green while decreased values are highlighted in red

## Discussion

A large number of studies (over 2600 articles/year in 2022 according to PubMed) have shown the great potential of radiomics analysis in clinical diagnosis, prognosis, and outcome prediction. However, several factors might affect radiomic features’ robustness, including the segmentation method, which impacts radiomic features to a large extent. While all segmentation algorithms have a unique goal, contouring the ROI, slight changes in the defined contours significantly impact the extracted radiomic features.

In the current study, 120 patient images were used to assess 9 segmentation methods belonging to 3 categories, namely automated, semi-automated, and manual contouring applied to PET images. The repeatability of radiomic features has always been an issue in radiomic studies. A number of factors might affect radiomic features individually and collectively based on data acquisition, image reconstruction, and image processing scenarios. Altogether, these factors have a significant impact on radiomic features. Various strategies have been proposed to tackle this challenge, including but not limited to selecting robust features or using a harmonization method to reduce the variability of the features. In this work, the impact of segmentation methods was explored. We attempted to eliminate or minimize other factors. For instance, all images were reconstructed using the same reconstruction algorithm. At the same time, the NPEB method was implemented to harmonize the radiomic features and decrease the impact of factors affecting their robustness (segmentation). To further analyze the impact of ComBat harmonization on classifier metrics, a ML pipeline was implemented on the dataset before and after ComBat harmonization.

It is evident from Fig. 1 that the LAC often results in smaller shapes compared to manual segmentation. This might indicate that LAC is not capturing all the relevant ROI. The other methods, although automated, seem to produce shapes that are closer in size to the manual technique, which suggests they might be more reliable for replicating expert-level segmentation. The ‘Watershed’ method, in particular, shows a unique pattern, differing slightly in shape from the manual method.

A number of strategies were proposed to overcome the poor reliability of radiomic features; selecting robust features against the different factors influencing their relevance is the principal one. Another solution is the use of a harmonization method, e.g., ComBat. These two solutions were explored in this study. Altogether, 749 radiomic features were extracted from each segmented region. To quantify the magnitude of variability of each radiomics feature, the ICC was measured for each one. All analyses were performed twice before and after ComBat harmonization. Radiomic features with an ICC of more than 0.95 were considered robust with excellent repeatability. Even though contouring variability significantly impacts radiomic features, few studies have addressed this issue [27, 30, 54].

Yang et al. [27] examined the effect of manual segmentation on PET radiomic features in patients with lung cancer. In their study, ten radiation oncologists segmented PET images to extract 25 texture features from each ROI. Gray-Level Neighborhood Difference Matrix (GLNDM) features showed the highest performance in terms of reliability, whereas GLRLM and GLSZM radiomic family showed the highest robustness. The difference might be explained by the fact that in our study, we examined fully automated and semi-automated segmentations in addition to manual segmentation. In another study, Bashir et al. [55] studied the impact of multiple image segmentation algorithms, including fuzzy locally adaptive Bayesian (FLAB), fixed thresholding (40%), and manual delineation (performed by three physicians) on PET radiomic features in 53 patients with NSCLC. They reported that 40% fixed thresholding and manual contouring yielded the smallest and largest volume measurements. Moreover, 62, 27, and 20 radiomic features showed ICC > 0.85 for fixed thresholding, FLAB, and manual contouring, respectively. Leijenaar et al. [56] examined the repeatability of radiomic features against inter-observer variability of manual segmentation and test–retest in PET/CT images of 27 NSCLC patients. Five physicians manually segmented PET images to extract 98 radiomic features from each ROI. The results of their study demonstrated that GLCM texture features achieved the best performance in terms of reliability. However, compared to our study, GLCM-based radiomic features did not show a good performance in terms of reliability, since only 11% and 18% among them showed excellent reliability (ICC > 0.9). In a CT radiomics study, Pavic et al. [30] investigated the impact of contouring variability on an NSCLC dataset. They reported that 63 out of the 105 extracted radiomic features were robust against delineation variability.

It was not unexpected to observe that the LLL wavelet response maps generate substantially more stable features compared to other response maps. The LLL wavelet essentially provides a low-pass filtered rendition of the original image, thereby eliminating noise and other abrupt transitions. This results in a cleaner, more coherent image that inherently lends itself to the extraction of robust and consistent features.

The application of ComBat harmonization to enhance the reproducibility of radiomic features in multi-center studies has been examined in a number of previous studies [57–60]. Furthermore, Leithner et al. [61] examined the impact of ComBat on tissue classification using PET radiomics in a PET/MRI study, where they reported that ComBat is useful in multi-center studies. Shiri et al. [62] demonstrated that ComBat harmonization improved gene mutation status prediction in non-small cell lung cancer. Another study examined the impact of feature harmonization on radio-genomics analysis for the prediction of KRAS and EGFR mutations from non-small cell lung cancer from PET/CT images [63]. It was reported that ComBat harmonization had a significant impact on the prediction. Despite the fact that radiomic features are potent instruments for prognosis, diagnosis, and outcome prediction, their reproducibility and repeatability have constantly been scrutinized owing to their sensitivity to several factors. Our results are in agreement with previous findings who reported that implementing ComBat harmonization might have a significant impact on the repeatability and reproducibility of radiomic features not only in multi-center studies but also in a single-site study where other factors, i.e., segmentation methods, may affect radiomic features.

Manual contouring produced the best results before ComBat harmonization (accuracy: 0.850, AUC: 0.850, sensitivity: 1, specificity: 0.700), followed by K-means (accuracy: 0.825, AUC: 0.825, sensitivity: 0.700, specificity: 0.950). After ComBat harmonization, watershed showed the most performance improvement, achieving the highest scores (accuracy: 0.875, AUC: 0.875, sensitivity: 0.800, specificity: 0.950). This was followed by manual contouring, which retained its previous results before ComBat harmonization (accuracy: 0.850, AUC: 0.850, sensitivity: 1, specificity: 0.700), and then by iterative threshold 50%, which also showed a good jump in terms of outcome (accuracy: 0.825, AUC: 0.825, sensitivity: 1, specificity: 0.750). These results demonstrated that implementing ComBat harmonization might be very helpful in terms of ML outcome improvement, especially for watershed segmentation which had the lowest number of robust features compared to manual contouring. After ComBat harmonization, sensitivity and specificity were dramatically improved in most cases or remained stable in manual contouring. Boosting the sensitivity is relatively important as missing positive cases could have serious consequences, such as in clinical diagnosis where a disease needs to be well characterized to provide timely treatment [64]. In addition, boosting specificity is essential as it may minimize the false positives rate [65]. The enhancement in sensitivity and specificity could be attributed to either the individual or combined influence of several factors: standardization of features, minimization of bias, and the improvement in data quality following the application of ComBat harmonization [66].

Although a number of studies examined the impact of ComBat harmonization on the reproducibility of radiomic features [67, 68], focus on the impact of harmonization on ML outcomes is lacking. However, our results in terms of ML outcome are consistent with previous studies substantiating the fact that ComBat harmonization enhances the machine learning classifiers performance [28].

This study inherently bears a number of limitations. First, the sample size was relatively small. Second, fully automated PET image segmentation algorithms (e.g., those using deep learning techniques) reported in more recent studies should also be considered [69, 70]. Third, exploring the repeatability and reproducibility of radiomic features using a phantom study is also needed to double-check these results in a controlled experiment. The application of robust features in a deep learning clinical study and the level of robustness of selected features by feature selection algorithms were not considered in the current study and will be evaluated in future studies. Finally, future studies will explore and validate the harmonization process in a multi-center, multi-scanner scenario to ensure the generalizability of our conclusions and the effectiveness of ComBat harmonization across diverse imaging platforms and settings as our study used data from single PET scanner.

## Conclusion

The remarkable impact of segmentation variability on PET radiomic features may come up with errors in quantitative analysis. In this work, we proposed a solution to mitigate the adverse effects of this variability. ComBat harmonization enhances the robustness of radiomic features across all families and increases the number of robust features. In addition, harmonization improves machine learning performance, particularly for datasets with fewer robust features. Selecting features robust to diverse segmentation techniques is crucial for reducing errors in radiomic studies and enhancing accuracy. Reasonable performance was achieved by choosing robust features and applying ComBat harmonization.

### Supplementary Information

Below is the link to the electronic supplementary material.

Supplementary file1 (PDF 2125 KB)

## Data Availability

The authors will provide access to the data and data processing pipeline supporting the findings of this study upon reasonable request.
