# Supplementary material for: The effect of harmonization on the variability of PET radiomic features extracted using various segmentation methods
Source: Ann Nucl Med. 2024 Apr 4;38(7):493–507. doi: 10.1007/s12149-024-01923-7 (PMC11217131; doi:10.1007/s12149-024-01923-7)
Supplement: Supplementary file 1 — Supplementary file1 (PDF 2125 KB) [file 12149_2024_1923_MOESM1_ESM.pdf]

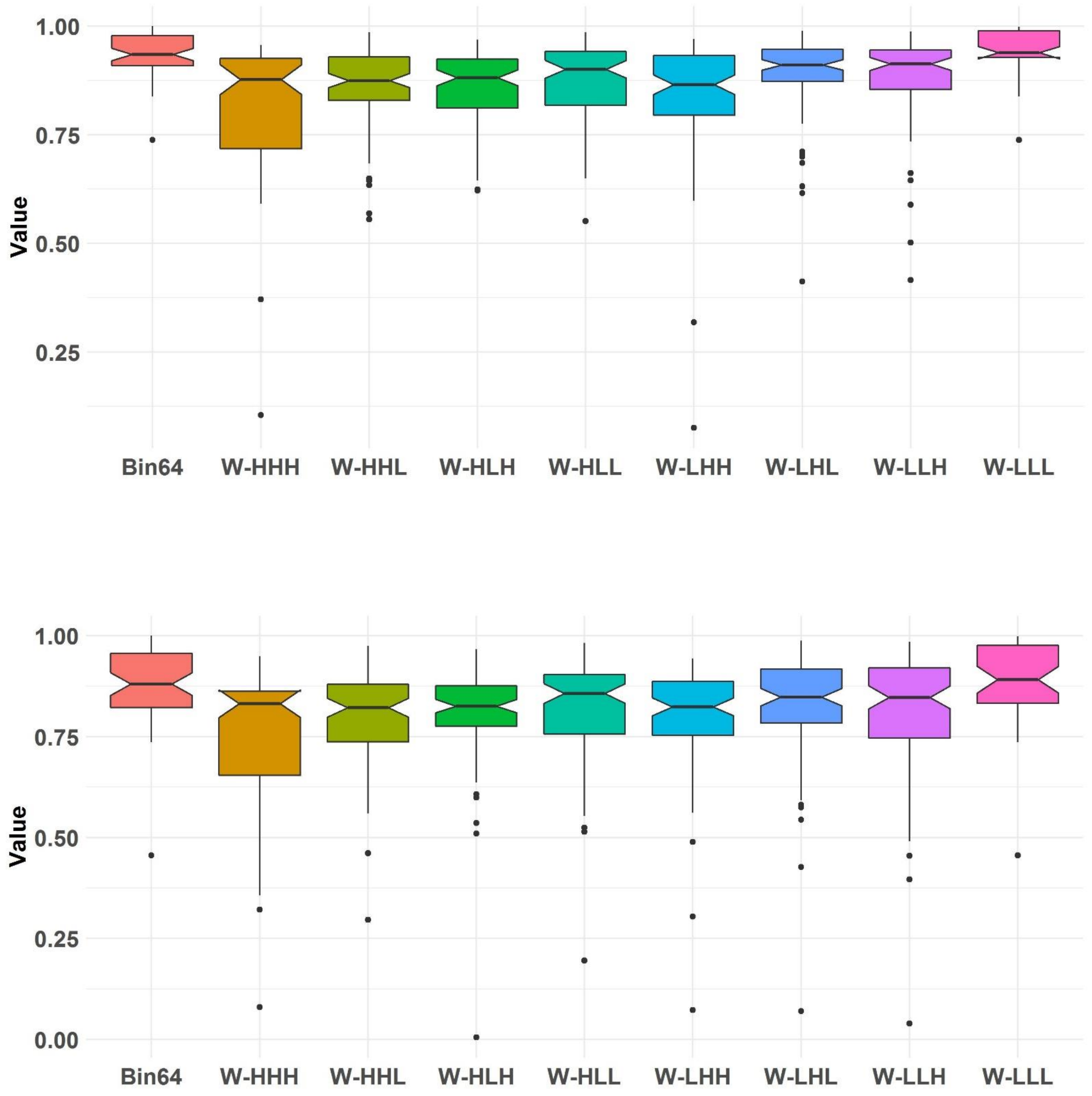

Supplementary Figure 1. Box plots of ICC values belonging to different feature sets, after (upper panel) and before (lower panel), applying NPEB harmonization on the radiomic features. The feature sets include 64 fixed bin widths and wavelets with multiple decompositions (LHH, HLL, HHL, LLL, HHH, HLH, LHL, and LLH) without any filter. Various PET image segmentation techniques were applied, including KM, watershed, FCM, IT (40, 45, and 50 % thresholds), RG, LAC, and manual contouring. The ICC values are between 0 and 1 (0= not reliable and 1= highly reliable).

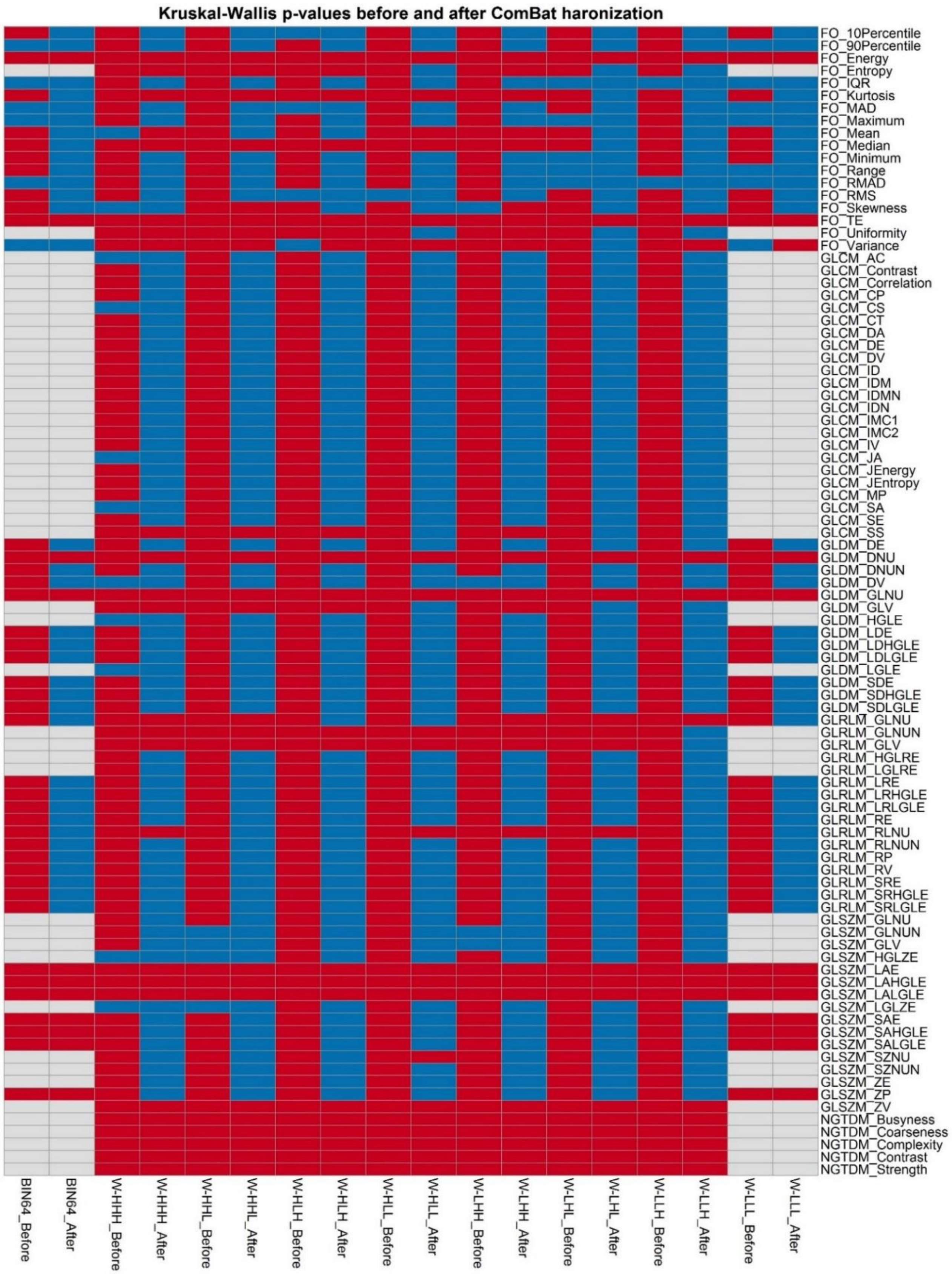

Supplementary Figure 2. Heatmap of the KW test results before and after Combat NPEB harmonization. Significant features based on the KW test are displayed in red, whereas non-significant features are shown in blue.

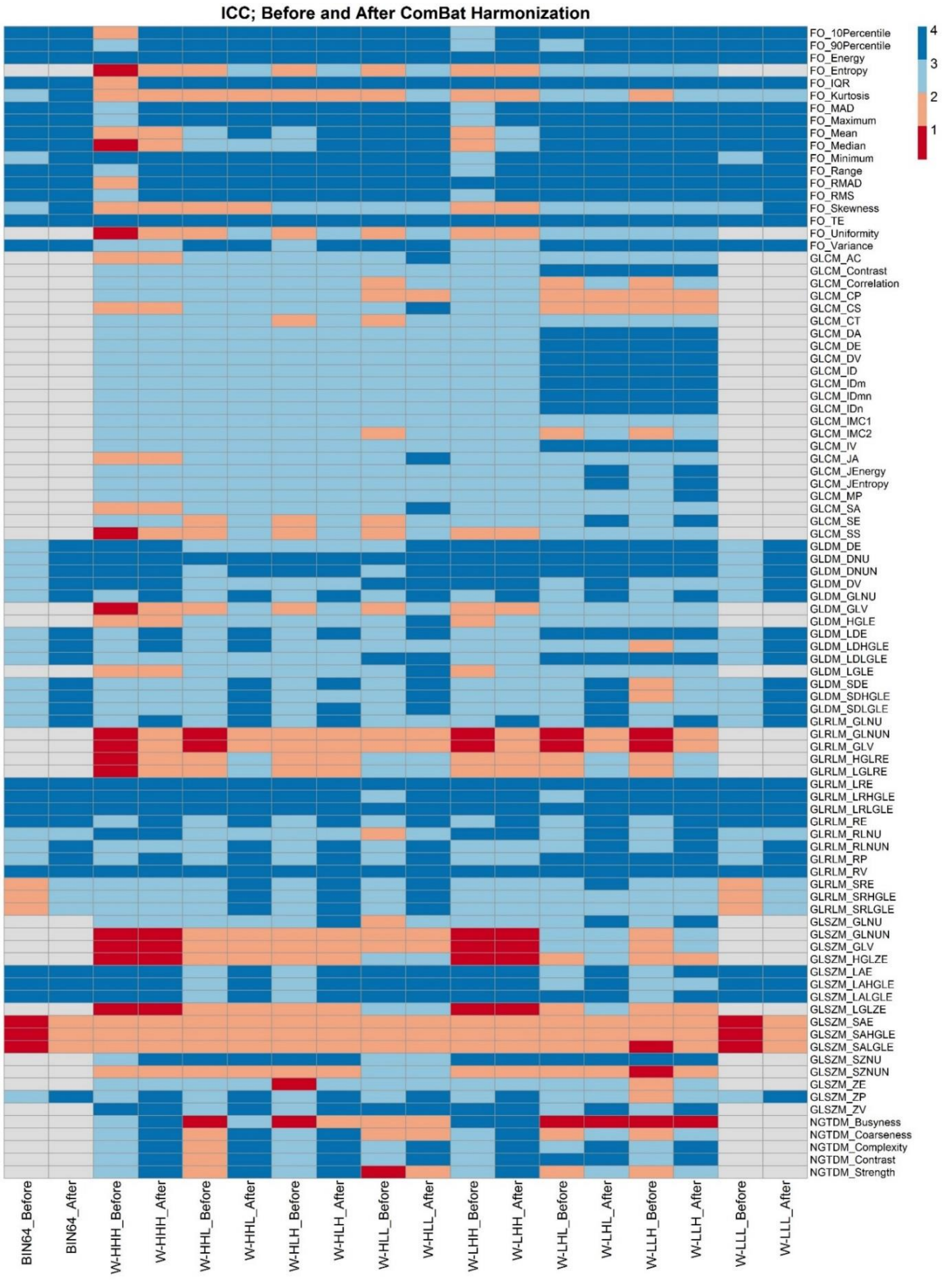

Supplementary Figure 3. Heatmap of the ICC results before and after Combat NPEB harmonization. The ICC values belonging to all original radiomic features (64 fixed bin widths and without any filter) were categorized into 4 groups, i.e., ICC<50% (dark red), 50%<ICC<75% (pale red), 75%<ICC<90% (pale blue), ICC>90% (dark blue), before and after ComBat harmonization.

Supplementary Table 1. Radiomics features' name, set, and family extracted in this study.

Set	Type	Name
**Original**	shape	Maximum3DDiameter
**Original**	shape	Maximum2DDiameterSlice
**Original**	shape	Sphericity
**Original**	shape	MinorAxis
**Original**	shape	Elongation
**Original**	shape	SurfaceVolumeRatio
**Original**	shape	Volume
**Original**	shape	MajorAxis
**Original**	shape	SurfaceArea
**Original**	shape	Flatness
**Original**	shape	LeastAxis
**Original**	shape	Maximum2DDiameterColumn
**Original**	shape	Maximum2DDiameterRow
**Original**	gldm	DependenceEntropy
**Original**	gldm	DependenceNonUniformity
**Original**	gldm	GrayLevelNonUniformity
**Original**	gldm	SmallDependenceEmphasis
**Original**	gldm	SmallDependenceHighGrayLevelEmphasis
**Original**	gldm	DependenceNonUniformityNormalized
**Original**	gldm	LargeDependenceEmphasis
**Original**	gldm	LargeDependenceLowGrayLevelEmphasis
**Original**	gldm	DependenceVariance
**Original**	gldm	LargeDependenceHighGrayLevelEmphasis
**Original**	gldm	SmallDependenceLowGrayLevelEmphasis
**Original**	firstorder	InterquartileRange
**Original**	firstorder	Skewness
**Original**	firstorder	Median
**Original**	firstorder	Energy
**Original**	firstorder	RobustMeanAbsoluteDeviation
**Original**	firstorder	MeanAbsoluteDeviation
**Original**	firstorder	TotalEnergy
**Original**	firstorder	Maximum
**Original**	firstorder	RootMeanSquared
**Original**	firstorder	90Percentile
**Original**	firstorder	Minimum
**Original**	firstorder	Range
**Original**	firstorder	Variance
**Original**	firstorder	10Percentile
**Original**	firstorder	Kurtosis
**Original**	firstorder	Mean
**Original**	glrlm	ShortRunLowGrayLevelEmphasis
**Original**	glrlm	RunVariance
**Original**	glrlm	GrayLevelNonUniformity

**Original**	glrlm	LongRunEmphasis
**Original**	glrlm	ShortRunHighGrayLevelEmphasis
**Original**	glrlm	RunLengthNonUniformity
**Original**	glrlm	ShortRunEmphasis
**Original**	glrlm	LongRunHighGrayLevelEmphasis
**Original**	glrlm	RunPercentage
**Original**	glrlm	LongRunLowGrayLevelEmphasis
**Original**	glrlm	RunEntropy
**Original**	glrlm	RunLengthNonUniformityNormalized
**Original**	glszm	LargeAreaEmphasis
**Original**	glszm	SmallAreaHighGrayLevelEmphasis
**Original**	glszm	ZonePercentage
**Original**	glszm	LargeAreaLowGrayLevelEmphasis
**Original**	glszm	LargeAreaHighGrayLevelEmphasis
**Original**	glszm	SmallAreaEmphasis
**Original**	glszm	SmallAreaLowGrayLevelEmphasis
**w-HHH**	gldm	GrayLevelVariance
**w-HHH**	gldm	HighGrayLevelEmphasis
**w-HHH**	gldm	DependenceEntropy
**w-HHH**	gldm	DependenceNonUniformity
**w-HHH**	gldm	GrayLevelNonUniformity
**w-HHH**	gldm	SmallDependenceEmphasis
**w-HHH**	gldm	SmallDependenceHighGrayLevelEmphasis
**w-HHH**	gldm	DependenceNonUniformityNormalized
**w-HHH**	gldm	LargeDependenceEmphasis
**w-HHH**	gldm	LargeDependenceLowGrayLevelEmphasis
**w-HHH**	gldm	DependenceVariance
**w-HHH**	gldm	LargeDependenceHighGrayLevelEmphasis
**w-HHH**	gldm	SmallDependenceLowGrayLevelEmphasis
**w-HHH**	gldm	LowGrayLevelEmphasis
**w-HHH**	glcm	JointAverage
**w-HHH**	glcm	SumAverage
**w-HHH**	glcm	JointEntropy
**w-HHH**	glcm	ClusterShade
**w-HHH**	glcm	MaximumProbability
**w-HHH**	glcm	Idmn
**w-HHH**	glcm	JointEnergy
**w-HHH**	glcm	Contrast
**w-HHH**	glcm	DifferenceEntropy
**w-HHH**	glcm	InverseVariance
**w-HHH**	glcm	DifferenceVariance
**w-HHH**	glcm	Idn
**w-HHH**	glcm	Idm
**w-HHH**	glcm	Correlation
**w-HHH**	glcm	Autocorrelation
**w-HHH**	glcm	SumEntropy

**w-HHH**	glcm	SumSquares
**w-HHH**	glcm	ClusterProminence
**w-HHH**	glcm	Imc2
**w-HHH**	glcm	Imc1
**w-HHH**	glcm	DifferenceAverage
**w-HHH**	glcm	Id
**w-HHH**	glcm	ClusterTendency
**w-HHH**	firstorder	InterquartileRange
**w-HHH**	firstorder	Skewness
**w-HHH**	firstorder	Uniformity
**w-HHH**	firstorder	Median
**w-HHH**	firstorder	Energy
**w-HHH**	firstorder	RobustMeanAbsoluteDeviation
**w-HHH**	firstorder	MeanAbsoluteDeviation
**w-HHH**	firstorder	TotalEnergy
**w-HHH**	firstorder	Maximum
**w-HHH**	firstorder	RootMeanSquared
**w-HHH**	firstorder	90Percentile
**w-HHH**	firstorder	Minimum
**w-HHH**	firstorder	Entropy
**w-HHH**	firstorder	Range
**w-HHH**	firstorder	Variance
**w-HHH**	firstorder	10Percentile
**w-HHH**	firstorder	Kurtosis
**w-HHH**	firstorder	Mean
**w-HHH**	glrlm	ShortRunLowGrayLevelEmphasis
**w-HHH**	glrlm	GrayLevelVariance
**w-HHH**	glrlm	LowGrayLevelRunEmphasis
**w-HHH**	glrlm	GrayLevelNonUniformityNormalized
**w-HHH**	glrlm	RunVariance
**w-HHH**	glrlm	GrayLevelNonUniformity
**w-HHH**	glrlm	LongRunEmphasis
**w-HHH**	glrlm	ShortRunHighGrayLevelEmphasis
**w-HHH**	glrlm	RunLengthNonUniformity
**w-HHH**	glrlm	ShortRunEmphasis
**w-HHH**	glrlm	LongRunHighGrayLevelEmphasis
**w-HHH**	glrlm	RunPercentage
**w-HHH**	glrlm	LongRunLowGrayLevelEmphasis
**w-HHH**	glrlm	RunEntropy
**w-HHH**	glrlm	HighGrayLevelRunEmphasis
**w-HHH**	glrlm	RunLengthNonUniformityNormalized
**w-HHH**	glszm	GrayLevelVariance
**w-HHH**	glszm	ZoneVariance
**w-HHH**	glszm	GrayLevelNonUniformityNormalized
**w-HHH**	glszm	SizeZoneNonUniformityNormalized
**w-HHH**	glszm	SizeZoneNonUniformity

**w-HHH**	glszm	GrayLevelNonUniformity
**w-HHH**	glszm	LargeAreaEmphasis
**w-HHH**	glszm	SmallAreaHighGrayLevelEmphasis
**w-HHH**	glszm	ZonePercentage
**w-HHH**	glszm	LargeAreaLowGrayLevelEmphasis
**w-HHH**	glszm	LargeAreaHighGrayLevelEmphasis
**w-HHH**	glszm	HighGrayLevelZoneEmphasis
**w-HHH**	glszm	SmallAreaEmphasis
**w-HHH**	glszm	LowGrayLevelZoneEmphasis
**w-HHH**	glszm	ZoneEntropy
**w-HHH**	glszm	SmallAreaLowGrayLevelEmphasis
**w-HHH**	ngtdm	Coarseness
**w-HHH**	ngtdm	Complexity
**w-HHH**	ngtdm	Strength
**w-HHH**	ngtdm	Contrast
**w-HHH**	ngtdm	Busyness
**w-HLL**	gldm	GrayLevelVariance
**w-HLL**	gldm	HighGrayLevelEmphasis
**w-HLL**	gldm	DependenceEntropy
**w-HLL**	gldm	DependenceNonUniformity
**w-HLL**	gldm	GrayLevelNonUniformity
**w-HLL**	gldm	SmallDependenceEmphasis
**w-HLL**	gldm	SmallDependenceHighGrayLevelEmphasis
**w-HLL**	gldm	DependenceNonUniformityNormalized
**w-HLL**	gldm	LargeDependenceEmphasis
**w-HLL**	gldm	LargeDependenceLowGrayLevelEmphasis
**w-HLL**	gldm	DependenceVariance
**w-HLL**	gldm	LargeDependenceHighGrayLevelEmphasis
**w-HLL**	gldm	SmallDependenceLowGrayLevelEmphasis
**w-HLL**	gldm	LowGrayLevelEmphasis
**w-HLL**	glcm	JointAverage
**w-HLL**	glcm	SumAverage
**w-HLL**	glcm	JointEntropy
**w-HLL**	glcm	ClusterShade
**w-HLL**	glcm	MaximumProbability
**w-HLL**	glcm	Idmn
**w-HLL**	glcm	JointEnergy
**w-HLL**	glcm	Contrast
**w-HLL**	glcm	DifferenceEntropy
**w-HLL**	glcm	InverseVariance
**w-HLL**	glcm	DifferenceVariance
**w-HLL**	glcm	Idn
**w-HLL**	glcm	Idm
**w-HLL**	glcm	Correlation
**w-HLL**	glcm	Autocorrelation
**w-HLL**	glcm	SumEntropy

**w-HLL**	glcm	SumSquares
**w-HLL**	glcm	ClusterProminence
**w-HLL**	glcm	Imc2
**w-HLL**	glcm	Imc1
**w-HLL**	glcm	DifferenceAverage
**w-HLL**	glcm	Id
**w-HLL**	glcm	ClusterTendency
**w-HLL**	firstorder	InterquartileRange
**w-HLL**	firstorder	Skewness
**w-HLL**	firstorder	Uniformity
**w-HLL**	firstorder	Median
**w-HLL**	firstorder	Energy
**w-HLL**	firstorder	RobustMeanAbsoluteDeviation
**w-HLL**	firstorder	MeanAbsoluteDeviation
**w-HLL**	firstorder	TotalEnergy
**w-HLL**	firstorder	Maximum
**w-HLL**	firstorder	RootMeanSquared
**w-HLL**	firstorder	90Percentile
**w-HLL**	firstorder	Minimum
**w-HLL**	firstorder	Entropy
**w-HLL**	firstorder	Range
**w-HLL**	firstorder	Variance
**w-HLL**	firstorder	10Percentile
**w-HLL**	firstorder	Kurtosis
**w-HLL**	firstorder	Mean
**w-HLL**	glrlm	ShortRunLowGrayLevelEmphasis
**w-HLL**	glrlm	GrayLevelVariance
**w-HLL**	glrlm	LowGrayLevelRunEmphasis
**w-HLL**	glrlm	GrayLevelNonUniformityNormalized
**w-HLL**	glrlm	RunVariance
**w-HLL**	glrlm	GrayLevelNonUniformity
**w-HLL**	glrlm	LongRunEmphasis
**w-HLL**	glrlm	ShortRunHighGrayLevelEmphasis
**w-HLL**	glrlm	RunLengthNonUniformity
**w-HLL**	glrlm	ShortRunEmphasis
**w-HLL**	glrlm	LongRunHighGrayLevelEmphasis
**w-HLL**	glrlm	RunPercentage
**w-HLL**	glrlm	LongRunLowGrayLevelEmphasis
**w-HLL**	glrlm	RunEntropy
**w-HLL**	glrlm	HighGrayLevelRunEmphasis
**w-HLL**	glrlm	RunLengthNonUniformityNormalized
**w-HLL**	glszm	GrayLevelVariance
**w-HLL**	glszm	ZoneVariance
**w-HLL**	glszm	GrayLevelNonUniformityNormalized
**w-HLL**	glszm	SizeZoneNonUniformityNormalized
**w-HLL**	glszm	SizeZoneNonUniformity

**w-HLL**	glszm	GrayLevelNonUniformity
**w-HLL**	glszm	LargeAreaEmphasis
**w-HLL**	glszm	SmallAreaHighGrayLevelEmphasis
**w-HLL**	glszm	ZonePercentage
**w-HLL**	glszm	LargeAreaLowGrayLevelEmphasis
**w-HLL**	glszm	LargeAreaHighGrayLevelEmphasis
**w-HLL**	glszm	HighGrayLevelZoneEmphasis
**w-HLL**	glszm	SmallAreaEmphasis
**w-HLL**	glszm	LowGrayLevelZoneEmphasis
**w-HLL**	glszm	ZoneEntropy
**w-HLL**	glszm	SmallAreaLowGrayLevelEmphasis
**w-HLL**	ngtdm	Coarseness
**w-HLL**	ngtdm	Complexity
**w-HLL**	ngtdm	Strength
**w-HLL**	ngtdm	Contrast
**w-HLL**	ngtdm	Busyness
**w-LHL**	gldm	GrayLevelVariance
**w-LHL**	gldm	HighGrayLevelEmphasis
**w-LHL**	gldm	DependenceEntropy
**w-LHL**	gldm	DependenceNonUniformity
**w-LHL**	gldm	GrayLevelNonUniformity
**w-LHL**	gldm	SmallDependenceEmphasis
**w-LHL**	gldm	SmallDependenceHighGrayLevelEmphasis
**w-LHL**	gldm	DependenceNonUniformityNormalized
**w-LHL**	gldm	LargeDependenceEmphasis
**w-LHL**	gldm	LargeDependenceLowGrayLevelEmphasis
**w-LHL**	gldm	DependenceVariance
**w-LHL**	gldm	LargeDependenceHighGrayLevelEmphasis
**w-LHL**	gldm	SmallDependenceLowGrayLevelEmphasis
**w-LHL**	gldm	LowGrayLevelEmphasis
**w-LHL**	glcm	JointAverage
**w-LHL**	glcm	SumAverage
**w-LHL**	glcm	JointEntropy
**w-LHL**	glcm	ClusterShade
**w-LHL**	glcm	MaximumProbability
**w-LHL**	glcm	Idmn
**w-LHL**	glcm	JointEnergy
**w-LHL**	glcm	Contrast
**w-LHL**	glcm	DifferenceEntropy
**w-LHL**	glcm	InverseVariance
**w-LHL**	glcm	DifferenceVariance
**w-LHL**	glcm	Idn
**w-LHL**	glcm	Idm
**w-LHL**	glcm	Correlation
**w-LHL**	glcm	Autocorrelation
**w-LHL**	glcm	SumEntropy

**w-LHL**	glcm	SumSquares
**w-LHL**	glcm	ClusterProminence
**w-LHL**	glcm	Imc2
**w-LHL**	glcm	Imc1
**w-LHL**	glcm	DifferenceAverage
**w-LHL**	glcm	Id
**w-LHL**	glcm	ClusterTendency
**w-LHL**	firstorder	InterquartileRange
**w-LHL**	firstorder	Skewness
**w-LHL**	firstorder	Uniformity
**w-LHL**	firstorder	Median
**w-LHL**	firstorder	Energy
**w-LHL**	firstorder	RobustMeanAbsoluteDeviation
**w-LHL**	firstorder	MeanAbsoluteDeviation
**w-LHL**	firstorder	TotalEnergy
**w-LHL**	firstorder	Maximum
**w-LHL**	firstorder	RootMeanSquared
**w-LHL**	firstorder	90Percentile
**w-LHL**	firstorder	Minimum
**w-LHL**	firstorder	Entropy
**w-LHL**	firstorder	Range
**w-LHL**	firstorder	Variance
**w-LHL**	firstorder	10Percentile
**w-LHL**	firstorder	Kurtosis
**w-LHL**	firstorder	Mean
**w-LHL**	glrlm	ShortRunLowGrayLevelEmphasis
**w-LHL**	glrlm	GrayLevelVariance
**w-LHL**	glrlm	LowGrayLevelRunEmphasis
**w-LHL**	glrlm	GrayLevelNonUniformityNormalized
**w-LHL**	glrlm	RunVariance
**w-LHL**	glrlm	GrayLevelNonUniformity
**w-LHL**	glrlm	LongRunEmphasis
**w-LHL**	glrlm	ShortRunHighGrayLevelEmphasis
**w-LHL**	glrlm	RunLengthNonUniformity
**w-LHL**	glrlm	ShortRunEmphasis
**w-LHL**	glrlm	LongRunHighGrayLevelEmphasis
**w-LHL**	glrlm	RunPercentage
**w-LHL**	glrlm	LongRunLowGrayLevelEmphasis
**w-LHL**	glrlm	RunEntropy
**w-LHL**	glrlm	HighGrayLevelRunEmphasis
**w-LHL**	glrlm	RunLengthNonUniformityNormalized
**w-LHL**	glszm	GrayLevelVariance
**w-LHL**	glszm	ZoneVariance
**w-LHL**	glszm	GrayLevelNonUniformityNormalized
**w-LHL**	glszm	SizeZoneNonUniformityNormalized
**w-LHL**	glszm	SizeZoneNonUniformity

**w-LHL**	glszm	GrayLevelNonUniformity
**w-LHL**	glszm	LargeAreaEmphasis
**w-LHL**	glszm	SmallAreaHighGrayLevelEmphasis
**w-LHL**	glszm	ZonePercentage
**w-LHL**	glszm	LargeAreaLowGrayLevelEmphasis
**w-LHL**	glszm	LargeAreaHighGrayLevelEmphasis
**w-LHL**	glszm	HighGrayLevelZoneEmphasis
**w-LHL**	glszm	SmallAreaEmphasis
**w-LHL**	glszm	LowGrayLevelZoneEmphasis
**w-LHL**	glszm	ZoneEntropy
**w-LHL**	glszm	SmallAreaLowGrayLevelEmphasis
**w-LHL**	ngtdm	Coarseness
**w-LHL**	ngtdm	Complexity
**w-LHL**	ngtdm	Strength
**w-LHL**	ngtdm	Contrast
**w-LHL**	ngtdm	Busyness
**w-LHH**	gldm	GrayLevelVariance
**w-LHH**	gldm	HighGrayLevelEmphasis
**w-LHH**	gldm	DependenceEntropy
**w-LHH**	gldm	DependenceNonUniformity
**w-LHH**	gldm	GrayLevelNonUniformity
**w-LHH**	gldm	SmallDependenceEmphasis
**w-LHH**	gldm	SmallDependenceHighGrayLevelEmphasis
**w-LHH**	gldm	DependenceNonUniformityNormalized
**w-LHH**	gldm	LargeDependenceEmphasis
**w-LHH**	gldm	LargeDependenceLowGrayLevelEmphasis
**w-LHH**	gldm	DependenceVariance
**w-LHH**	gldm	LargeDependenceHighGrayLevelEmphasis
**w-LHH**	gldm	SmallDependenceLowGrayLevelEmphasis
**w-LHH**	gldm	LowGrayLevelEmphasis
**w-LHH**	glcm	JointAverage
**w-LHH**	glcm	SumAverage
**w-LHH**	glcm	JointEntropy
**w-LHH**	glcm	ClusterShade
**w-LHH**	glcm	MaximumProbability
**w-LHH**	glcm	Idmn
**w-LHH**	glcm	JointEnergy
**w-LHH**	glcm	Contrast
**w-LHH**	glcm	DifferenceEntropy
**w-LHH**	glcm	InverseVariance
**w-LHH**	glcm	DifferenceVariance
**w-LHH**	glcm	Idn
**w-LHH**	glcm	Idm
**w-LHH**	glcm	Correlation
**w-LHH**	glcm	Autocorrelation
**w-LHH**	glcm	SumEntropy

**w-LHH**	glcm	SumSquares
**w-LHH**	glcm	ClusterProminence
**w-LHH**	glcm	Imc2
**w-LHH**	glcm	Imc1
**w-LHH**	glcm	DifferenceAverage
**w-LHH**	glcm	Id
**w-LHH**	glcm	ClusterTendency
**w-LHH**	firstorder	InterquartileRange
**w-LHH**	firstorder	Skewness
**w-LHH**	firstorder	Uniformity
**w-LHH**	firstorder	Median
**w-LHH**	firstorder	Energy
**w-LHH**	firstorder	RobustMeanAbsoluteDeviation
**w-LHH**	firstorder	MeanAbsoluteDeviation
**w-LHH**	firstorder	TotalEnergy
**w-LHH**	firstorder	Maximum
**w-LHH**	firstorder	RootMeanSquared
**w-LHH**	firstorder	90Percentile
**w-LHH**	firstorder	Minimum
**w-LHH**	firstorder	Entropy
**w-LHH**	firstorder	Range
**w-LHH**	firstorder	Variance
**w-LHH**	firstorder	10Percentile
**w-LHH**	firstorder	Kurtosis
**w-LHH**	firstorder	Mean
**w-LHH**	glrlm	ShortRunLowGrayLevelEmphasis
**w-LHH**	glrlm	GrayLevelVariance
**w-LHH**	glrlm	LowGrayLevelRunEmphasis
**w-LHH**	glrlm	GrayLevelNonUniformityNormalized
**w-LHH**	glrlm	RunVariance
**w-LHH**	glrlm	GrayLevelNonUniformity
**w-LHH**	glrlm	LongRunEmphasis
**w-LHH**	glrlm	ShortRunHighGrayLevelEmphasis
**w-LHH**	glrlm	RunLengthNonUniformity
**w-LHH**	glrlm	ShortRunEmphasis
**w-LHH**	glrlm	LongRunHighGrayLevelEmphasis
**w-LHH**	glrlm	RunPercentage
**w-LHH**	glrlm	LongRunLowGrayLevelEmphasis
**w-LHH**	glrlm	RunEntropy
**w-LHH**	glrlm	HighGrayLevelRunEmphasis
**w-LHH**	glrlm	RunLengthNonUniformityNormalized
**w-LHH**	glszm	GrayLevelVariance
**w-LHH**	glszm	ZoneVariance
**w-LHH**	glszm	GrayLevelNonUniformityNormalized
**w-LHH**	glszm	SizeZoneNonUniformityNormalized
**w-LHH**	glszm	SizeZoneNonUniformity

**w-LHH**	glszm	GrayLevelNonUniformity
**w-LHH**	glszm	LargeAreaEmphasis
**w-LHH**	glszm	SmallAreaHighGrayLevelEmphasis
**w-LHH**	glszm	ZonePercentage
**w-LHH**	glszm	LargeAreaLowGrayLevelEmphasis
**w-LHH**	glszm	LargeAreaHighGrayLevelEmphasis
**w-LHH**	glszm	HighGrayLevelZoneEmphasis
**w-LHH**	glszm	SmallAreaEmphasis
**w-LHH**	glszm	LowGrayLevelZoneEmphasis
**w-LHH**	glszm	ZoneEntropy
**w-LHH**	glszm	SmallAreaLowGrayLevelEmphasis
**w-LHH**	ngtdm	Coarseness
**w-LHH**	ngtdm	Complexity
**w-LHH**	ngtdm	Strength
**w-LHH**	ngtdm	Contrast
**w-LHH**	ngtdm	Busyness
**w-LLH**	gldm	GrayLevelVariance
**w-LLH**	gldm	HighGrayLevelEmphasis
**w-LLH**	gldm	DependenceEntropy
**w-LLH**	gldm	DependenceNonUniformity
**w-LLH**	gldm	GrayLevelNonUniformity
**w-LLH**	gldm	SmallDependenceEmphasis
**w-LLH**	gldm	SmallDependenceHighGrayLevelEmphasis
**w-LLH**	gldm	DependenceNonUniformityNormalized
**w-LLH**	gldm	LargeDependenceEmphasis
**w-LLH**	gldm	LargeDependenceLowGrayLevelEmphasis
**w-LLH**	gldm	DependenceVariance
**w-LLH**	gldm	LargeDependenceHighGrayLevelEmphasis
**w-LLH**	gldm	SmallDependenceLowGrayLevelEmphasis
**w-LLH**	gldm	LowGrayLevelEmphasis
**w-LLH**	glcm	JointAverage
**w-LLH**	glcm	SumAverage
**w-LLH**	glcm	JointEntropy
**w-LLH**	glcm	ClusterShade
**w-LLH**	glcm	MaximumProbability
**w-LLH**	glcm	Idmn
**w-LLH**	glcm	JointEnergy
**w-LLH**	glcm	Contrast
**w-LLH**	glcm	DifferenceEntropy
**w-LLH**	glcm	InverseVariance
**w-LLH**	glcm	DifferenceVariance
**w-LLH**	glcm	Idn
**w-LLH**	glcm	Idm
**w-LLH**	glcm	Correlation
**w-LLH**	glcm	Autocorrelation
**w-LLH**	glcm	SumEntropy

**w-LLH**	glcm	SumSquares
**w-LLH**	glcm	ClusterProminence
**w-LLH**	glcm	Imc2
**w-LLH**	glcm	Imc1
**w-LLH**	glcm	DifferenceAverage
**w-LLH**	glcm	Id
**w-LLH**	glcm	ClusterTendency
**w-LLH**	firstorder	InterquartileRange
**w-LLH**	firstorder	Skewness
**w-LLH**	firstorder	Uniformity
**w-LLH**	firstorder	Median
**w-LLH**	firstorder	Energy
**w-LLH**	firstorder	RobustMeanAbsoluteDeviation
**w-LLH**	firstorder	MeanAbsoluteDeviation
**w-LLH**	firstorder	TotalEnergy
**w-LLH**	firstorder	Maximum
**w-LLH**	firstorder	RootMeanSquared
**w-LLH**	firstorder	90Percentile
**w-LLH**	firstorder	Minimum
**w-LLH**	firstorder	Entropy
**w-LLH**	firstorder	Range
**w-LLH**	firstorder	Variance
**w-LLH**	firstorder	10Percentile
**w-LLH**	firstorder	Kurtosis
**w-LLH**	firstorder	Mean
**w-LLH**	glrlm	ShortRunLowGrayLevelEmphasis
**w-LLH**	glrlm	GrayLevelVariance
**w-LLH**	glrlm	LowGrayLevelRunEmphasis
**w-LLH**	glrlm	GrayLevelNonUniformityNormalized
**w-LLH**	glrlm	RunVariance
**w-LLH**	glrlm	GrayLevelNonUniformity
**w-LLH**	glrlm	LongRunEmphasis
**w-LLH**	glrlm	ShortRunHighGrayLevelEmphasis
**w-LLH**	glrlm	RunLengthNonUniformity
**w-LLH**	glrlm	ShortRunEmphasis
**w-LLH**	glrlm	LongRunHighGrayLevelEmphasis
**w-LLH**	glrlm	RunPercentage
**w-LLH**	glrlm	LongRunLowGrayLevelEmphasis
**w-LLH**	glrlm	RunEntropy
**w-LLH**	glrlm	HighGrayLevelRunEmphasis
**w-LLH**	glrlm	RunLengthNonUniformityNormalized
**w-LLH**	glszm	GrayLevelVariance
**w-LLH**	glszm	ZoneVariance
**w-LLH**	glszm	GrayLevelNonUniformityNormalized
**w-LLH**	glszm	SizeZoneNonUniformityNormalized
**w-LLH**	glszm	SizeZoneNonUniformity

**w-LLH**	glszm	GrayLevelNonUniformity
**w-LLH**	glszm	LargeAreaEmphasis
**w-LLH**	glszm	SmallAreaHighGrayLevelEmphasis
**w-LLH**	glszm	ZonePercentage
**w-LLH**	glszm	LargeAreaLowGrayLevelEmphasis
**w-LLH**	glszm	LargeAreaHighGrayLevelEmphasis
**w-LLH**	glszm	HighGrayLevelZoneEmphasis
**w-LLH**	glszm	SmallAreaEmphasis
**w-LLH**	glszm	LowGrayLevelZoneEmphasis
**w-LLH**	glszm	ZoneEntropy
**w-LLH**	glszm	SmallAreaLowGrayLevelEmphasis
**w-LLH**	ngtdm	Coarseness
**w-LLH**	ngtdm	Complexity
**w-LLH**	ngtdm	Strength
**w-LLH**	ngtdm	Contrast
**w-LLH**	ngtdm	Busyness
**w-LLL**	gldm	DependenceEntropy
**w-LLL**	gldm	DependenceNonUniformity
**w-LLL**	gldm	GrayLevelNonUniformity
**w-LLL**	gldm	SmallDependenceEmphasis
**w-LLL**	gldm	SmallDependenceHighGrayLevelEmphasis
**w-LLL**	gldm	DependenceNonUniformityNormalized
**w-LLL**	gldm	LargeDependenceEmphasis
**w-LLL**	gldm	LargeDependenceLowGrayLevelEmphasis
**w-LLL**	gldm	DependenceVariance
**w-LLL**	gldm	LargeDependenceHighGrayLevelEmphasis
**w-LLL**	gldm	SmallDependenceLowGrayLevelEmphasis
**w-LLL**	firstorder	InterquartileRange
**w-LLL**	firstorder	Skewness
**w-LLL**	firstorder	Median
**w-LLL**	firstorder	Energy
**w-LLL**	firstorder	RobustMeanAbsoluteDeviation
**w-LLL**	firstorder	MeanAbsoluteDeviation
**w-LLL**	firstorder	TotalEnergy
**w-LLL**	firstorder	Maximum
**w-LLL**	firstorder	RootMeanSquared
**w-LLL**	firstorder	90Percentile
**w-LLL**	firstorder	Minimum
**w-LLL**	firstorder	Range
**w-LLL**	firstorder	Variance
**w-LLL**	firstorder	10Percentile
**w-LLL**	firstorder	Kurtosis
**w-LLL**	firstorder	Mean
**w-LLL**	glrlm	ShortRunLowGrayLevelEmphasis
**w-LLL**	glrlm	RunVariance
**w-LLL**	glrlm	GrayLevelNonUniformity

**w-LLL**	glrlm	LongRunEmphasis
**w-LLL**	glrlm	ShortRunHighGrayLevelEmphasis
**w-LLL**	glrlm	RunLengthNonUniformity
**w-LLL**	glrlm	ShortRunEmphasis
**w-LLL**	glrlm	LongRunHighGrayLevelEmphasis
**w-LLL**	glrlm	RunPercentage
**w-LLL**	glrlm	LongRunLowGrayLevelEmphasis
**w-LLL**	glrlm	RunEntropy
**w-LLL**	glrlm	RunLengthNonUniformityNormalized
**w-LLL**	glszm	LargeAreaEmphasis
**w-LLL**	glszm	SmallAreaHighGrayLevelEmphasis
**w-LLL**	glszm	ZonePercentage
**w-LLL**	glszm	LargeAreaLowGrayLevelEmphasis
**w-LLL**	glszm	LargeAreaHighGrayLevelEmphasis
**w-LLL**	glszm	SmallAreaEmphasis
**w-LLL**	glszm	SmallAreaLowGrayLevelEmphasis
**w-HHL**	gldm	GrayLevelVariance
**w-HHL**	gldm	HighGrayLevelEmphasis
**w-HHL**	gldm	DependenceEntropy
**w-HHL**	gldm	DependenceNonUniformity
**w-HHL**	gldm	GrayLevelNonUniformity
**w-HHL**	gldm	SmallDependenceEmphasis
**w-HHL**	gldm	SmallDependenceHighGrayLevelEmphasis
**w-HHL**	gldm	DependenceNonUniformityNormalized
**w-HHL**	gldm	LargeDependenceEmphasis
**w-HHL**	gldm	LargeDependenceLowGrayLevelEmphasis
**w-HHL**	gldm	DependenceVariance
**w-HHL**	gldm	LargeDependenceHighGrayLevelEmphasis
**w-HHL**	gldm	SmallDependenceLowGrayLevelEmphasis
**w-HHL**	gldm	LowGrayLevelEmphasis
**w-HHL**	glcm	JointAverage
**w-HHL**	glcm	SumAverage
**w-HHL**	glcm	JointEntropy
**w-HHL**	glcm	ClusterShade
**w-HHL**	glcm	MaximumProbability
**w-HHL**	glcm	Idmn
**w-HHL**	glcm	JointEnergy
**w-HHL**	glcm	Contrast
**w-HHL**	glcm	DifferenceEntropy
**w-HHL**	glcm	InverseVariance
**w-HHL**	glcm	DifferenceVariance
**w-HHL**	glcm	Idn
**w-HHL**	glcm	Idm
**w-HHL**	glcm	Correlation
**w-HHL**	glcm	Autocorrelation
**w-HHL**	glcm	SumEntropy

**w-HHL**	glcm	SumSquares
**w-HHL**	glcm	ClusterProminence
**w-HHL**	glcm	Imc2
**w-HHL**	glcm	Imc1
**w-HHL**	glcm	DifferenceAverage
**w-HHL**	glcm	Id
**w-HHL**	glcm	ClusterTendency
**w-HHL**	firstorder	InterquartileRange
**w-HHL**	firstorder	Skewness
**w-HHL**	firstorder	Uniformity
**w-HHL**	firstorder	Median
**w-HHL**	firstorder	Energy
**w-HHL**	firstorder	RobustMeanAbsoluteDeviation
**w-HHL**	firstorder	MeanAbsoluteDeviation
**w-HHL**	firstorder	TotalEnergy
**w-HHL**	firstorder	Maximum
**w-HHL**	firstorder	RootMeanSquared
**w-HHL**	firstorder	90Percentile
**w-HHL**	firstorder	Minimum
**w-HHL**	firstorder	Entropy
**w-HHL**	firstorder	Range
**w-HHL**	firstorder	Variance
**w-HHL**	firstorder	10Percentile
**w-HHL**	firstorder	Kurtosis
**w-HHL**	firstorder	Mean
**w-HHL**	glrlm	ShortRunLowGrayLevelEmphasis
**w-HHL**	glrlm	GrayLevelVariance
**w-HHL**	glrlm	LowGrayLevelRunEmphasis
**w-HHL**	glrlm	GrayLevelNonUniformityNormalized
**w-HHL**	glrlm	RunVariance
**w-HHL**	glrlm	GrayLevelNonUniformity
**w-HHL**	glrlm	LongRunEmphasis
**w-HHL**	glrlm	ShortRunHighGrayLevelEmphasis
**w-HHL**	glrlm	RunLengthNonUniformity
**w-HHL**	glrlm	ShortRunEmphasis
**w-HHL**	glrlm	LongRunHighGrayLevelEmphasis
**w-HHL**	glrlm	RunPercentage
**w-HHL**	glrlm	LongRunLowGrayLevelEmphasis
**w-HHL**	glrlm	RunEntropy
**w-HHL**	glrlm	HighGrayLevelRunEmphasis
**w-HHL**	glrlm	RunLengthNonUniformityNormalized
**w-HHL**	glszm	GrayLevelVariance
**w-HHL**	glszm	ZoneVariance
**w-HHL**	glszm	GrayLevelNonUniformityNormalized
**w-HHL**	glszm	SizeZoneNonUniformityNormalized
**w-HHL**	glszm	SizeZoneNonUniformity

**w-HHL**	glszm	GrayLevelNonUniformity
**w-HHL**	glszm	LargeAreaEmphasis
**w-HHL**	glszm	SmallAreaHighGrayLevelEmphasis
**w-HHL**	glszm	ZonePercentage
**w-HHL**	glszm	LargeAreaLowGrayLevelEmphasis
**w-HHL**	glszm	LargeAreaHighGrayLevelEmphasis
**w-HHL**	glszm	HighGrayLevelZoneEmphasis
**w-HHL**	glszm	SmallAreaEmphasis
**w-HHL**	glszm	LowGrayLevelZoneEmphasis
**w-HHL**	glszm	ZoneEntropy
**w-HHL**	glszm	SmallAreaLowGrayLevelEmphasis
**w-HHL**	ngtdm	Coarseness
**w-HHL**	ngtdm	Complexity
**w-HHL**	ngtdm	Strength
**w-HHL**	ngtdm	Contrast
**w-HHL**	ngtdm	Busyness
**w-HLH**	gldm	GrayLevelVariance
**w-HLH**	gldm	HighGrayLevelEmphasis
**w-HLH**	gldm	DependenceEntropy
**w-HLH**	gldm	DependenceNonUniformity
**w-HLH**	gldm	GrayLevelNonUniformity
**w-HLH**	gldm	SmallDependenceEmphasis
**w-HLH**	gldm	SmallDependenceHighGrayLevelEmphasis
**w-HLH**	gldm	DependenceNonUniformityNormalized
**w-HLH**	gldm	LargeDependenceEmphasis
**w-HLH**	gldm	LargeDependenceLowGrayLevelEmphasis
**w-HLH**	gldm	DependenceVariance
**w-HLH**	gldm	LargeDependenceHighGrayLevelEmphasis
**w-HLH**	gldm	SmallDependenceLowGrayLevelEmphasis
**w-HLH**	gldm	LowGrayLevelEmphasis
**w-HLH**	glcm	JointAverage
**w-HLH**	glcm	SumAverage
**w-HLH**	glcm	JointEntropy
**w-HLH**	glcm	ClusterShade
**w-HLH**	glcm	MaximumProbability
**w-HLH**	glcm	Idmn
**w-HLH**	glcm	JointEnergy
**w-HLH**	glcm	Contrast
**w-HLH**	glcm	DifferenceEntropy
**w-HLH**	glcm	InverseVariance
**w-HLH**	glcm	DifferenceVariance
**w-HLH**	glcm	Idn
**w-HLH**	glcm	Idm
**w-HLH**	glcm	Correlation
**w-HLH**	glcm	Autocorrelation
**w-HLH**	glcm	SumEntropy

**w-HLH**	glcm	SumSquares
**w-HLH**	glcm	ClusterProminence
**w-HLH**	glcm	Imc2
**w-HLH**	glcm	Imc1
**w-HLH**	glcm	DifferenceAverage
**w-HLH**	glcm	Id
**w-HLH**	glcm	ClusterTendency
**w-HLH**	firstorder	InterquartileRange
**w-HLH**	firstorder	Skewness
**w-HLH**	firstorder	Uniformity
**w-HLH**	firstorder	Median
**w-HLH**	firstorder	Energy
**w-HLH**	firstorder	RobustMeanAbsoluteDeviation
**w-HLH**	firstorder	MeanAbsoluteDeviation
**w-HLH**	firstorder	TotalEnergy
**w-HLH**	firstorder	Maximum
**w-HLH**	firstorder	RootMeanSquared
**w-HLH**	firstorder	90Percentile
**w-HLH**	firstorder	Minimum
**w-HLH**	firstorder	Entropy
**w-HLH**	firstorder	Range
**w-HLH**	firstorder	Variance
**w-HLH**	firstorder	10Percentile
**w-HLH**	firstorder	Kurtosis
**w-HLH**	firstorder	Mean
**w-HLH**	glrlm	ShortRunLowGrayLevelEmphasis
**w-HLH**	glrlm	GrayLevelVariance
**w-HLH**	glrlm	LowGrayLevelRunEmphasis
**w-HLH**	glrlm	GrayLevelNonUniformityNormalized
**w-HLH**	glrlm	RunVariance
**w-HLH**	glrlm	GrayLevelNonUniformity
**w-HLH**	glrlm	LongRunEmphasis
**w-HLH**	glrlm	ShortRunHighGrayLevelEmphasis
**w-HLH**	glrlm	RunLengthNonUniformity
**w-HLH**	glrlm	ShortRunEmphasis
**w-HLH**	glrlm	LongRunHighGrayLevelEmphasis
**w-HLH**	glrlm	RunPercentage
**w-HLH**	glrlm	LongRunLowGrayLevelEmphasis
**w-HLH**	glrlm	RunEntropy
**w-HLH**	glrlm	HighGrayLevelRunEmphasis
**w-HLH**	glrlm	RunLengthNonUniformityNormalized
**w-HLH**	glszm	GrayLevelVariance
**w-HLH**	glszm	ZoneVariance
**w-HLH**	glszm	GrayLevelNonUniformityNormalized
**w-HLH**	glszm	SizeZoneNonUniformityNormalized
**w-HLH**	glszm	SizeZoneNonUniformity

**w-HLH**	glszm	GrayLevelNonUniformity
**w-HLH**	glszm	LargeAreaEmphasis
**w-HLH**	glszm	SmallAreaHighGrayLevelEmphasis
**w-HLH**	glszm	ZonePercentage
**w-HLH**	glszm	LargeAreaLowGrayLevelEmphasis
**w-HLH**	glszm	LargeAreaHighGrayLevelEmphasis
**w-HLH**	glszm	HighGrayLevelZoneEmphasis
**w-HLH**	glszm	SmallAreaEmphasis
**w-HLH**	glszm	LowGrayLevelZoneEmphasis
**w-HLH**	glszm	ZoneEntropy
**w-HLH**	glszm	SmallAreaLowGrayLevelEmphasis
**w-HLH**	ngtdm	Coarseness
**w-HLH**	ngtdm	Complexity
**w-HLH**	ngtdm	Strength
**w-HLH**	ngtdm	Contrast
**w-HLH**	ngtdm	Busyness

Supplementary Table 2. ICC values of radiomics features extracted in the current study before combat NPNE harmonization.

Set	Type	Name	value
**Original**	firstorder	Maximum	0.999755
**w-LLL**	firstorder	Maximum	0.997979
**Original**	firstorder	90Percentile	0.996699
**w-LLL**	firstorder	90Percentile	0.995462
**Original**	firstorder	Energy	0.993831
**Original**	firstorder	TotalEnergy	0.993831
**w-LLL**	firstorder	Energy	0.993254
**w-LLL**	firstorder	TotalEnergy	0.993254
**w-LLL**	firstorder	Range	0.992777
**Original**	firstorder	Range	0.992466
**Original**	firstorder	RootMeanSquared	0.991577
**w-LLL**	firstorder	RootMeanSquared	0.990885
**w-LLL**	firstorder	MeanAbsoluteDeviation	0.989573
**Original**	firstorder	MeanAbsoluteDeviation	0.988979
**Original**	firstorder	Mean	0.988661
**w-LLL**	firstorder	Mean	0.988509
**w-LLL**	firstorder	RobustMeanAbsoluteDeviation	0.988257
**w-LLL**	firstorder	InterquartileRange	0.987772
**w-LHL**	firstorder	InterquartileRange	0.987439
**w-LHL**	firstorder	RobustMeanAbsoluteDeviation	0.987106
**w-LHL**	firstorder	Minimum	0.986955
**w-LHL**	firstorder	Range	0.986891
**Original**	firstorder	RobustMeanAbsoluteDeviation	0.986776
**w-LHL**	firstorder	MeanAbsoluteDeviation	0.986053
**Original**	firstorder	InterquartileRange	0.986009
**w-LLL**	firstorder	Median	0.985942
**w-LHL**	firstorder	Maximum	0.985817
**Original**	firstorder	Median	0.985453
**w-LLH**	firstorder	RobustMeanAbsoluteDeviation	0.98481
**w-LLH**	firstorder	InterquartileRange	0.983677
**w-HLL**	firstorder	Range	0.981712
**w-HLL**	firstorder	Variance	0.981238
**w-HLL**	firstorder	Maximum	0.980778
**w-LLH**	firstorder	MeanAbsoluteDeviation	0.979212
**w-HLL**	firstorder	RootMeanSquared	0.979206
**w-LLH**	firstorder	Range	0.978701
**w-LHL**	firstorder	RootMeanSquared	0.978512
**w-HLL**	firstorder	MeanAbsoluteDeviation	0.978481
**w-LLH**	firstorder	Minimum	0.978146
**w-LLH**	firstorder	Maximum	0.977534
**w-HLL**	firstorder	RobustMeanAbsoluteDeviation	0.977416
**w-LHL**	firstorder	10Percentile	0.977102

**w-LLH**	firstorder	10Percentile	0.9771
**w-LLL**	firstorder	Variance	0.9769
**w-HLL**	firstorder	InterquartileRange	0.976545
**w-HLL**	firstorder	Minimum	0.976268
**w-HLL**	firstorder	Energy	0.976253
**w-HLL**	firstorder	TotalEnergy	0.976253
**w-HLL**	firstorder	90Percentile	0.975529
**Original**	firstorder	Variance	0.975257
**w-HHL**	firstorder	Minimum	0.974436
**w-HHL**	firstorder	Range	0.974165
**w-HHL**	firstorder	Energy	0.974077
**w-HHL**	firstorder	TotalEnergy	0.974069
**w-HHL**	firstorder	Maximum	0.97357
**w-LHL**	firstorder	Energy	0.973091
**w-LHL**	firstorder	TotalEnergy	0.973073
**w-LLH**	glrlm	LongRunLowGrayLevelEmphasis	0.971722
**w-LLL**	firstorder	10Percentile	0.970559
**Original**	firstorder	10Percentile	0.968223
**w-HHL**	firstorder	RobustMeanAbsoluteDeviation	0.968153
**w-HHL**	firstorder	InterquartileRange	0.968105
**w-LHL**	firstorder	Variance	0.968009
**w-HLH**	firstorder	Maximum	0.967115
**w-HHL**	firstorder	Variance	0.967085
**w-LLH**	glrlm	RunVariance	0.967028
**w-HLH**	firstorder	Range	0.96619
**w-HHL**	firstorder	90Percentile	0.965258
**w-LHL**	glrlm	LongRunLowGrayLevelEmphasis	0.964801
**w-LLH**	firstorder	RootMeanSquared	0.963579
**w-HHL**	firstorder	RootMeanSquared	0.963365
**w-LLH**	glrlm	LongRunEmphasis	0.962844
**w-HLH**	firstorder	Minimum	0.96256
**w-HHL**	firstorder	MeanAbsoluteDeviation	0.961975
**w-LHL**	gldm	DependenceNonUniformityNormalized	0.960559
**w-HLH**	firstorder	Energy	0.957265
**w-HLH**	firstorder	TotalEnergy	0.957246
**Original**	glszm	LargeAreaEmphasis	0.956279
**Original**	glszm	LargeAreaLowGrayLevelEmphasis	0.956279
**Original**	glszm	LargeAreaHighGrayLevelEmphasis	0.956279
**w-LLL**	glszm	LargeAreaEmphasis	0.956279
**w-LLL**	glszm	LargeAreaLowGrayLevelEmphasis	0.956279
**w-LLL**	glszm	LargeAreaHighGrayLevelEmphasis	0.956279
**w-HLL**	glszm	LargeAreaLowGrayLevelEmphasis	0.955082
**w-HLL**	firstorder	10Percentile	0.954346
**w-LHL**	glrlm	RunVariance	0.952822
**w-HLL**	glszm	ZoneVariance	0.951765
**w-HHL**	firstorder	10Percentile	0.951143

**w-HHH**	gldm	DependenceNonUniformityNormalized	0.948964
**w-LHL**	glrlm	LongRunEmphasis	0.948837
**w-LLH**	firstorder	Energy	0.948813
**w-LLH**	firstorder	TotalEnergy	0.948762
**w-LHL**	gldm	DependenceEntropy	0.948714
**w-HLH**	firstorder	InterquartileRange	0.94705
**w-HLL**	glszm	LargeAreaEmphasis	0.947018
**w-LLH**	gldm	DependenceNonUniformityNormalized	0.946805
**w-HLH**	firstorder	RobustMeanAbsoluteDeviation	0.946072
**w-HHH**	gldm	DependenceEntropy	0.944562
**w-LHH**	gldm	DependenceNonUniformityNormalized	0.943389
**w-HLH**	firstorder	MeanAbsoluteDeviation	0.941099
**w-LLH**	gldm	DependenceEntropy	0.940823
**w-HLL**	glrlm	LongRunLowGrayLevelEmphasis	0.940651
**w-HLH**	glrlm	LongRunHighGrayLevelEmphasis	0.940425
**w-LLH**	firstorder	90Percentile	0.94025
**w-HHH**	glrlm	RunVariance	0.94018
**w-HHH**	glrlm	LongRunHighGrayLevelEmphasis	0.939779
**w-LLH**	gldm	LargeDependenceLowGrayLevelEmphasis	0.939504
**w-LHH**	gldm	DependenceVariance	0.93879
**w-HLH**	glrlm	RunVariance	0.938735
**w-HLH**	glrlm	LongRunEmphasis	0.938448
**w-HHH**	gldm	DependenceVariance	0.938396
**w-HHH**	glrlm	LongRunEmphasis	0.938353
**w-LHL**	firstorder	Mean	0.936523
**w-LHL**	gldm	LargeDependenceLowGrayLevelEmphasis	0.936106
**w-HHH**	glrlm	LongRunLowGrayLevelEmphasis	0.935662
**w-HLH**	firstorder	RootMeanSquared	0.935553
**w-HLH**	gldm	DependenceNonUniformity	0.935049
**w-HLL**	firstorder	Median	0.934366
**w-LLH**	firstorder	Mean	0.934348
**w-LLH**	gldm	LargeDependenceEmphasis	0.933683
**w-LHH**	glrlm	RunVariance	0.933634
**w-HLH**	glszm	SizeZoneNonUniformity	0.933185
**w-LHL**	gldm	DependenceNonUniformity	0.931222
**w-HHH**	firstorder	Energy	0.931035
**w-HLL**	glszm	LargeAreaHighGrayLevelEmphasis	0.931019
**w-HHH**	firstorder	TotalEnergy	0.930931
**w-LHH**	firstorder	InterquartileRange	0.930906
**w-HLH**	glrlm	LongRunLowGrayLevelEmphasis	0.930719
**w-LHL**	gldm	LargeDependenceEmphasis	0.930348
**w-HLL**	firstorder	Mean	0.929911
**w-LHH**	glrlm	LongRunHighGrayLevelEmphasis	0.928974
**w-HLH**	firstorder	90Percentile	0.928542
**w-HLH**	firstorder	10Percentile	0.928335
**w-LLH**	glrlm	RunPercentage	0.928216

**w-LHH**	firstorder	Energy	0.927828
**w-LHH**	firstorder	TotalEnergy	0.927707
**w-LHH**	glrlm	LongRunEmphasis	0.927521
**w-LHH**	firstorder	RobustMeanAbsoluteDeviation	0.926552
**w-HHL**	gldm	DependenceNonUniformity	0.926262
**w-LHL**	glrlm	RunPercentage	0.925052
**w-LLH**	gldm	DependenceNonUniformity	0.924894
**w-HLL**	gldm	LargeDependenceLowGrayLevelEmphasis	0.924282
**w-LHH**	glrlm	LongRunLowGrayLevelEmphasis	0.923804
**w-LHH**	gldm	DependenceEntropy	0.92362
**w-LHH**	glszm	LargeAreaLowGrayLevelEmphasis	0.9229
**w-LLH**	glszm	SizeZoneNonUniformity	0.922764
**w-LHL**	firstorder	Median	0.922242
**w-HHL**	glrlm	LongRunHighGrayLevelEmphasis	0.92166
**w-LHH**	glszm	LargeAreaEmphasis	0.921203
**w-LHL**	ngtdm	Contrast	0.920901
**w-HHL**	glrlm	RunVariance	0.920743
**w-LHH**	glszm	ZoneVariance	0.920657
**w-LLH**	glcm	DifferenceVariance	0.920075
**w-LLH**	glcm	Idmn	0.919463
**w-LLH**	glcm	Idm	0.919463
**w-LLH**	glcm	Id	0.919463
**w-LLH**	glcm	Contrast	0.919463
**w-LLH**	glcm	InverseVariance	0.919463
**w-LLH**	glcm	DifferenceAverage	0.919463
**w-LLH**	glcm	Idn	0.919463
**w-LHH**	glszm	LargeAreaHighGrayLevelEmphasis	0.919397
**Original**	glrlm	LongRunEmphasis	0.919022
**Original**	glrlm	LongRunHighGrayLevelEmphasis	0.919022
**Original**	glrlm	LongRunLowGrayLevelEmphasis	0.919022
**w-LLL**	glrlm	LongRunEmphasis	0.919022
**w-LLL**	glrlm	LongRunHighGrayLevelEmphasis	0.919022
**w-LLL**	glrlm	LongRunLowGrayLevelEmphasis	0.919022
**w-HLL**	gldm	DependenceVariance	0.91801
**w-LLH**	glcm	DifferenceEntropy	0.917969
**w-LHH**	glrlm	RunLengthNonUniformity	0.917418
**w-HHL**	glrlm	LongRunEmphasis	0.916717
**w-LLH**	firstorder	Median	0.916058
**w-LLH**	firstorder	Variance	0.915792
**w-LHL**	glszm	LargeAreaLowGrayLevelEmphasis	0.915737
**w-LHH**	glszm	SizeZoneNonUniformity	0.915651
**w-HLH**	gldm	DependenceNonUniformityNormalized	0.914384
**w-HHH**	gldm	DependenceNonUniformity	0.91369
**w-LHL**	glcm	DifferenceVariance	0.912288
**w-LHL**	glcm	Idm	0.91102
**w-LHL**	glcm	Id	0.91102

**w-LHL**	glcm	Idn	0.91102
**w-LHL**	glcm	Contrast	0.91102
**w-LHL**	glcm	InverseVariance	0.91102
**w-LHL**	glcm	DifferenceAverage	0.91102
**w-LHL**	glcm	Idmn	0.91102
**w-LLH**	glrlm	LongRunHighGrayLevelEmphasis	0.909798
**Original**	glrlm	RunVariance	0.909575
**w-LLL**	glrlm	RunVariance	0.909575
**w-HLL**	glrlm	LongRunEmphasis	0.909348
**w-HHH**	glszm	LargeAreaHighGrayLevelEmphasis	0.908815
**w-HLL**	gldm	DependenceNonUniformity	0.90833
**w-LHH**	ngtdm	Busyness	0.907841
**w-HHL**	glrlm	LongRunLowGrayLevelEmphasis	0.907414
**w-HHH**	glszm	ZoneVariance	0.907378
**w-HHH**	glszm	LargeAreaEmphasis	0.907233
**w-LHL**	glcm	DifferenceEntropy	0.906865
**w-HHH**	glszm	LargeAreaLowGrayLevelEmphasis	0.905476
**w-LHH**	gldm	DependenceNonUniformity	0.903903
**w-HHH**	glrlm	RunLengthNonUniformity	0.90345
**Original**	shape	LeastAxis	0.903128
**Original**	glrlm	RunEntropy	0.902515
**w-LLL**	glrlm	RunEntropy	0.902515
**w-HLL**	glrlm	RunVariance	0.902088
**w-LHL**	glszm	SizeZoneNonUniformity	0.901347
**w-HHH**	firstorder	Minimum	0.90057
**w-HHL**	glszm	SizeZoneNonUniformity	0.90039
**w-LLH**	gldm	GrayLevelNonUniformity	0.899486
**w-HLH**	glszm	GrayLevelNonUniformity	0.898997
**w-HHH**	firstorder	Range	0.898527
**w-LHH**	firstorder	10Percentile	0.898053
**w-HLL**	gldm	DependenceNonUniformityNormalized	0.897806
**w-LHL**	firstorder	90Percentile	0.896794
**w-LHL**	glszm	ZoneVariance	0.89559
**w-HLL**	glrlm	ShortRunEmphasis	0.895449
**w-LHL**	glszm	LargeAreaEmphasis	0.895193
**w-LHL**	gldm	GrayLevelNonUniformity	0.895101
**w-LHH**	glrlm	GrayLevelNonUniformity	0.894515
**w-HHL**	gldm	DependenceNonUniformityNormalized	0.894287
**w-HHH**	firstorder	Maximum	0.893995
**Original**	gldm	DependenceNonUniformity	0.893211
**w-LLL**	gldm	DependenceNonUniformity	0.893211
**w-LLL**	firstorder	Minimum	0.892389
**w-HLL**	glcm	ClusterShade	0.892368
**w-LHH**	firstorder	MeanAbsoluteDeviation	0.891315
**w-HLH**	glrlm	RunEntropy	0.889195
**Original**	gldm	DependenceVariance	0.889002

**w-LLL**	gldm	DependenceVariance	0.889002
**w-LHL**	glrlm	LongRunHighGrayLevelEmphasis	0.888452
**w-HLL**	glrlm	RunEntropy	0.888294
**w-LLH**	glszm	GrayLevelNonUniformity	0.887989
**w-HHL**	glrlm	RunEntropy	0.887595
**w-LHH**	firstorder	Minimum	0.887519
**w-LHL**	glrlm	RunLengthNonUniformityNormalized	0.887492
**w-LHL**	firstorder	Kurtosis	0.887299
**Original**	firstorder	Minimum	0.886794
**Original**	shape	Flatness	0.88673
**w-LHH**	firstorder	Range	0.886245
**w-LLH**	glrlm	RunLengthNonUniformityNormalized	0.886015
**w-LHL**	gldm	DependenceVariance	0.885973
**w-HHH**	glrlm	GrayLevelNonUniformity	0.885962
**w-HLL**	glrlm	RunLengthNonUniformityNormalized	0.884625
**Original**	firstorder	Skewness	0.884561
**w-HHL**	glszm	LargeAreaLowGrayLevelEmphasis	0.883828
**w-HLH**	gldm	GrayLevelNonUniformity	0.883107
**w-HHH**	glszm	SizeZoneNonUniformity	0.88307
**w-HLL**	gldm	GrayLevelNonUniformity	0.882191
**w-HLH**	gldm	DependenceEntropy	0.882025
**w-HHH**	ngtdm	Busyness	0.881967
**Original**	gldm	DependenceNonUniformityNormalized	0.881187
**w-LLL**	gldm	DependenceNonUniformityNormalized	0.881187
**w-HHL**	gldm	GrayLevelNonUniformity	0.881142
**w-HHH**	gldm	GrayLevelNonUniformity	0.881122
**Original**	gldm	GrayLevelNonUniformity	0.881072
**w-LLL**	gldm	GrayLevelNonUniformity	0.881072
**w-LHH**	gldm	GrayLevelNonUniformity	0.880944
**w-LLH**	glszm	LargeAreaLowGrayLevelEmphasis	0.880875
**w-HLL**	gldm	DependenceEntropy	0.880873
**w-HLL**	glrlm	LongRunHighGrayLevelEmphasis	0.88042
**w-HLL**	gldm	LargeDependenceEmphasis	0.880372
**Original**	shape	Volume	0.880202
**w-LHL**	glrlm	RunEntropy	0.880128
**w-HLH**	glrlm	ShortRunEmphasis	0.879746
**w-HHL**	glszm	LargeAreaEmphasis	0.879548
**w-HLL**	glrlm	RunPercentage	0.879532
**w-HHL**	glrlm	ShortRunEmphasis	0.879183
**w-HHL**	glszm	ZoneVariance	0.878959
**w-LHH**	firstorder	Maximum	0.877896
**w-HLL**	gldm	SmallDependenceHighGrayLevelEmphasis	0.876589
**w-HLL**	glcm	SumAverage	0.875537
**w-HLL**	glcm	JointAverage	0.875537
**w-LLH**	glrlm	RunEntropy	0.875475
**w-LHH**	firstorder	RootMeanSquared	0.874539

**w-HHL**	glrlm	ShortRunLowGrayLevelEmphasis	0.873843
**w-HLL**	gldm	HighGrayLevelEmphasis	0.873812
**w-HLL**	gldm	LowGrayLevelEmphasis	0.873812
**w-HHL**	glszm	LargeAreaHighGrayLevelEmphasis	0.873804
**w-LLH**	gldm	DependenceVariance	0.873768
**w-HLL**	glrlm	ShortRunHighGrayLevelEmphasis	0.873715
**w-HLL**	glcm	Autocorrelation	0.873078
**w-HLH**	glrlm	ShortRunHighGrayLevelEmphasis	0.872113
**w-LHH**	firstorder	90Percentile	0.871263
**w-HHL**	gldm	LargeDependenceHighGrayLevelEmphasis	0.870808
**w-HLH**	glrlm	ShortRunLowGrayLevelEmphasis	0.870587
**w-HHL**	glrlm	ShortRunHighGrayLevelEmphasis	0.870469
**Original**	gldm	DependenceEntropy	0.869768
**w-LLL**	gldm	DependenceEntropy	0.869768
**w-HLH**	gldm	LargeDependenceHighGrayLevelEmphasis	0.869576
**w-HHH**	glrlm	RunEntropy	0.86949
**w-LHL**	glrlm	ShortRunEmphasis	0.869461
**w-LLL**	firstorder	Skewness	0.867265
**w-HLH**	gldm	DependenceVariance	0.866779
**w-LLH**	ngtdm	Contrast	0.866342
**Original**	shape	Maximum2DDiameterColumn	0.865257
**Original**	shape	Sphericity	0.864656
**w-HLH**	firstorder	Median	0.863979
**w-HHL**	gldm	LargeDependenceEmphasis	0.863053
**w-HHL**	gldm	DependenceEntropy	0.861645
**w-HHH**	glcm	Imc2	0.86035
**w-HLH**	gldm	LargeDependenceEmphasis	0.859867
**w-HHH**	glcm	Idmn	0.859418
**w-HHH**	glcm	Idm	0.859418
**w-HHH**	glcm	Id	0.859418
**w-HHH**	glcm	Idn	0.859418
**w-HHH**	glcm	Contrast	0.859418
**w-HHH**	glcm	InverseVariance	0.859418
**w-HHH**	glcm	DifferenceAverage	0.859418
**w-HHH**	firstorder	RootMeanSquared	0.858568
**w-HLH**	firstorder	Variance	0.858282
**w-HHL**	glrlm	RunPercentage	0.858212
**w-HLL**	gldm	SmallDependenceEmphasis	0.858004
**w-HHH**	gldm	LargeDependenceEmphasis	0.857262
**w-HLL**	glcm	Idm	0.856802
**w-HLL**	glcm	Id	0.856802
**w-HLL**	glcm	Contrast	0.856802
**w-HLL**	glcm	InverseVariance	0.856802
**w-HLL**	glcm	DifferenceAverage	0.856802
**w-HLL**	glcm	Idn	0.856802
**w-HLL**	glcm	Idmn	0.856802

**w-HHH**	glcm	DifferenceEntropy	0.855584
**w-HHH**	glcm	Imc1	0.855363
**w-HHH**	gldm	LargeDependenceHighGrayLevelEmphasis	0.854965
**w-HLH**	glrlm	RunPercentage	0.854938
**w-HHH**	glcm	Correlation	0.854708
**w-HLL**	glcm	DifferenceVariance	0.854693
**w-HHL**	glrlm	RunLengthNonUniformityNormalized	0.854516
**w-LHL**	glszm	LargeAreaHighGrayLevelEmphasis	0.854282
**w-HLL**	gldm	LargeDependenceHighGrayLevelEmphasis	0.854096
**w-HHH**	glcm	ClusterProminence	0.853252
**w-HHH**	glcm	DifferenceVariance	0.853198
**w-HLH**	glrlm	RunLengthNonUniformityNormalized	0.852068
**w-HHL**	firstorder	Mean	0.851974
**w-LHL**	glszm	GrayLevelNonUniformity	0.851952
**w-HHH**	glcm	SumEntropy	0.851738
**w-LHH**	glrlm	RunEntropy	0.850663
**w-LLH**	glrlm	GrayLevelNonUniformity	0.849765
**w-HLL**	glcm	DifferenceEntropy	0.849655
**w-LHH**	glcm	Imc2	0.848703
**w-LLH**	glcm	MaximumProbability	0.848685
**w-LLH**	glszm	ZoneVariance	0.848524
**w-LLH**	glcm	JointEntropy	0.848346
**w-LLH**	glrlm	ShortRunEmphasis	0.847905
**w-LLH**	glszm	LargeAreaEmphasis	0.846659
**w-HHL**	firstorder	Median	0.846023
**Original**	glrlm	RunLengthNonUniformityNormalized	0.845689
**w-LLL**	glrlm	RunLengthNonUniformityNormalized	0.845689
**w-HHH**	glcm	ClusterTendency	0.84504
**w-HHH**	glcm	JointEntropy	0.844883
**Original**	shape	Elongation	0.8446
**w-HHL**	gldm	DependenceVariance	0.84433
**w-LHL**	glrlm	GrayLevelNonUniformity	0.843728
**w-HLL**	glszm	HighGrayLevelZoneEmphasis	0.843685
**w-HLL**	glszm	LowGrayLevelZoneEmphasis	0.843685
**w-LHH**	glcm	Idm	0.842903
**w-LHH**	glcm	Id	0.842903
**w-LHH**	glcm	Idmn	0.842903
**w-LHH**	glcm	Contrast	0.842903
**w-LHH**	glcm	InverseVariance	0.842903
**w-LHH**	glcm	DifferenceAverage	0.842903
**w-LHH**	glcm	Idn	0.842903
**w-LLH**	glcm	JointEnergy	0.842215
**w-HLH**	firstorder	Mean	0.841827
**w-HLH**	glcm	DifferenceEntropy	0.841514
**w-LHL**	ngtdm	Complexity	0.841485
**w-HLH**	glcm	DifferenceVariance	0.841458

**w-LHH**	gldm	LargeDependenceEmphasis	0.841231
**Original**	gldm	LargeDependenceEmphasis	0.840046
**Original**	gldm	LargeDependenceLowGrayLevelEmphasis	0.840046
**Original**	gldm	LargeDependenceHighGrayLevelEmphasis	0.840046
**w-LLL**	gldm	LargeDependenceEmphasis	0.840046
**w-LLL**	gldm	LargeDependenceLowGrayLevelEmphasis	0.840046
**w-LLL**	gldm	LargeDependenceHighGrayLevelEmphasis	0.840046
**w-HHH**	glrlm	RunPercentage	0.838582
**Original**	firstorder	Kurtosis	0.838101
**Original**	glrlm	RunPercentage	0.836864
**w-LLL**	glrlm	RunPercentage	0.836864
**w-HLL**	glrlm	ShortRunLowGrayLevelEmphasis	0.836408
**w-HHH**	gldm	LargeDependenceLowGrayLevelEmphasis	0.836288
**w-LLL**	firstorder	Kurtosis	0.836163
**w-LHH**	glcm	Correlation	0.836068
**Original**	shape	Maximum2DDiameterRow	0.835499
**w-HHL**	glrlm	GrayLevelNonUniformity	0.835123
**w-LHH**	glcm	ClusterProminence	0.835019
**w-HHH**	glcm	JointEnergy	0.835006
**w-LHH**	glrlm	ShortRunLowGrayLevelEmphasis	0.834774
**w-LLH**	glrlm	RunLengthNonUniformity	0.834525
**w-HLL**	glszm	ZonePercentage	0.83423
**w-HHL**	gldm	LargeDependenceLowGrayLevelEmphasis	0.834191
**w-LHH**	gldm	LargeDependenceHighGrayLevelEmphasis	0.833172
**w-LHH**	gldm	LargeDependenceLowGrayLevelEmphasis	0.832708
**w-HHH**	glrlm	RunLengthNonUniformityNormalized	0.832458
**w-LHL**	glszm	ZoneEntropy	0.832092
**w-LHL**	glcm	JointEntropy	0.831294
**Original**	glrlm	GrayLevelNonUniformity	0.830695
**w-LLL**	glrlm	GrayLevelNonUniformity	0.830695
**w-HLH**	glcm	ClusterShade	0.830466
**w-HHL**	glcm	ClusterShade	0.830384
**w-HHH**	glrlm	ShortRunEmphasis	0.830188
**Original**	shape	SurfaceArea	0.829776
**w-LHH**	glrlm	RunPercentage	0.829667
**w-HLH**	glcm	Idmn	0.829507
**w-HLH**	glcm	Contrast	0.829507
**w-HLH**	glcm	InverseVariance	0.829507
**w-HLH**	glcm	DifferenceAverage	0.829507
**w-HLH**	glcm	Idm	0.829507
**w-HLH**	glcm	Id	0.829507
**w-HLH**	glcm	Idn	0.829507
**w-HHH**	glcm	MaximumProbability	0.829342
**w-HHH**	glrlm	ShortRunLowGrayLevelEmphasis	0.82819
**w-HHL**	glcm	Imc1	0.828093
**w-LLH**	glcm	SumEntropy	0.827879

**w-HLH**	glcm	Imc1	0.82647
**w-LHH**	glrlm	ShortRunEmphasis	0.826279
**w-LHL**	glcm	JointEnergy	0.826235
**w-HLH**	glrlm	GrayLevelNonUniformity	0.825836
**w-HLH**	gldm	SmallDependenceHighGrayLevelEmphasis	0.82554
**w-HHL**	glcm	DifferenceVariance	0.824527
**w-LHL**	glcm	MaximumProbability	0.8243
**w-LHL**	gldm	SmallDependenceEmphasis	0.824118
**w-LHH**	ngtdm	Strength	0.823782
**w-LHH**	ngtdm	Coarseness	0.823771
**w-HLH**	glszm	LargeAreaLowGrayLevelEmphasis	0.823761
**w-HHH**	glrlm	ShortRunHighGrayLevelEmphasis	0.823666
**w-LLH**	glcm	JointAverage	0.823527
**w-LLH**	glcm	SumAverage	0.823527
**w-LLH**	glrlm	ShortRunLowGrayLevelEmphasis	0.822233
**w-LHL**	gldm	SmallDependenceHighGrayLevelEmphasis	0.821875
**w-LHH**	glcm	MaximumProbability	0.821869
**w-HHL**	glcm	DifferenceEntropy	0.821805
**w-LHL**	gldm	SmallDependenceLowGrayLevelEmphasis	0.821507
**Original**	gldm	SmallDependenceEmphasis	0.821447
**Original**	gldm	SmallDependenceHighGrayLevelEmphasis	0.821447
**Original**	gldm	SmallDependenceLowGrayLevelEmphasis	0.821447
**w-LLL**	gldm	SmallDependenceEmphasis	0.821447
**w-LLL**	gldm	SmallDependenceHighGrayLevelEmphasis	0.821447
**w-LLL**	gldm	SmallDependenceLowGrayLevelEmphasis	0.821447
**w-LHH**	glrlm	RunLengthNonUniformityNormalized	0.821444
**Original**	shape	SurfaceVolumeRatio	0.821294
**w-HHL**	glcm	Idmn	0.821284
**w-HHL**	glcm	Idm	0.821284
**w-HHL**	glcm	Id	0.821284
**w-HHL**	glcm	Contrast	0.821284
**w-HHL**	glcm	InverseVariance	0.821284
**w-HHL**	glcm	DifferenceAverage	0.821284
**w-HHL**	glcm	Idn	0.821284
**Original**	shape	MajorAxis	0.821264
**w-LLH**	ngtdm	Complexity	0.82067
**w-HLH**	gldm	LargeDependenceLowGrayLevelEmphasis	0.820027
**Original**	glszm	ZonePercentage	0.819692
**w-LLL**	glszm	ZonePercentage	0.819692
**w-HHL**	glcm	Correlation	0.818974
**w-HHL**	glcm	Imc2	0.818486
**w-LHH**	glcm	ClusterTendency	0.816773
**w-LHL**	glcm	SumEntropy	0.815143
**Original**	shape	Maximum3DDiameter	0.814842
**Original**	shape	MinorAxis	0.814273
**w-HLL**	gldm	SmallDependenceLowGrayLevelEmphasis	0.814158

**w-HLH**	ngtdm	Strength	0.813711
**w-HLH**	gldm	SmallDependenceEmphasis	0.813658
**w-LLH**	glcm	Autocorrelation	0.81305
**w-LHL**	glrlm	RunLengthNonUniformity	0.813027
**w-LLH**	gldm	LowGrayLevelEmphasis	0.812895
**w-LLH**	gldm	HighGrayLevelEmphasis	0.812895
**w-HHH**	glszm	ZonePercentage	0.811293
**w-HLH**	glszm	LargeAreaEmphasis	0.810777
**Original**	glrlm	RunLengthNonUniformity	0.810725
**w-LLL**	glrlm	RunLengthNonUniformity	0.810725
**w-LHL**	glrlm	ShortRunHighGrayLevelEmphasis	0.810383
**w-HLH**	ngtdm	Coarseness	0.809553
**w-HHH**	firstorder	MeanAbsoluteDeviation	0.809518
**w-HHL**	glcm	Autocorrelation	0.809308
**w-HLH**	glszm	ZonePercentage	0.809153
**w-HLH**	glszm	ZoneVariance	0.809091
**w-LHH**	glrlm	ShortRunHighGrayLevelEmphasis	0.809031
**w-LLH**	glcm	SumSquares	0.808747
**w-LHH**	glcm	DifferenceVariance	0.808458
**w-HLL**	glszm	ZoneEntropy	0.807913
**w-HHH**	gldm	SmallDependenceEmphasis	0.807694
**w-HHL**	gldm	SmallDependenceHighGrayLevelEmphasis	0.807293
**Original**	shape	Maximum2DDiameterSlice	0.806618
**w-LLH**	gldm	SmallDependenceLowGrayLevelEmphasis	0.80592
**w-HHL**	glcm	JointAverage	0.805518
**w-HHL**	glcm	SumAverage	0.805518
**w-HHH**	gldm	SmallDependenceHighGrayLevelEmphasis	0.805319
**w-HHL**	gldm	LowGrayLevelEmphasis	0.80511
**w-HHL**	gldm	HighGrayLevelEmphasis	0.80511
**w-LHL**	glcm	JointAverage	0.804416
**w-LHL**	glcm	SumAverage	0.804416
**w-HHL**	gldm	SmallDependenceEmphasis	0.802408
**w-HHH**	ngtdm	Strength	0.80214
**w-HHH**	ngtdm	Coarseness	0.801705
**w-LHL**	firstorder	Skewness	0.801386
**w-HLL**	firstorder	Skewness	0.801142
**w-HHL**	glszm	ZoneEntropy	0.801005
**w-LHH**	glcm	DifferenceEntropy	0.800239
**w-HLL**	glcm	Imc1	0.799886
**w-LHL**	glcm	SumSquares	0.799621
**w-HHL**	glcm	MaximumProbability	0.799529
**w-HHH**	gldm	SmallDependenceLowGrayLevelEmphasis	0.79853
**w-LHH**	glcm	Imc1	0.79837
**w-LLH**	gldm	GrayLevelVariance	0.797732
**w-LLH**	firstorder	Uniformity	0.797732
**w-LHL**	glcm	Autocorrelation	0.797151

**w-HHH**	firstorder	Variance	0.797117
**w-HHL**	glszm	GrayLevelNonUniformity	0.796602
**w-HLH**	glszm	LargeAreaHighGrayLevelEmphasis	0.796276
**w-LHL**	glrlm	ShortRunLowGrayLevelEmphasis	0.796044
**w-HLH**	ngtdm	Contrast	0.79427
**w-HLH**	glcm	Autocorrelation	0.792616
**w-HHL**	glcm	ClusterProminence	0.791834
**w-LHH**	gldm	SmallDependenceHighGrayLevelEmphasis	0.79145
**w-LHH**	glszm	GrayLevelNonUniformity	0.791221
**w-HLH**	glcm	Correlation	0.791144
**w-LLH**	glcm	Imc1	0.790917
**w-LHH**	glcm	JointEntropy	0.790779
**w-HLL**	glrlm	GrayLevelNonUniformity	0.790742
**w-LHL**	gldm	LowGrayLevelEmphasis	0.790365
**w-LHL**	gldm	HighGrayLevelEmphasis	0.790365
**w-LLH**	firstorder	Entropy	0.790117
**w-HHL**	glrlm	RunLengthNonUniformity	0.789508
**w-HLL**	glszm	SizeZoneNonUniformityNormalized	0.789473
**w-HLH**	glcm	SumAverage	0.78932
**w-HLH**	glcm	JointAverage	0.78932
**w-HLH**	gldm	LowGrayLevelEmphasis	0.789033
**w-HLH**	gldm	HighGrayLevelEmphasis	0.789033
**w-LHH**	gldm	SmallDependenceEmphasis	0.788533
**w-HHL**	gldm	SmallDependenceLowGrayLevelEmphasis	0.787883
**w-HLH**	glcm	ClusterProminence	0.787145
**w-HHH**	glszm	GrayLevelNonUniformity	0.785935
**w-LHH**	glcm	JointEnergy	0.784767
**w-HLL**	glcm	MaximumProbability	0.784637
**w-LHL**	gldm	LargeDependenceHighGrayLevelEmphasis	0.783707
**w-LHL**	glszm	GrayLevelNonUniformityNormalized	0.78366
**w-LHL**	glszm	GrayLevelVariance	0.78366
**w-LHL**	gldm	GrayLevelVariance	0.783113
**w-LHL**	firstorder	Uniformity	0.783113
**w-LLH**	glrlm	ShortRunHighGrayLevelEmphasis	0.782743
**w-HHH**	firstorder	90Percentile	0.782394
**w-HLH**	glcm	MaximumProbability	0.782272
**w-HLH**	firstorder	Skewness	0.782053
**w-LLH**	firstorder	Skewness	0.780852
**w-HHH**	glszm	ZoneEntropy	0.780788
**w-HLL**	ngtdm	Contrast	0.780491
**w-HLH**	gldm	SmallDependenceLowGrayLevelEmphasis	0.779963
**w-HLL**	glrlm	LowGrayLevelRunEmphasis	0.779844
**w-HLL**	glrlm	HighGrayLevelRunEmphasis	0.779844
**w-HLH**	glrlm	RunLengthNonUniformity	0.77908
**w-HHL**	glszm	ZonePercentage	0.778268
**w-HLH**	glcm	JointEntropy	0.77783

**w-LHH**	glszm	ZoneEntropy	0.777608
**w-LHL**	glcm	ClusterTendency	0.776645
**w-HHL**	glcm	JointEntropy	0.775876
**w-LHH**	glszm	ZonePercentage	0.775614
**w-LHL**	firstorder	Entropy	0.775026
**w-LLH**	glszm	LargeAreaHighGrayLevelEmphasis	0.774882
**w-HLL**	ngtdm	Complexity	0.774738
**w-HLH**	glcm	Imc2	0.772963
**w-LHH**	glcm	SumEntropy	0.772923
**w-LHH**	gldm	SmallDependenceLowGrayLevelEmphasis	0.772647
**w-LLH**	glcm	ClusterTendency	0.772041
**w-LHH**	firstorder	Variance	0.771687
**w-HHH**	ngtdm	Contrast	0.768838
**w-HHL**	glcm	ClusterTendency	0.768756
**w-HLH**	ngtdm	Complexity	0.768011
**w-HHH**	ngtdm	Complexity	0.766906
**w-LHH**	glcm	Autocorrelation	0.765845
**w-HLL**	glcm	JointEntropy	0.765456
**w-LHL**	glcm	Imc1	0.76417
**w-LHH**	glcm	ClusterShade	0.761563
**w-HHL**	glcm	JointEnergy	0.758679
**w-HLL**	glcm	JointEnergy	0.757236
**w-LHL**	glszm	ZonePercentage	0.756245
**w-LHH**	ngtdm	Contrast	0.754838
**w-LHH**	glcm	JointAverage	0.753014
**w-LHH**	glcm	SumAverage	0.753014
**w-HLH**	glcm	JointEnergy	0.752966
**w-LHH**	ngtdm	Complexity	0.752239
**w-HLL**	glszm	SizeZoneNonUniformity	0.751843
**w-LLH**	gldm	SmallDependenceEmphasis	0.749145
**w-HLL**	glcm	SumEntropy	0.747423
**w-HLL**	glcm	Correlation	0.747265
**w-HHH**	firstorder	RobustMeanAbsoluteDeviation	0.747005
**w-LLH**	gldm	LargeDependenceHighGrayLevelEmphasis	0.746301
**w-LLH**	firstorder	Kurtosis	0.744151
**w-HHL**	glcm	SumEntropy	0.743053
**w-HHH**	firstorder	InterquartileRange	0.742896
**w-LHH**	gldm	LowGrayLevelEmphasis	0.740536
**w-LHH**	gldm	HighGrayLevelEmphasis	0.740536
**w-HLL**	glcm	SumSquares	0.73974
**w-LLH**	glszm	GrayLevelNonUniformityNormalized	0.737738
**w-LLH**	glszm	GrayLevelVariance	0.737738
**w-LLH**	ngtdm	Strength	0.737117
**w-LHL**	glszm	HighGrayLevelZoneEmphasis	0.736978
**w-LHL**	glszm	LowGrayLevelZoneEmphasis	0.736978
**Original**	glrlm	ShortRunLowGrayLevelEmphasis	0.736339

**Original**	glrlm	ShortRunHighGrayLevelEmphasis	0.736339
**Original**	glrlm	ShortRunEmphasis	0.736339
**w-LLL**	glrlm	ShortRunLowGrayLevelEmphasis	0.736339
**w-LLL**	glrlm	ShortRunHighGrayLevelEmphasis	0.736339
**w-LLL**	glrlm	ShortRunEmphasis	0.736339
**w-HLL**	glcm	ClusterTendency	0.735673
**w-HLL**	firstorder	Uniformity	0.733763
**w-HLL**	gldm	GrayLevelVariance	0.733763
**w-HLH**	glcm	SumEntropy	0.732203
**w-LLH**	ngtdm	Coarseness	0.731848
**w-LHH**	firstorder	Median	0.73155
**w-LHH**	firstorder	Skewness	0.729482
**w-HLH**	glszm	HighGrayLevelZoneEmphasis	0.728097
**w-HLH**	glszm	LowGrayLevelZoneEmphasis	0.728097
**w-LLH**	glcm	ClusterShade	0.727123
**w-LLH**	glszm	ZoneEntropy	0.723899
**w-HLL**	glrlm	RunLengthNonUniformity	0.721477
**w-HLL**	glszm	GrayLevelNonUniformityNormalized	0.721093
**w-HLL**	glszm	GrayLevelVariance	0.721093
**w-HLL**	firstorder	Kurtosis	0.719915
**w-HHL**	ngtdm	Complexity	0.719114
**w-HLL**	glszm	SmallAreaLowGrayLevelEmphasis	0.717785
**w-HLL**	firstorder	Entropy	0.717526
**w-HLL**	glszm	GrayLevelNonUniformity	0.712836
**w-LLH**	gldm	SmallDependenceHighGrayLevelEmphasis	0.711468
**w-HHL**	glcm	SumSquares	0.710312
**w-LLH**	glcm	Correlation	0.710066
**w-LHL**	glcm	Imc2	0.703701
**w-HHL**	glrlm	LowGrayLevelRunEmphasis	0.701593
**w-HHL**	glrlm	HighGrayLevelRunEmphasis	0.701593
**w-LHH**	glszm	SmallAreaEmphasis	0.70063
**w-HHL**	firstorder	Uniformity	0.699182
**w-HHL**	gldm	GrayLevelVariance	0.699182
**w-LHL**	glcm	Correlation	0.696091
**w-HLH**	gldm	GrayLevelVariance	0.69323
**w-HLH**	firstorder	Uniformity	0.69323
**w-LLH**	glcm	Imc2	0.693026
**w-HHH**	firstorder	Skewness	0.692344
**w-HHH**	firstorder	Kurtosis	0.691229
**w-LHL**	ngtdm	Strength	0.689345
**w-HHH**	firstorder	10Percentile	0.68924
**w-HLH**	glcm	ClusterTendency	0.6876
**w-HLH**	glcm	SumSquares	0.686838
**w-HHL**	firstorder	Entropy	0.685318
**w-LHL**	ngtdm	Coarseness	0.684776
**w-LLH**	glszm	HighGrayLevelZoneEmphasis	0.6829

**w-LLH**	glszm	LowGrayLevelZoneEmphasis	0.6829
**w-HLH**	firstorder	Entropy	0.680889
**w-HHL**	ngtdm	Contrast	0.679164
**w-HLH**	glszm	SmallAreaHighGrayLevelEmphasis	0.671473
**w-LHH**	glszm	SizeZoneNonUniformityNormalized	0.670554
**w-HLL**	glcm	Imc2	0.66918
**w-HHL**	firstorder	Skewness	0.669062
**w-LLH**	glszm	ZonePercentage	0.667793
**w-LHL**	glszm	SizeZoneNonUniformityNormalized	0.666952
**w-HLH**	glszm	SizeZoneNonUniformityNormalized	0.662971
**w-HHL**	glszm	SmallAreaEmphasis	0.662923
**w-HHH**	glszm	SmallAreaEmphasis	0.662396
**w-HLH**	glszm	SmallAreaEmphasis	0.660353
**w-HHL**	glszm	LowGrayLevelZoneEmphasis	0.648221
**w-HHL**	glszm	HighGrayLevelZoneEmphasis	0.648221
**w-HHL**	ngtdm	Strength	0.646396
**w-LHH**	firstorder	Mean	0.643237
**w-LLH**	glszm	SmallAreaHighGrayLevelEmphasis	0.643157
**w-HLH**	glszm	GrayLevelNonUniformityNormalized	0.636137
**w-HLH**	glszm	GrayLevelVariance	0.636137
**w-HHL**	glszm	SmallAreaHighGrayLevelEmphasis	0.6323
**w-HHH**	glszm	SizeZoneNonUniformityNormalized	0.630856
**w-HHH**	glcm	ClusterShade	0.6297
**w-HHH**	glcm	Autocorrelation	0.62721
**w-HLL**	glszm	SmallAreaHighGrayLevelEmphasis	0.626413
**w-LHH**	glszm	SmallAreaHighGrayLevelEmphasis	0.626379
**w-LHH**	glcm	SumSquares	0.626154
**w-HLL**	glszm	SmallAreaEmphasis	0.623597
**w-HHL**	ngtdm	Coarseness	0.620216
**w-LHH**	firstorder	Uniformity	0.615966
**w-LHH**	gldm	GrayLevelVariance	0.615966
**w-LHL**	glcm	ClusterShade	0.614305
**w-LHH**	firstorder	Entropy	0.613581
**w-HHH**	glszm	SmallAreaLowGrayLevelEmphasis	0.611596
**w-HLH**	glszm	SmallAreaLowGrayLevelEmphasis	0.607488
**w-LLH**	glcm	ClusterProminence	0.602745
**w-HHL**	firstorder	Kurtosis	0.602395
**w-HLL**	glrlm	GrayLevelVariance	0.599762
**w-HLL**	glrlm	GrayLevelNonUniformityNormalized	0.599762
**w-HLH**	glrlm	HighGrayLevelRunEmphasis	0.598872
**w-HLH**	glrlm	LowGrayLevelRunEmphasis	0.598872
**w-HHH**	glcm	SumAverage	0.598276
**w-HHH**	glcm	JointAverage	0.598276
**w-HHL**	glszm	SizeZoneNonUniformityNormalized	0.597354
**w-HHH**	gldm	LowGrayLevelEmphasis	0.597336
**w-HHH**	gldm	HighGrayLevelEmphasis	0.597336

**w-HHH**	firstorder	Mean	0.59592
**w-LLH**	glszm	SmallAreaEmphasis	0.595339
**w-LHH**	glszm	SmallAreaLowGrayLevelEmphasis	0.592657
**w-LHL**	glszm	SmallAreaHighGrayLevelEmphasis	0.592202
**w-LHH**	firstorder	Kurtosis	0.581283
**w-LHL**	glszm	SmallAreaEmphasis	0.581281
**w-LHL**	glrlm	LowGrayLevelRunEmphasis	0.580356
**w-LHL**	glrlm	HighGrayLevelRunEmphasis	0.580356
**w-HHH**	glszm	SmallAreaHighGrayLevelEmphasis	0.575205
**w-LLH**	glrlm	HighGrayLevelRunEmphasis	0.574737
**w-LLH**	glrlm	LowGrayLevelRunEmphasis	0.574737
**w-LHL**	glcm	ClusterProminence	0.574376
**w-HHL**	glszm	SmallAreaLowGrayLevelEmphasis	0.561765
**w-LHH**	glrlm	LowGrayLevelRunEmphasis	0.560976
**w-LHH**	glrlm	HighGrayLevelRunEmphasis	0.560976
**w-HHL**	glszm	GrayLevelNonUniformityNormalized	0.559339
**w-HHL**	glszm	GrayLevelVariance	0.559339
**w-HLL**	ngtdm	Coarseness	0.552944
**w-LHL**	glszm	SmallAreaLowGrayLevelEmphasis	0.544309
**w-HLH**	firstorder	Kurtosis	0.536428
**w-HLL**	ngtdm	Busyness	0.524586
**w-HLL**	glcm	ClusterProminence	0.514432
**w-HLH**	glrlm	GrayLevelVariance	0.509644
**w-HLH**	glrlm	GrayLevelNonUniformityNormalized	0.509644
**w-HHH**	glrlm	HighGrayLevelRunEmphasis	0.497327
**w-HHH**	glrlm	LowGrayLevelRunEmphasis	0.497327
**w-LLH**	glszm	SmallAreaLowGrayLevelEmphasis	0.490605
**w-LHH**	glrlm	GrayLevelNonUniformityNormalized	0.489541
**w-LHH**	glrlm	GrayLevelVariance	0.489541
**w-HHL**	glrlm	GrayLevelNonUniformityNormalized	0.461165
**w-HHL**	glrlm	GrayLevelVariance	0.461165
**Original**	glszm	SmallAreaHighGrayLevelEmphasis	0.455647
**Original**	glszm	SmallAreaEmphasis	0.455647
**Original**	glszm	SmallAreaLowGrayLevelEmphasis	0.455647
**w-LLL**	glszm	SmallAreaHighGrayLevelEmphasis	0.455647
**w-LLL**	glszm	SmallAreaEmphasis	0.455647
**w-LLL**	glszm	SmallAreaLowGrayLevelEmphasis	0.455647
**w-LLH**	glrlm	GrayLevelVariance	0.454859
**w-LLH**	glrlm	GrayLevelNonUniformityNormalized	0.454859
**w-LHL**	glrlm	GrayLevelNonUniformityNormalized	0.426658
**w-LHL**	glrlm	GrayLevelVariance	0.426658
**w-HHH**	glcm	SumSquares	0.423653
**w-HHH**	firstorder	Uniformity	0.396599
**w-HHH**	gldm	GrayLevelVariance	0.396599
**w-LLH**	glszm	SizeZoneNonUniformityNormalized	0.396525
**w-HHH**	firstorder	Entropy	0.393627

**w-HHH**	glszm	GrayLevelNonUniformityNormalized	0.371436
**w-HHH**	glszm	GrayLevelVariance	0.371436
**w-HHH**	firstorder	Median	0.356719
**w-HHH**	glrlm	GrayLevelNonUniformityNormalized	0.321387
**w-HHH**	glrlm	GrayLevelVariance	0.321387
**w-LHH**	glszm	GrayLevelNonUniformityNormalized	0.304709
**w-LHH**	glszm	GrayLevelVariance	0.304709
**w-HHL**	ngtdm	Busyness	0.296658
**w-HLL**	ngtdm	Strength	0.195783
**w-HHH**	glszm	HighGrayLevelZoneEmphasis	0.080397
**w-HHH**	glszm	LowGrayLevelZoneEmphasis	0.080397
**w-LHH**	glszm	HighGrayLevelZoneEmphasis	0.072932
**w-LHH**	glszm	LowGrayLevelZoneEmphasis	0.072932
**w-LHL**	ngtdm	Busyness	0.069914
**w-LLH**	ngtdm	Busyness	0.039685
**w-HLH**	ngtdm	Busyness	0.00489

Supplementary Table 3. The ICC values of radiomics features extracted in the current study after combat NPNE harmonization.

Set	Type	Name	value
**Original**	firstorder	Maximum	0.999767
**Original**	firstorder	Range	0.99913
**w-LLL**	firstorder	Maximum	0.998137
**Original**	firstorder	90Percentile	0.998063
**w-LLL**	firstorder	Range	0.997653
**w-LLL**	firstorder	90Percentile	0.997003
**Original**	firstorder	Energy	0.996043
**Original**	firstorder	TotalEnergy	0.996043
**Original**	firstorder	RootMeanSquared	0.995983
**w-LLL**	firstorder	Energy	0.99573
**w-LLL**	firstorder	TotalEnergy	0.995729
**w-LLL**	firstorder	RootMeanSquared	0.995119
**Original**	firstorder	MeanAbsoluteDeviation	0.994671
**Original**	firstorder	Mean	0.994524
**w-LLL**	firstorder	MeanAbsoluteDeviation	0.994406
**w-LLL**	firstorder	Mean	0.993949
**w-LLL**	firstorder	RobustMeanAbsoluteDeviation	0.992735
**Original**	firstorder	Median	0.992532
**w-LLL**	firstorder	Median	0.99247
**Original**	firstorder	RobustMeanAbsoluteDeviation	0.992055
**w-LLL**	firstorder	InterquartileRange	0.99205
**Original**	firstorder	InterquartileRange	0.990958
**w-LLL**	firstorder	Variance	0.990824
**Original**	firstorder	Variance	0.990579
**w-LHL**	firstorder	InterquartileRange	0.989495
**w-LHL**	firstorder	MeanAbsoluteDeviation	0.989308
**w-LHL**	firstorder	RobustMeanAbsoluteDeviation	0.989265
**w-LLH**	firstorder	RobustMeanAbsoluteDeviation	0.987587
**w-LHL**	firstorder	Range	0.987256
**w-LHL**	firstorder	Minimum	0.987254
**w-LHL**	firstorder	10Percentile	0.986969
**w-LLH**	firstorder	10Percentile	0.986866
**w-LLH**	firstorder	InterquartileRange	0.986612
**w-LHL**	firstorder	Maximum	0.986366
**w-HHL**	firstorder	Energy	0.986249
**w-HHL**	firstorder	TotalEnergy	0.986243
**w-HLL**	firstorder	Variance	0.985437
**w-LLL**	firstorder	10Percentile	0.984326
**w-LHL**	firstorder	RootMeanSquared	0.984041
**w-LLH**	firstorder	MeanAbsoluteDeviation	0.983958
**Original**	firstorder	10Percentile	0.983919
**w-HLL**	firstorder	Energy	0.983753

**w-HLL**	firstorder	TotalEnergy	0.983753
**w-HLL**	firstorder	Range	0.983539
**w-HLL**	firstorder	Maximum	0.982278
**w-HLL**	firstorder	RootMeanSquared	0.981025
**w-HLL**	firstorder	MeanAbsoluteDeviation	0.980967
**w-LLH**	firstorder	Range	0.980823
**w-HLL**	firstorder	Minimum	0.980694
**w-LLH**	firstorder	Minimum	0.980519
**w-HLL**	firstorder	RobustMeanAbsoluteDeviation	0.980082
**w-LLH**	firstorder	Maximum	0.979524
**w-HLL**	firstorder	InterquartileRange	0.979347
**w-LHL**	firstorder	Energy	0.97875
**w-LHL**	firstorder	TotalEnergy	0.978739
**w-LLH**	glrlm	LongRunLowGrayLevelEmphasis	0.978546
**w-HLL**	firstorder	90Percentile	0.978499
**Original**	glszm	LargeAreaEmphasis	0.97817
**Original**	glszm	LargeAreaLowGrayLevelEmphasis	0.97817
**Original**	glszm	LargeAreaHighGrayLevelEmphasis	0.97817
**w-LLL**	glszm	LargeAreaEmphasis	0.97817
**w-LLL**	glszm	LargeAreaLowGrayLevelEmphasis	0.97817
**w-LLL**	glszm	LargeAreaHighGrayLevelEmphasis	0.97817
**w-HHL**	firstorder	RobustMeanAbsoluteDeviation	0.977408
**w-HHL**	firstorder	InterquartileRange	0.977356
**w-LLH**	glrlm	RunVariance	0.977271
**w-HHL**	firstorder	Minimum	0.975749
**w-HHL**	firstorder	Variance	0.975651
**w-LHL**	firstorder	Variance	0.975625
**w-HHL**	firstorder	Range	0.975625
**w-HHL**	firstorder	Maximum	0.975181
**w-LLH**	firstorder	RootMeanSquared	0.974723
**w-LHL**	glrlm	LongRunLowGrayLevelEmphasis	0.974682
**w-LLH**	glrlm	LongRunEmphasis	0.973835
**w-HHL**	firstorder	90Percentile	0.971811
**w-HHL**	firstorder	MeanAbsoluteDeviation	0.971243
**w-LHL**	firstorder	Mean	0.970957
**w-LHH**	firstorder	InterquartileRange	0.969797
**w-HHL**	firstorder	RootMeanSquared	0.969763
**w-HHL**	firstorder	10Percentile	0.969463
**w-HLH**	firstorder	Range	0.969324
**w-LHL**	gldm	DependenceNonUniformityNormalized	0.969299
**w-HLH**	firstorder	Maximum	0.969285
**w-LLH**	firstorder	Mean	0.969201
**w-LHH**	firstorder	RobustMeanAbsoluteDeviation	0.968852
**w-LHL**	glrlm	RunVariance	0.967853
**w-HLH**	firstorder	Minimum	0.967215
**w-HLL**	glszm	LargeAreaLowGrayLevelEmphasis	0.967076

**w-LHL**	glrlm	LongRunEmphasis	0.964957
**w-HLL**	glszm	ZoneVariance	0.964892
**w-HLH**	firstorder	RobustMeanAbsoluteDeviation	0.964438
**w-HLH**	firstorder	MeanAbsoluteDeviation	0.964216
**w-LHL**	gldm	LargeDependenceEmphasis	0.964213
**w-HLH**	firstorder	InterquartileRange	0.964076
**w-LHL**	glrlm	RunPercentage	0.962133
**w-LLH**	firstorder	Energy	0.962112
**w-LLH**	firstorder	TotalEnergy	0.962082
**w-LHH**	firstorder	90Percentile	0.962022
**w-HLH**	firstorder	Energy	0.96125
**w-HLH**	firstorder	TotalEnergy	0.961236
**w-HLH**	firstorder	10Percentile	0.961133
**w-LHL**	firstorder	Median	0.961101
**w-HLL**	glszm	LargeAreaEmphasis	0.960825
**w-HLH**	firstorder	RootMeanSquared	0.96016
**Original**	firstorder	Minimum	0.959319
**w-LHL**	gldm	DependenceEntropy	0.958081
**w-HLL**	firstorder	10Percentile	0.957509
**w-HHH**	glrlm	LongRunHighGrayLevelEmphasis	0.956869
**w-LLH**	gldm	LargeDependenceEmphasis	0.956668
**w-HLH**	glrlm	LongRunHighGrayLevelEmphasis	0.956614
**w-LLH**	gldm	DependenceNonUniformityNormalized	0.956298
**w-HLH**	glrlm	LongRunEmphasis	0.956159
**w-LLH**	firstorder	Median	0.955983
**w-HLH**	gldm	DependenceNonUniformity	0.955965
**w-HHH**	glrlm	LongRunEmphasis	0.955826
**w-HLH**	glrlm	RunVariance	0.955809
**w-LHL**	gldm	DependenceNonUniformity	0.955745
**w-LLH**	firstorder	90Percentile	0.955383
**w-HHH**	glszm	LargeAreaHighGrayLevelEmphasis	0.954981
**w-LLL**	firstorder	Minimum	0.954704
**w-HHH**	glrlm	RunVariance	0.954631
**w-LLH**	glrlm	RunPercentage	0.954494
**w-HHH**	gldm	DependenceNonUniformityNormalized	0.954192
**w-HHH**	glszm	ZoneVariance	0.95362
**w-HLH**	firstorder	90Percentile	0.953549
**w-HHH**	glszm	LargeAreaEmphasis	0.953432
**w-HHH**	glrlm	LongRunLowGrayLevelEmphasis	0.95338
**w-LLH**	gldm	DependenceEntropy	0.953254
**w-HHL**	gldm	DependenceNonUniformity	0.953162
**w-HLL**	glrlm	LongRunLowGrayLevelEmphasis	0.952791
**w-HHH**	glszm	LargeAreaLowGrayLevelEmphasis	0.951739
**w-LLH**	gldm	LargeDependenceLowGrayLevelEmphasis	0.951371
**w-LHH**	glrlm	RunVariance	0.951319
**w-HLL**	glrlm	RunLengthNonUniformityNormalized	0.951216

**w-LHH**	firstorder	10Percentile	0.951205
**w-LHH**	firstorder	MeanAbsoluteDeviation	0.95115
**w-HLL**	glszm	LargeAreaHighGrayLevelEmphasis	0.95096
**w-LHL**	ngtdm	Contrast	0.950889
**w-LHH**	glszm	LargeAreaLowGrayLevelEmphasis	0.95081
**w-HHH**	firstorder	InterquartileRange	0.950641
**w-LHH**	gldm	DependenceNonUniformityNormalized	0.950582
**w-LHH**	glszm	ZoneVariance	0.95053
**w-LLH**	gldm	DependenceNonUniformity	0.950287
**w-LHH**	glszm	LargeAreaEmphasis	0.950101
**w-LLH**	firstorder	Variance	0.949705
**w-LHH**	glrlm	LongRunHighGrayLevelEmphasis	0.949698
**w-LHH**	glszm	LargeAreaHighGrayLevelEmphasis	0.949326
**w-LHH**	glrlm	LongRunEmphasis	0.949178
**w-HLH**	glrlm	LongRunLowGrayLevelEmphasis	0.948317
**w-LHL**	gldm	LargeDependenceLowGrayLevelEmphasis	0.947561
**w-LHH**	glrlm	RunLengthNonUniformity	0.947421
**w-HHH**	firstorder	RobustMeanAbsoluteDeviation	0.946836
**w-LHH**	glrlm	LongRunLowGrayLevelEmphasis	0.946239
**w-HHH**	gldm	DependenceEntropy	0.94616
**w-LHL**	glrlm	RunEntropy	0.946008
**w-HLL**	glrlm	RunEntropy	0.945961
**w-HHH**	gldm	DependenceNonUniformity	0.945918
**w-HLL**	glrlm	RunPercentage	0.94571
**w-HLL**	gldm	LargeDependenceEmphasis	0.944586
**w-LHH**	ngtdm	Busyness	0.944522
**w-HHH**	glrlm	RunLengthNonUniformity	0.944411
**w-HLH**	glszm	SizeZoneNonUniformity	0.943417
**w-LHL**	glszm	LargeAreaLowGrayLevelEmphasis	0.943258
**w-HLL**	firstorder	Median	0.943044
**w-HLL**	gldm	DependenceVariance	0.943018
**w-LLH**	gldm	GrayLevelNonUniformity	0.942958
**w-LHH**	gldm	DependenceVariance	0.942906
**w-LHL**	ngtdm	Complexity	0.942176
**w-LHH**	gldm	DependenceNonUniformity	0.942119
**Original**	glrlm	RunEntropy	0.94205
**w-LLL**	glrlm	RunEntropy	0.94205
**w-LHL**	gldm	GrayLevelNonUniformity	0.941524
**w-HHH**	gldm	DependenceVariance	0.941409
**w-HHL**	glrlm	LongRunHighGrayLevelEmphasis	0.940815
**Original**	glrlm	LongRunEmphasis	0.940808
**Original**	glrlm	LongRunHighGrayLevelEmphasis	0.940808
**Original**	glrlm	LongRunLowGrayLevelEmphasis	0.940808
**w-LLL**	glrlm	LongRunEmphasis	0.940808
**w-LLL**	glrlm	LongRunHighGrayLevelEmphasis	0.940808
**w-LLL**	glrlm	LongRunLowGrayLevelEmphasis	0.940808

**w-HLL**	ngtdm	Contrast	0.940777
**w-HHL**	glrlm	RunVariance	0.940544
**w-HLL**	gldm	DependenceNonUniformity	0.940358
**w-HLH**	firstorder	Variance	0.940236
**w-HLH**	glrlm	RunEntropy	0.93954
**w-HLL**	ngtdm	Complexity	0.939202
**w-LHH**	glrlm	GrayLevelNonUniformity	0.93918
**Original**	gldm	LargeDependenceEmphasis	0.938633
**Original**	gldm	LargeDependenceLowGrayLevelEmphasis	0.938633
**Original**	gldm	LargeDependenceHighGrayLevelEmphasis	0.938633
**w-LLL**	gldm	LargeDependenceEmphasis	0.938633
**w-LLL**	gldm	LargeDependenceLowGrayLevelEmphasis	0.938633
**w-LLL**	gldm	LargeDependenceHighGrayLevelEmphasis	0.938633
**w-HHL**	glrlm	LongRunEmphasis	0.937906
**w-HLL**	firstorder	Mean	0.937872
**Original**	gldm	DependenceNonUniformity	0.937811
**w-LLL**	gldm	DependenceNonUniformity	0.937811
**Original**	glszm	ZonePercentage	0.9378
**w-LLL**	glszm	ZonePercentage	0.9378
**Original**	glrlm	RunPercentage	0.937786
**w-LLL**	glrlm	RunPercentage	0.937786
**w-LHL**	glszm	ZoneVariance	0.937074
**w-HHH**	glrlm	GrayLevelNonUniformity	0.936923
**w-LLH**	ngtdm	Contrast	0.936636
**w-HLL**	glrlm	ShortRunEmphasis	0.93655
**w-HHL**	glszm	LargeAreaLowGrayLevelEmphasis	0.93608
**Original**	gldm	DependenceVariance	0.936034
**w-LLL**	gldm	DependenceVariance	0.936034
**w-HLL**	gldm	LargeDependenceLowGrayLevelEmphasis	0.935725
**w-LLH**	glrlm	LongRunHighGrayLevelEmphasis	0.93502
**w-HLH**	gldm	GrayLevelNonUniformity	0.934892
**Original**	gldm	DependenceNonUniformityNormalized	0.934607
**w-LLL**	gldm	DependenceNonUniformityNormalized	0.934607
**Original**	shape	LeastAxis	0.934534
**w-LHL**	glrlm	RunLengthNonUniformityNormalized	0.934447
**w-LLH**	glszm	SizeZoneNonUniformity	0.934248
**w-HHL**	glszm	ZoneVariance	0.93414
**w-HHH**	gldm	GrayLevelNonUniformity	0.934076
**w-HHL**	gldm	GrayLevelNonUniformity	0.934054
**Original**	gldm	GrayLevelNonUniformity	0.934044
**w-LLL**	gldm	GrayLevelNonUniformity	0.934044
**Original**	glrlm	RunVariance	0.933997
**w-LLL**	glrlm	RunVariance	0.933997
**w-LHH**	gldm	GrayLevelNonUniformity	0.93399
**Original**	shape	Volume	0.933704
**Original**	firstorder	Skewness	0.933693

**w-HHH**	ngtdm	Busyness	0.933588
**w-HHL**	glszm	LargeAreaEmphasis	0.933057
**w-LHH**	glszm	SizeZoneNonUniformity	0.932936
**w-LHL**	glszm	LargeAreaEmphasis	0.932919
**w-HLL**	gldm	GrayLevelNonUniformity	0.932741
**w-LHH**	firstorder	Energy	0.93264
**w-LHH**	firstorder	TotalEnergy	0.932561
**w-LLH**	glrlm	RunEntropy	0.932245
**w-HLH**	glrlm	ShortRunEmphasis	0.932187
**w-HHL**	glrlm	RunEntropy	0.932168
**w-HLL**	gldm	SmallDependenceEmphasis	0.931937
**w-LHH**	gldm	DependenceEntropy	0.931916
**Original**	shape	SurfaceVolumeRatio	0.931718
**w-HLL**	glrlm	LongRunEmphasis	0.931537
**w-LLH**	ngtdm	Complexity	0.93141
**w-LHL**	glcm	DifferenceVariance	0.931033
**w-LLH**	glcm	DifferenceVariance	0.930919
**w-LHL**	glszm	SizeZoneNonUniformity	0.930364
**w-LLH**	glcm	DifferenceEntropy	0.930358
**Original**	gldm	SmallDependenceEmphasis	0.930311
**Original**	gldm	SmallDependenceHighGrayLevelEmphasis	0.930311
**Original**	gldm	SmallDependenceLowGrayLevelEmphasis	0.930311
**w-LLL**	gldm	SmallDependenceEmphasis	0.930311
**w-LLL**	gldm	SmallDependenceHighGrayLevelEmphasis	0.930311
**w-LLL**	gldm	SmallDependenceLowGrayLevelEmphasis	0.930311
**w-HLH**	glszm	GrayLevelNonUniformity	0.930249
**w-HHH**	firstorder	90Percentile	0.929962
**w-LHL**	glcm	Idm	0.929937
**w-LHL**	glcm	Id	0.929937
**w-LHL**	glcm	Idmn	0.929937
**w-LHL**	glcm	Contrast	0.929937
**w-LHL**	glcm	InverseVariance	0.929937
**w-LHL**	glcm	DifferenceAverage	0.929937
**w-LHL**	glcm	Idn	0.929937
**w-LHH**	firstorder	RootMeanSquared	0.929851
**w-LLH**	glszm	LargeAreaLowGrayLevelEmphasis	0.929797
**w-HHH**	firstorder	Energy	0.929074
**w-HHL**	glszm	LargeAreaHighGrayLevelEmphasis	0.92903
**w-HHH**	firstorder	TotalEnergy	0.928968
**w-HHL**	glrlm	ShortRunEmphasis	0.928879
**w-HLL**	gldm	DependenceNonUniformityNormalized	0.928775
**w-HHL**	glrlm	LongRunLowGrayLevelEmphasis	0.928579
**w-HLL**	gldm	SmallDependenceHighGrayLevelEmphasis	0.928373
**w-LLH**	glcm	Idmn	0.928176
**w-LLH**	glcm	Idm	0.928176
**w-LLH**	glcm	Id	0.928176

**w-LLH**	glcm	Contrast	0.928176
**w-LLH**	glcm	InverseVariance	0.928176
**w-LLH**	glcm	DifferenceAverage	0.928176
**w-LLH**	glcm	Idn	0.928176
**Original**	gldm	DependenceEntropy	0.927705
**w-LLL**	gldm	DependenceEntropy	0.927705
**w-LHL**	glcm	DifferenceEntropy	0.927427
**w-HLL**	glszm	ZonePercentage	0.92703
**w-HLH**	gldm	DependenceNonUniformityNormalized	0.926705
**w-HHH**	firstorder	Range	0.926341
**w-HHH**	firstorder	Minimum	0.926207
**w-HHH**	glrlm	RunEntropy	0.925313
**w-HLL**	glrlm	RunVariance	0.924744
**w-HHH**	firstorder	MeanAbsoluteDeviation	0.924699
**w-HHH**	firstorder	Maximum	0.924395
**w-HLH**	ngtdm	Complexity	0.923026
**w-HLH**	glrlm	ShortRunLowGrayLevelEmphasis	0.922838
**w-HHL**	glszm	SizeZoneNonUniformity	0.922639
**w-LLH**	glrlm	GrayLevelNonUniformity	0.92252
**w-HLH**	glrlm	RunLengthNonUniformityNormalized	0.922418
**w-LLH**	glszm	GrayLevelNonUniformity	0.922282
**Original**	glrlm	RunLengthNonUniformityNormalized	0.922116
**w-LLL**	glrlm	RunLengthNonUniformityNormalized	0.922116
**w-HLH**	ngtdm	Contrast	0.921847
**w-HLH**	glrlm	RunPercentage	0.92168
**w-LHL**	glrlm	LongRunHighGrayLevelEmphasis	0.921272
**w-HHL**	glrlm	ShortRunLowGrayLevelEmphasis	0.920904
**w-LHH**	firstorder	Range	0.920856
**w-LLH**	glszm	ZoneVariance	0.920754
**w-HHH**	firstorder	10Percentile	0.920657
**w-LHH**	ngtdm	Contrast	0.920494
**w-LHH**	firstorder	Minimum	0.920476
**w-LHH**	ngtdm	Complexity	0.92027
**w-HHL**	glrlm	ShortRunHighGrayLevelEmphasis	0.919622
**w-HHH**	ngtdm	Contrast	0.919595
**w-HHH**	ngtdm	Coarseness	0.919384
**w-HHH**	ngtdm	Complexity	0.91938
**w-HHH**	ngtdm	Strength	0.919374
**Original**	shape	Flatness	0.918933
**w-LHL**	glrlm	GrayLevelNonUniformity	0.918567
**w-LLH**	glrlm	RunLengthNonUniformityNormalized	0.918415
**w-HHH**	firstorder	RootMeanSquared	0.918149
**w-HLH**	firstorder	Median	0.91795
**w-LLH**	glszm	LargeAreaEmphasis	0.917856
**w-HLH**	gldm	LargeDependenceEmphasis	0.917778
**w-LLL**	firstorder	Skewness	0.917579

**w-HLH**	glszm	LargeAreaLowGrayLevelEmphasis	0.917123
**w-LHL**	firstorder	90Percentile	0.916362
**w-HHL**	glrlm	RunLengthNonUniformityNormalized	0.916153
**w-LLH**	glcm	JointEntropy	0.916055
**w-LHH**	ngtdm	Strength	0.915905
**w-LHH**	ngtdm	Coarseness	0.915868
**w-HLH**	glrlm	ShortRunHighGrayLevelEmphasis	0.915846
**w-LLH**	glrlm	RunLengthNonUniformity	0.915584
**Original**	shape	Maximum2DDiameterColumn	0.914273
**w-HLH**	ngtdm	Strength	0.913913
**w-HHL**	gldm	SmallDependenceEmphasis	0.913864
**w-HLH**	ngtdm	Coarseness	0.913687
**w-HHL**	glrlm	RunPercentage	0.913507
**w-HHL**	glrlm	GrayLevelNonUniformity	0.912893
**w-LHL**	gldm	DependenceVariance	0.912779
**w-LHH**	firstorder	Maximum	0.912626
**w-HLH**	glszm	ZoneVariance	0.91196
**w-HLH**	glrlm	GrayLevelNonUniformity	0.911207
**w-LHL**	gldm	SmallDependenceEmphasis	0.910981
**w-HLH**	glszm	ZonePercentage	0.910974
**w-LHL**	glszm	LargeAreaHighGrayLevelEmphasis	0.910902
**w-LHL**	glcm	JointEntropy	0.910811
**w-HHL**	ngtdm	Complexity	0.910751
**w-HLH**	glszm	LargeAreaEmphasis	0.910443
**w-LLH**	glcm	JointEnergy	0.910303
**w-HHL**	gldm	DependenceNonUniformityNormalized	0.910182
**w-LHH**	glszm	ZonePercentage	0.910103
**w-HLL**	glrlm	LongRunHighGrayLevelEmphasis	0.910035
**w-HHL**	gldm	SmallDependenceHighGrayLevelEmphasis	0.909891
**w-LHL**	gldm	SmallDependenceLowGrayLevelEmphasis	0.909847
**Original**	glrlm	GrayLevelNonUniformity	0.909578
**w-LLL**	glrlm	GrayLevelNonUniformity	0.909578
**w-HHL**	gldm	LargeDependenceEmphasis	0.909252
**w-HHH**	glszm	ZonePercentage	0.909213
**Original**	shape	SurfaceArea	0.909203
**w-HHL**	gldm	SmallDependenceLowGrayLevelEmphasis	0.908962
**w-HHH**	gldm	LargeDependenceEmphasis	0.908863
**Original**	shape	Sphericity	0.908827
**w-HLH**	gldm	SmallDependenceEmphasis	0.908406
**w-HLH**	firstorder	Mean	0.908354
**w-HHL**	ngtdm	Strength	0.908021
**w-LHL**	glszm	GrayLevelNonUniformity	0.907476
**Original**	firstorder	Kurtosis	0.907413
**w-HLL**	gldm	SmallDependenceLowGrayLevelEmphasis	0.907353
**w-HHL**	ngtdm	Coarseness	0.907345
**w-LHH**	glrlm	RunEntropy	0.906459

**w-LLH**	glcm	SumEntropy	0.906448
**w-HHH**	glszm	SizeZoneNonUniformity	0.905831
**w-LHL**	gldm	SmallDependenceHighGrayLevelEmphasis	0.905753
**w-HLL**	glcm	ClusterShade	0.905558
**w-LHL**	glcm	JointEnergy	0.905487
**w-HHL**	firstorder	Mean	0.905341
**w-HHL**	gldm	LargeDependenceHighGrayLevelEmphasis	0.904204
**w-LHL**	glcm	SumEntropy	0.903688
**w-LLH**	glcm	MaximumProbability	0.903359
**w-HLL**	glrlm	ShortRunLowGrayLevelEmphasis	0.903337
**w-HHH**	gldm	LargeDependenceHighGrayLevelEmphasis	0.903241
**w-HHL**	glszm	ZonePercentage	0.903223
**w-HLL**	glcm	SumAverage	0.902576
**w-HLL**	glcm	JointAverage	0.902576
**w-HLH**	glszm	LargeAreaHighGrayLevelEmphasis	0.901994
**w-HHL**	ngtdm	Contrast	0.901986
**w-LHL**	glrlm	ShortRunEmphasis	0.9018
**w-HLL**	gldm	HighGrayLevelEmphasis	0.901664
**w-HLL**	gldm	LowGrayLevelEmphasis	0.901664
**w-HLL**	gldm	DependenceEntropy	0.901627
**w-LHL**	glrlm	RunLengthNonUniformity	0.901575
**w-HHH**	glrlm	RunPercentage	0.901572
**w-HLL**	glrlm	ShortRunHighGrayLevelEmphasis	0.901138
**w-HLH**	gldm	SmallDependenceLowGrayLevelEmphasis	0.900772
**w-HLL**	glcm	Autocorrelation	0.900455
**w-HHH**	gldm	SmallDependenceEmphasis	0.899896
**w-LLH**	ngtdm	Strength	0.899307
**w-LLH**	gldm	DependenceVariance	0.898845
**w-HLH**	gldm	SmallDependenceHighGrayLevelEmphasis	0.898726
**w-LHL**	firstorder	Kurtosis	0.898476
**w-HLL**	glrlm	GrayLevelNonUniformity	0.898195
**w-HHH**	firstorder	Variance	0.898023
**w-LHH**	firstorder	Variance	0.898008
**w-HHL**	firstorder	Median	0.897498
**w-HHH**	gldm	SmallDependenceHighGrayLevelEmphasis	0.897444
**w-LLH**	ngtdm	Coarseness	0.897315
**w-LHL**	glrlm	ShortRunLowGrayLevelEmphasis	0.895936
**w-HLH**	gldm	DependenceEntropy	0.895394
**w-LHL**	glcm	MaximumProbability	0.89468
**w-LLH**	gldm	SmallDependenceLowGrayLevelEmphasis	0.894346
**Original**	shape	Maximum2DDiameterRow	0.894215
**w-HHH**	gldm	SmallDependenceLowGrayLevelEmphasis	0.893823
**w-HLL**	gldm	LargeDependenceHighGrayLevelEmphasis	0.893192
**w-HHH**	glrlm	RunLengthNonUniformityNormalized	0.893188
**Original**	shape	MinorAxis	0.893086
**w-HLH**	gldm	LargeDependenceHighGrayLevelEmphasis	0.892963

**w-LLH**	glrlm	ShortRunLowGrayLevelEmphasis	0.892169
**w-LLH**	glszm	LargeAreaHighGrayLevelEmphasis	0.892058
**w-LHL**	glszm	ZonePercentage	0.891859
**w-HLL**	glcm	Contrast	0.891433
**w-HLL**	glcm	InverseVariance	0.891433
**w-HLL**	glcm	DifferenceAverage	0.891433
**w-HLL**	glcm	Idm	0.891433
**w-HLL**	glcm	Id	0.891433
**w-HLL**	glcm	Idmn	0.891433
**w-HLL**	glcm	Idn	0.891433
**Original**	shape	Maximum3DDiameter	0.891117
**w-LHL**	glcm	SumSquares	0.890149
**Original**	glrlm	RunLengthNonUniformity	0.889178
**w-LLL**	glrlm	RunLengthNonUniformity	0.889178
**w-HHH**	gldm	LargeDependenceLowGrayLevelEmphasis	0.889034
**w-LHH**	gldm	LargeDependenceEmphasis	0.88887
**w-LLH**	glcm	SumSquares	0.888611
**w-LHH**	gldm	SmallDependenceEmphasis	0.888205
**w-HLL**	glcm	DifferenceVariance	0.885506
**w-LLH**	glcm	SumAverage	0.885439
**w-LLH**	glcm	JointAverage	0.885439
**w-LHH**	gldm	SmallDependenceLowGrayLevelEmphasis	0.885361
**w-LHL**	firstorder	Uniformity	0.885035
**w-LHL**	gldm	GrayLevelVariance	0.885035
**w-LLH**	gldm	GrayLevelVariance	0.884841
**w-LLH**	firstorder	Uniformity	0.884841
**w-HLH**	glrlm	RunLengthNonUniformity	0.884785
**Original**	shape	Maximum2DDiameterSlice	0.884768
**w-HHH**	glcm	SumEntropy	0.883756
**w-LHH**	glrlm	RunPercentage	0.883699
**w-LLH**	gldm	SmallDependenceEmphasis	0.883599
**w-LLH**	firstorder	Entropy	0.88326
**Original**	shape	MajorAxis	0.883179
**w-LHL**	firstorder	Entropy	0.883
**w-LHL**	glcm	JointAverage	0.882608
**w-LHL**	glcm	SumAverage	0.882608
**w-LHH**	gldm	LargeDependenceLowGrayLevelEmphasis	0.882137
**w-LLH**	gldm	LowGrayLevelEmphasis	0.880698
**w-LLH**	gldm	HighGrayLevelEmphasis	0.880698
**w-LLL**	firstorder	Kurtosis	0.879676
**w-LLH**	glrlm	ShortRunEmphasis	0.879166
**w-HLL**	glcm	DifferenceEntropy	0.879036
**w-HHL**	glrlm	RunLengthNonUniformity	0.878805
**w-LHH**	gldm	SmallDependenceHighGrayLevelEmphasis	0.878681
**w-LHL**	gldm	HighGrayLevelEmphasis	0.877982
**w-LHL**	gldm	LowGrayLevelEmphasis	0.877982

**w-LHL**	glcm	Autocorrelation	0.877979
**w-LLH**	glcm	Autocorrelation	0.877498
**w-LHH**	glrlm	RunLengthNonUniformityNormalized	0.87745
**w-HHH**	glcm	Idmn	0.876969
**w-HHH**	glcm	Idm	0.876969
**w-HHH**	glcm	Id	0.876969
**w-HHH**	glcm	Idn	0.876969
**w-HHH**	glcm	Contrast	0.876969
**w-HHH**	glcm	InverseVariance	0.876969
**w-HHH**	glcm	DifferenceAverage	0.876969
**w-HLH**	gldm	DependenceVariance	0.876842
**w-HHL**	gldm	DependenceEntropy	0.876649
**w-HHH**	glcm	ClusterProminence	0.876045
**w-LHL**	glcm	ClusterTendency	0.875342
**w-HHH**	glcm	Correlation	0.87514
**w-HHL**	glszm	GrayLevelNonUniformity	0.875046
**w-LLH**	glszm	ZonePercentage	0.874708
**w-LHH**	gldm	LargeDependenceHighGrayLevelEmphasis	0.874539
**w-HHH**	glrlm	ShortRunEmphasis	0.874346
**w-HHL**	gldm	LargeDependenceLowGrayLevelEmphasis	0.874157
**w-HHH**	glrlm	ShortRunHighGrayLevelEmphasis	0.874111
**w-LHL**	ngtdm	Strength	0.873439
**w-HLH**	gldm	LargeDependenceLowGrayLevelEmphasis	0.873224
**w-LHL**	ngtdm	Coarseness	0.871965
**w-LHH**	glszm	GrayLevelNonUniformity	0.871858
**w-HHH**	glcm	ClusterTendency	0.869987
**w-LLH**	gldm	SmallDependenceHighGrayLevelEmphasis	0.869678
**w-LHL**	gldm	LargeDependenceHighGrayLevelEmphasis	0.869309
**w-HHH**	glcm	DifferenceEntropy	0.869011
**w-HHH**	glcm	Imc1	0.868623
**w-HHH**	glszm	GrayLevelNonUniformity	0.868375
**w-LHH**	glrlm	ShortRunLowGrayLevelEmphasis	0.867201
**w-HHH**	glrlm	ShortRunLowGrayLevelEmphasis	0.866636
**Original**	shape	Elongation	0.866275
**w-HLL**	glcm	Imc1	0.866074
**w-LHL**	glszm	ZoneEntropy	0.865821
**w-LHH**	glcm	Idmn	0.864847
**w-LHH**	glcm	Idm	0.864847
**w-LHH**	glcm	Id	0.864847
**w-LHH**	glcm	Contrast	0.864847
**w-LHH**	glcm	InverseVariance	0.864847
**w-LHH**	glcm	DifferenceAverage	0.864847
**w-LHH**	glcm	Idn	0.864847
**w-LHH**	glrlm	ShortRunEmphasis	0.864325
**w-HHH**	glcm	DifferenceVariance	0.864083
**w-HHH**	glcm	Imc2	0.863869

**w-LHH**	glcm	Imc2	0.862529
**w-LHH**	glcm	ClusterProminence	0.8617
**w-HHH**	glcm	JointEntropy	0.86157
**w-LHH**	glcm	Correlation	0.859996
**w-HHL**	glcm	ClusterShade	0.859476
**w-LLH**	glcm	ClusterTendency	0.859385
**w-HHL**	gldm	DependenceVariance	0.857776
**w-HLH**	glcm	ClusterShade	0.857603
**w-HLH**	glcm	DifferenceEntropy	0.857206
**w-HLH**	glcm	DifferenceVariance	0.856924
**w-HHH**	glcm	MaximumProbability	0.856359
**w-HHH**	glcm	JointEnergy	0.853556
**w-LHH**	glrlm	ShortRunHighGrayLevelEmphasis	0.852655
**w-HLH**	glcm	Imc1	0.852243
**w-LHH**	glcm	MaximumProbability	0.852181
**w-HLL**	glszm	HighGrayLevelZoneEmphasis	0.851008
**w-HLL**	glszm	LowGrayLevelZoneEmphasis	0.851008
**w-HHL**	glcm	Autocorrelation	0.850636
**w-HLH**	glcm	Idmn	0.848076
**w-HLH**	glcm	Contrast	0.848076
**w-HLH**	glcm	InverseVariance	0.848076
**w-HLH**	glcm	DifferenceAverage	0.848076
**w-HLH**	glcm	Idm	0.848076
**w-HLH**	glcm	Id	0.848076
**w-HLH**	glcm	Idn	0.848076
**w-LHH**	glcm	DifferenceVariance	0.847832
**w-HHL**	glcm	JointAverage	0.847478
**w-HHL**	glcm	SumAverage	0.847478
**w-HHL**	gldm	LowGrayLevelEmphasis	0.847086
**w-HHL**	gldm	HighGrayLevelEmphasis	0.847086
**w-HLL**	glrlm	RunLengthNonUniformity	0.84702
**w-HHL**	glcm	Imc1	0.845525
**w-LHH**	glcm	ClusterTendency	0.845086
**w-HHL**	glcm	Imc2	0.844046
**w-HHL**	glcm	Correlation	0.843886
**w-LHH**	glcm	DifferenceEntropy	0.8432
**w-HLL**	glrlm	LowGrayLevelRunEmphasis	0.841735
**w-HLL**	glrlm	HighGrayLevelRunEmphasis	0.841735
**w-LHH**	glcm	JointEntropy	0.840591
**w-HLL**	glcm	Correlation	0.84014
**w-HHL**	glszm	ZoneEntropy	0.839566
**w-LHH**	glcm	JointEnergy	0.839438
**w-HLL**	firstorder	Skewness	0.839149
**w-LLH**	glcm	Imc1	0.839042
**w-LHH**	glcm	Imc1	0.838862
**w-HHL**	glcm	DifferenceVariance	0.838839

**w-LHL**	glcm	Imc1	0.83849
**w-LHL**	glcm	Imc2	0.83807
**w-HHL**	glcm	Idmn	0.837586
**w-HHL**	glcm	Idm	0.837586
**w-HHL**	glcm	Id	0.837586
**w-HHL**	glcm	Contrast	0.837586
**w-HHL**	glcm	InverseVariance	0.837586
**w-HHL**	glcm	DifferenceAverage	0.837586
**w-HHL**	glcm	Idn	0.837586
**w-HLL**	glszm	ZoneEntropy	0.83734
**Original**	glrlm	ShortRunLowGrayLevelEmphasis	0.837241
**Original**	glrlm	ShortRunHighGrayLevelEmphasis	0.837241
**Original**	glrlm	ShortRunEmphasis	0.837241
**w-LLL**	glrlm	ShortRunLowGrayLevelEmphasis	0.837241
**w-LLL**	glrlm	ShortRunHighGrayLevelEmphasis	0.837241
**w-LLL**	glrlm	ShortRunEmphasis	0.837241
**w-HHL**	glcm	DifferenceEntropy	0.836719
**w-HLH**	glcm	Autocorrelation	0.836388
**w-HLH**	gldm	HighGrayLevelEmphasis	0.834929
**w-HLH**	gldm	LowGrayLevelEmphasis	0.834929
**w-LLH**	firstorder	Skewness	0.834849
**w-HLH**	glcm	SumAverage	0.834708
**w-HLH**	glcm	JointAverage	0.834708
**w-LHL**	glcm	Correlation	0.833034
**w-HLH**	glcm	Correlation	0.832962
**w-HHL**	glcm	MaximumProbability	0.832198
**w-LLH**	gldm	LargeDependenceHighGrayLevelEmphasis	0.830474
**w-HLH**	glcm	ClusterProminence	0.829748
**w-HHL**	ngtdm	Busyness	0.82953
**w-HHL**	glcm	ClusterProminence	0.827837
**w-LHL**	firstorder	Skewness	0.826883
**w-HLH**	firstorder	Skewness	0.82514
**w-LHL**	glrlm	ShortRunHighGrayLevelEmphasis	0.824135
**w-HLL**	firstorder	Kurtosis	0.821228
**w-HLH**	glcm	Imc2	0.820883
**w-HLL**	glcm	MaximumProbability	0.819009
**w-LHH**	firstorder	Median	0.817134
**w-HHL**	glcm	ClusterTendency	0.81607
**w-HHH**	glszm	ZoneEntropy	0.81598
**w-LLH**	glcm	Correlation	0.812768
**w-HLH**	glcm	JointEntropy	0.812336
**w-HLL**	glcm	JointEntropy	0.812245
**w-HHL**	glcm	JointEntropy	0.811263
**w-HLH**	glcm	MaximumProbability	0.810448
**w-LLH**	glrlm	ShortRunHighGrayLevelEmphasis	0.80945
**w-HHL**	glcm	SumEntropy	0.80713

**w-HLL**	glszm	SizeZoneNonUniformityNormalized	0.805516
**w-HLL**	glcm	JointEnergy	0.805344
**w-HHL**	glcm	JointEnergy	0.805242
**w-LHH**	glszm	ZoneEntropy	0.804995
**w-LHL**	glszm	GrayLevelNonUniformityNormalized	0.803464
**w-LHL**	glszm	GrayLevelVariance	0.803464
**w-LLH**	glcm	Imc2	0.801668
**w-LHH**	glcm	SumEntropy	0.801619
**w-LHH**	firstorder	Mean	0.801335
**w-HLL**	glszm	GrayLevelNonUniformity	0.801119
**w-HLL**	glcm	SumEntropy	0.799829
**w-HLL**	glcm	ClusterTendency	0.798639
**w-HLH**	glszm	ZoneEntropy	0.797541
**w-HLL**	glcm	SumSquares	0.796946
**w-HLH**	glcm	JointEnergy	0.796908
**w-LHH**	glcm	Autocorrelation	0.79603
**w-HLL**	firstorder	Uniformity	0.793951
**w-HLL**	gldm	GrayLevelVariance	0.793951
**w-LHH**	glcm	ClusterShade	0.791307
**w-HHL**	glcm	SumSquares	0.791303
**w-HLH**	glcm	SumEntropy	0.789909
**w-LLH**	firstorder	Kurtosis	0.788565
**w-LHH**	glcm	JointAverage	0.78737
**w-LHH**	glcm	SumAverage	0.78737
**w-HLL**	glcm	Imc2	0.78728
**w-HLL**	firstorder	Entropy	0.786939
**w-HHL**	gldm	GrayLevelVariance	0.785391
**w-HHL**	firstorder	Uniformity	0.785391
**w-HLL**	glszm	SizeZoneNonUniformity	0.781792
**w-LHL**	glszm	HighGrayLevelZoneEmphasis	0.781247
**w-LHL**	glszm	LowGrayLevelZoneEmphasis	0.781247
**w-HHL**	firstorder	Entropy	0.781125
**w-LLH**	glszm	GrayLevelNonUniformityNormalized	0.77928
**w-LLH**	glszm	GrayLevelVariance	0.77928
**w-LHL**	glrlm	LowGrayLevelRunEmphasis	0.774621
**w-LHL**	glrlm	HighGrayLevelRunEmphasis	0.774621
**w-LHH**	gldm	HighGrayLevelEmphasis	0.773615
**w-LHH**	gldm	LowGrayLevelEmphasis	0.773615
**w-HLH**	glcm	ClusterTendency	0.765461
**w-HHL**	glrlm	LowGrayLevelRunEmphasis	0.765018
**w-HHL**	glrlm	HighGrayLevelRunEmphasis	0.765018
**w-HLH**	firstorder	Uniformity	0.764864
**w-HLH**	gldm	GrayLevelVariance	0.764864
**w-HLH**	firstorder	Entropy	0.758967
**w-HLH**	glcm	SumSquares	0.758418
**w-LLH**	glszm	ZoneEntropy	0.755177

**w-LLH**	glrlm	HighGrayLevelRunEmphasis	0.751007
**w-LLH**	glrlm	LowGrayLevelRunEmphasis	0.751007
**w-LLH**	glcm	ClusterShade	0.748799
**w-LLH**	glcm	ClusterProminence	0.7485
**w-LLH**	glrlm	GrayLevelVariance	0.746687
**w-LLH**	glrlm	GrayLevelNonUniformityNormalized	0.746687
**w-HHL**	glrlm	GrayLevelNonUniformityNormalized	0.744576
**w-HHL**	glrlm	GrayLevelVariance	0.744576
**w-HLL**	glszm	SmallAreaLowGrayLevelEmphasis	0.744156
**w-HLL**	ngtdm	Strength	0.743358
**w-HHL**	firstorder	Skewness	0.739503
**Original**	glszm	SmallAreaHighGrayLevelEmphasis	0.737823
**Original**	glszm	SmallAreaEmphasis	0.737823
**Original**	glszm	SmallAreaLowGrayLevelEmphasis	0.737823
**w-LLL**	glszm	SmallAreaHighGrayLevelEmphasis	0.737823
**w-LLL**	glszm	SmallAreaEmphasis	0.737823
**w-LLL**	glszm	SmallAreaLowGrayLevelEmphasis	0.737823
**w-HLL**	glszm	GrayLevelNonUniformityNormalized	0.736502
**w-HLL**	glszm	GrayLevelVariance	0.736502
**w-HLH**	glszm	HighGrayLevelZoneEmphasis	0.735284
**w-HLH**	glszm	LowGrayLevelZoneEmphasis	0.735284
**w-LLH**	glszm	HighGrayLevelZoneEmphasis	0.734415
**w-LLH**	glszm	LowGrayLevelZoneEmphasis	0.734415
**w-HLL**	ngtdm	Busyness	0.729168
**w-HHH**	firstorder	Median	0.728044
**w-LHH**	firstorder	Skewness	0.727553
**w-HHH**	firstorder	Kurtosis	0.726816
**w-LHH**	glszm	SmallAreaEmphasis	0.726359
**w-HHH**	firstorder	Mean	0.723264
**w-HLL**	glrlm	GrayLevelVariance	0.71555
**w-HLL**	glrlm	GrayLevelNonUniformityNormalized	0.71555
**w-HLL**	ngtdm	Coarseness	0.715013
**w-LHL**	glszm	SizeZoneNonUniformityNormalized	0.711486
**w-LHL**	glrlm	GrayLevelNonUniformityNormalized	0.70614
**w-LHL**	glrlm	GrayLevelVariance	0.70614
**w-HHH**	glszm	SmallAreaEmphasis	0.701201
**w-HHL**	firstorder	Kurtosis	0.700476
**w-LHL**	glcm	ClusterProminence	0.699864
**w-LHH**	glcm	SumSquares	0.699192
**w-HHH**	glcm	Autocorrelation	0.693159
**w-HLH**	glszm	SmallAreaHighGrayLevelEmphasis	0.692647
**w-LHH**	firstorder	Kurtosis	0.692634
**w-HLH**	firstorder	Kurtosis	0.688852
**w-HLH**	glszm	SmallAreaEmphasis	0.687777
**w-LHH**	firstorder	Uniformity	0.685661
**w-LHH**	gldm	GrayLevelVariance	0.685661

**w-LHL**	glcm	ClusterShade	0.685066
**w-LHH**	firstorder	Entropy	0.684173
**w-HHL**	glszm	SmallAreaEmphasis	0.683346
**w-HHH**	firstorder	Skewness	0.682535
**w-LHH**	glszm	SizeZoneNonUniformityNormalized	0.681243
**w-LHH**	glrlm	GrayLevelNonUniformityNormalized	0.676606
**w-LHH**	glrlm	GrayLevelVariance	0.676606
**w-HLH**	glszm	SizeZoneNonUniformityNormalized	0.674198
**w-HLH**	glrlm	HighGrayLevelRunEmphasis	0.668203
**w-HLH**	glrlm	LowGrayLevelRunEmphasis	0.668203
**w-HHH**	glcm	ClusterShade	0.663385
**w-HHH**	glrlm	GrayLevelNonUniformityNormalized	0.662268
**w-HHH**	glrlm	GrayLevelVariance	0.662268
**w-LLH**	glszm	SmallAreaHighGrayLevelEmphasis	0.661804
**w-HLL**	glszm	SmallAreaHighGrayLevelEmphasis	0.659879
**w-HHH**	glcm	JointAverage	0.65723
**w-HHH**	glcm	SumAverage	0.65723
**w-LHH**	glszm	SmallAreaHighGrayLevelEmphasis	0.65616
**w-HHH**	glszm	SizeZoneNonUniformityNormalized	0.652089
**w-HLH**	glszm	GrayLevelNonUniformityNormalized	0.650698
**w-HLH**	glszm	GrayLevelVariance	0.650698
**w-HHH**	glcm	SumSquares	0.649986
**w-HHL**	glszm	SmallAreaHighGrayLevelEmphasis	0.649586
**w-HHH**	gldm	LowGrayLevelEmphasis	0.649471
**w-HHH**	gldm	HighGrayLevelEmphasis	0.649471
**w-HLL**	glszm	SmallAreaEmphasis	0.649204
**w-HLH**	glrlm	GrayLevelVariance	0.645295
**w-HLH**	glrlm	GrayLevelNonUniformityNormalized	0.645295
**w-LLH**	glszm	SmallAreaEmphasis	0.645151
**w-HHL**	glszm	LowGrayLevelZoneEmphasis	0.644012
**w-HHL**	glszm	HighGrayLevelZoneEmphasis	0.644012
**w-HHH**	glszm	SmallAreaLowGrayLevelEmphasis	0.64312
**w-HHL**	glszm	SizeZoneNonUniformityNormalized	0.633853
**w-LHL**	glszm	SmallAreaLowGrayLevelEmphasis	0.631572
**w-LHL**	glszm	SmallAreaEmphasis	0.630607
**w-HHH**	gldm	GrayLevelVariance	0.627027
**w-HHH**	firstorder	Uniformity	0.627027
**w-HHH**	firstorder	Entropy	0.626371
**w-HLH**	glszm	SmallAreaLowGrayLevelEmphasis	0.623979
**w-HLH**	ngtdm	Busyness	0.621277
**w-LHL**	glszm	SmallAreaHighGrayLevelEmphasis	0.615952
**w-HHH**	glszm	SmallAreaHighGrayLevelEmphasis	0.604979
**w-LHH**	glszm	SmallAreaLowGrayLevelEmphasis	0.604127
**w-LHH**	glrlm	LowGrayLevelRunEmphasis	0.59799
**w-LHH**	glrlm	HighGrayLevelRunEmphasis	0.59799
**w-HHH**	glrlm	HighGrayLevelRunEmphasis	0.591582

**w-HHH**	glrlm	LowGrayLevelRunEmphasis	0.591582
**w-LLH**	glszm	SmallAreaLowGrayLevelEmphasis	0.589305
**w-HHL**	glszm	SmallAreaLowGrayLevelEmphasis	0.568945
**w-HHL**	glszm	GrayLevelNonUniformityNormalized	0.555566
**w-HHL**	glszm	GrayLevelVariance	0.555566
**w-HLL**	glcm	ClusterProminence	0.551269
**w-LLH**	glszm	SizeZoneNonUniformityNormalized	0.501451
**w-LLH**	ngtdm	Busyness	0.415667
**w-LHL**	ngtdm	Busyness	0.412441
**w-HHH**	glszm	GrayLevelNonUniformityNormalized	0.371009
**w-HHH**	glszm	GrayLevelVariance	0.371009
**w-LHH**	glszm	GrayLevelNonUniformityNormalized	0.31857
**w-LHH**	glszm	GrayLevelVariance	0.31857
**w-HHH**	glszm	LowGrayLevelZoneEmphasis	0.105178
**w-HHH**	glszm	HighGrayLevelZoneEmphasis	0.105178
**w-LHH**	glszm	HighGrayLevelZoneEmphasis	0.075191
**w-LHH**	glszm	LowGrayLevelZoneEmphasis	0.075191
